# Optimized Alkaline Hydrolysis for Recovering Ferulated Arabinoxylan Biopolymers from Maize Bran with Antioxidant Functionality

**DOI:** 10.3390/polym18060689

**Published:** 2026-03-12

**Authors:** Muzzamal Hussain, Kristin Whitney, Senay Simsek

**Affiliations:** 1 Whistler Center for Carbohydrate Research, Department of Food Science, Purdue University, West Lafayette, IN 47907, USA; muzzamalhussain24@gcuf.edu.pk (M.H.); klwhitne@purdue.edu (K.W.); 2 Department of Food Science, Government College University, Faisalabad 38000, Pakistan

**Keywords:** ferulated arabinoxylans, ferulic acid, maize bran, alkaline extraction, gas chromatography–mass spectrometry (GC–MS), high-performance liquid chromatography (HPLC), water absorption, structural properties, industrial applications

## Abstract

Maize bran is an abundant cereal byproduct and a promising source of ferulated arabinoxylan biopolymers (FAXs). In this study, alkaline hydrolysis was optimized for FAX extraction from maize bran using a design-of-experiments approach evaluating alkali concentration, extraction time, and temperature. Purified FAXs were characterized for their chemical composition, phenolic and ferulic acid content, antioxidant activity, microstructure, and functional properties using GC–MS, HPLC, FT-IR, SEM, and standard antioxidant and functional assays. The FAX yields ranged from 14.7 to 18.9%, producing arabinose- and xylose-rich polymers (A/X ratio 0.68–0.74) with a high proportion of bound ferulic acid. Antioxidant assays (FRAP, ABTS, and DPPH) showed that alkaline-extracted and bound phenolic fractions exhibited substantially higher antioxidant capacity (*p* ≤ 0.05) than free phenolics, highlighting the importance of phenolic association with the arabinoxylan backbone. The FAX 3 extract also showed high activity in both the alkaline-extracted phenolic compounds (905.0 μg/g TE) and fraction II (286.5 μg/g TE), indicating that specific structural features may contribute to its bioactivity. In addition, FAXs demonstrated high water-holding capacity and favorable emulsifying properties. These results support the recovery of maize bran-derived FAXs as functional, antioxidant-active ingredients for food and related applications.

## 1. Introduction

Lignocellulosic materials represent the largest biomass resource worldwide. Cereal waste is predominantly lignocellulosic and consists mainly of lignin, cellulose, and hemicellulose, making it suitable for producing food and bioenergy, which is of great importance given limited agricultural areas and the expectation that fossil fuels will become scarcer in the future [1]. Valorization of existing lignocellulosic byproducts offers a sustainable strategy to improve resource efficiency without expanding agricultural land use, while simultaneously reducing dependence on fossil-derived raw materials. In recent years, cereal wastes have attracted considerable attention for their biotechnological utilization, as a majority of their current use is in animal feed [2,3]. Among lignocellulosic components, hemicelluloses such as arabinoxylans are particularly attractive for valorization due to their alkali solubility, structural heterogeneity, and association with phenolic acids that confer functional and bioactive properties.

Maize bran is one of the most abundantly produced, inexpensive lignocellulosic materials worldwide, generated in large quantities as a byproduct of global wet- and dry-milling operations in the starch and cereal industries. It mainly contains testa, pericarp, aleurone layers, and residual sclerenchyma. These outer kernel layers are particularly enriched in arabinoxylans and ester-linked phenolic acids, which underpin the high ferulated arabinoxylan content and extractability of maize bran. Maize bran is an abundant byproduct of the starch industry, either fed to animals or discarded. Nevertheless, maize bran is rich in dietary fibers and phenolic compounds [4,5]. There are complex carbohydrate polymers in maize, including cellulose (20%), hemicellulose (30%), starch (70–85%), protein (10–13%), crude oil (2–3%), and phenolic acids (4%) [6,7]. As for the carbohydrate content in maize bran, the different layers are dominated by polysaccharides, including cellulose, hemicellulose, and lignin. Maize bran contains relatively high levels of arabinoxylan in its outer kernel fractions compared with many other cereal brans, making it an attractive source of this hemicellulosic dietary fiber [8]. As a primary dietary fiber source, arabinoxylans are of interest due to their perceived health benefits [9]. They resist digestion in the small intestine and undergo partial or complete fermentation by the microbiota in the large intestine. In addition to its prebiotic activity, dietary fiber also improves lipid and fat metabolism and reduces the risk of various diseases. As a result of arabinoxylan consumption, the risk of chronic cardiovascular disease, intestinal cancer, type II diabetes, and rheumatoid arthritis is reduced, and pathogenic bacteria are prevented from overgrowing [10,11].

Cateigner-Boutin and Saulnier [12] illustrated that ferulic acid dimers and trimers (ester-linked to the O-5 position of hydroxycinnamic acids), and p-coumaric acid dimers are present in cell wall polysaccharides and can be involved in crosslinking of cereal cell wall polysaccharides. Recent studies have shown that phenolic compounds, particularly ferulic acid, are associated with arabinoxylans. However, the structure of ferulated arabinoxylans, or FAX, in the cell wall has not been clarified [13,14,15]. Polysaccharides are usually extracted from natural sources using conventional extraction methods. There are three main types of extraction methods: aqueous extraction, enzyme extraction, and solvent extraction. In chemical solvent extraction, alkali and acid solutions are used [16]. The chemical solvent extraction methods yielded more arabinoxylans than the enzymatic and aqueous extraction methods. There is evidence that a small portion of cereal bran polysaccharides degrades into low-molecular-weight components during extensive acid hydrolysis [17].

Most structural studies of FAXs have used alkaline solutions to extract matrix polysaccharides [18]. Alkaline extraction of ferulated arabinoxylans (FAXs), in which bran cell wall phenolic acids are attached to arabinoxylans, is used to extract FAXs [19]. Previous literature showed that maize bran contains a high content of biopolymers, FAXs, as compared to other cereal bran. Therefore, a comprehensive study of maize bran is essential for the extraction of FAXs. Unlike prior studies that primarily report isolated extraction yields or compositional features, the present work provides a systematic, DOE-based optimization of alkaline hydrolysis for maize bran ferulated arabinoxylans and integrates extraction performance with detailed chemical, phenolic, antioxidant, and functional property characterization under mild, application-relevant conditions. Despite this growing understanding of phenolic association and extraction chemistry, clear relationships between the alkaline processing conditions, phenolic retention, antioxidant activity, and functional performance of maize bran-derived ferulated arabinoxylans remain insufficiently defined [8].

Despite the abundance of maize bran as a low-value byproduct of the cereal and starch industries, its potential as a source of high-value bioactive biopolymers remains underutilized. FAXs are of particular interest due to their combined dietary fiber functionality, phenolic association, and antioxidant potential; however, reported extraction approaches vary widely, and systematic optimization of alkaline hydrolysis conditions remains limited. In addition, the relationships between the extraction conditions, chemical composition, phenolic retention, antioxidant activity, and functional properties of maize bran-derived FAXs are not well established under mild, industrially relevant processing conditions [17,20].

Therefore, the objective of this study was to optimize the alkaline hydrolysis parameters (alkali concentration, time, and temperature) for the extraction of FAXs from maize bran using a design-of-experiments approach, and to comprehensively characterize the resulting biopolymers in terms of monosaccharide composition, phenolic and ferulic acid content, antioxidant capacity, and functional properties. In this study, the optimal extraction conditions are defined not solely by maximum yield, but by the ability to recover ferulated arabinoxylans under mild, application-relevant conditions while maintaining phenolic association, antioxidant activity, and functional performance. This integrated evaluation aims to provide practical insights into the recovery and potential utilization of maize bran-derived FAXs as functional biopolymers for food and related applications.

## 2. Materials and Methods

### 2.1. Materials

The maize bran was provided by Rafhan Maize Product Co., Ltd., Faisalabad, Pakistan. The maize bran was commercially micronized (particle size < 250 μm) using a milling process. Chemicals were procured from Fisher Scientific by Thermo Fisher Scientific, 168 Third Avenue, Waltham, MA 02451, USA.

### 2.2. Chemical Analysis

The maize bran sample was analyzed to determine the moisture, fat, protein, fiber, and ash content. The methods of the American Association of Cereal Chemists (AACC) [21] were used to determine the moisture (AACC method 44–15), ash (AACC method 08–01), protein (AACC method 46–10), fat (AACC method 30–10), and fiber content (AACC method 32–10), while the total carbohydrates were obtained as follows:

Total carbohydrates = 100% − (% moisture + % crude protein + % ash + % crude fat + % crude fiber).

Insoluble dietary fiber and soluble dietary fiber were determined using an enzymatic assay kit (K-TDFR-100A, Megazyme, Wicklow, Ireland) based on the AACC-I official method 32–07.

### 2.3. Extraction of Ferulated Arabinoxylans

FAXs were extracted from maize bran using different concentrations of potassium hydroxide, time, and temperature modes (Table 1). A 50 g sample of maize bran was dried in a hot-air oven at 65 °C for 6 h and defatted with hexane (1:5 *w*/*v*) by stirring at room temperature. The sample was boiled in deionized water for 1 h to induce starch gelatinization, protein denaturation, and enzyme inactivation.

According to the previous method of Herrera-Balandrano et al. [20], with some modifications, the supernatant was discarded, and the sample was dried for 12 h at 60 °C. For hydrolysis, the sample was suspended in alkaline solution (5, 6, and 7%) the stirred at 150 rpm for different time periods (4, 5, and 6 h) and at different temperatures (25, 30, and 35 °C). The slurry was cooled to room temperature over 1 h. The material was then centrifuged at 5000 rpm for 20 min at 4 °C. The supernatant was collected and precipitated with 70% *v*/*v* ethanol overnight. The precipitated material was recovered by solvent exchange (80% *v*/*v* acetone), yielding the crude extract. For purification, the crude extract was dissolved to obtain a homogeneous aqueous solution (1% *w*/*v*) and precipitated by sequential ethanol-induced precipitation (30 and 60% *v*/*v*). The precipitates were again recovered by the solvent-exchange method and dried in a freeze-drier to obtain purified FAXs. The dried extract was blended and converted into powder form for further characterization.

From the 27 extraction conditions generated by the factorial design, nine representative samples (FAX 1–FAX 9) were selected for comprehensive characterization to capture low, intermediate, and high extraction responses across the experimental design space while maintaining analytical feasibility. In order to evaluate the combined effects of the independent extraction variables on the response (extraction yield), interaction plots were generated.

### 2.4. Yield

The yield of FAXs was calculated by the following Equation:

Yield (%) = W_1_/W_0_ × 100

where W_1_ is the weight of dried AX (g) and W_0_ is the weight of dry bran (g).

### 2.5. Monosaccharides Analysis

FAXs extracted from maize bran were analyzed for neutral sugars. Monosaccharides in the FAX extracts were determined by hydrolysis (1 M H_2_SO_4_, 100 °C, 90 min) and conversion into alditol acetates following Pettolino et al. [22]. The determination of arabinose, xylose, galactose, and glucose was done using gas chromatography–mass spectrometry (GC-MS) using an Agilent 5890 and 5973 Mass Selective Detector (Agilent, Santa Clara, CA, USA). Separation was carried out on a 30 m long DB-5 fused-silica capillary column with a film thickness of 1.0 mm and an inner diameter of 0.25 mm, coated with a methyl polysiloxane polymer phase. In this experiment, nitrogen was used as the carrier gas at approximately 1.5 mL/min. The sample (2 μL) was injected into the GC-MS in electron impact mode at 70 eV. For accuracy, monosaccharide experiments were repeated three times. The arabinose to xylose ratio was estimated by following the formula:

$$Ratio=\frac{Concentration\;of\;Arabinose}{Concentration\;of\;Xylose}$$

### 2.6. Measurement of Protein Content

The protein contents of the FAX samples were determined by a Thermo Fisher Scientific (Waltham, MA, USA) Flashsmart Elemental Analyzer N/Protein instrument. For sample preparation, the sample was dried and pressed into small tins, weighed, and inserted into the autosampler for combustion to quantify the nitrogen. The nitrogen was converted to protein using a conversion factor of 6.25.

### 2.7. Determination of Total Phenolic Content

The total phenolic content was determined according to the method of Kulathunga and Simsek [23] with some modifications. The Folin–Ciocalteu procedure was performed on the extracted material (free-form, alkaline-extracted, acid-extracted, bound-form friction I, and bound-form friction II). The extracted sample (30 μL), along with the Folin–Ciocalteu reagent (150 μL) and 7.5% Na_2_CO_3_ (120 μL), were added to a flat-bottom 96-well plate. The plate was incubated at room temperature for 30 min in the dark. The absorbance was measured at 750 nm in a spectrophotometer. Ferulic acid was used to prepare the standard curve, and the results were expressed as ferulic acid equivalents.

### 2.8. Quantification of Ferulic Acid Content

We used the method of Barberousse et al. [24], with some modifications. The ferulic acid content was quantified by HPLC analysis of the extracted materials (free-form, alkaline-extracted, acid-extracted, bound-form friction I, and bound-form friction II). The sample extracts were used directly after being filtered through 0.45 μm nylon syringe filters before injection (10 μL onto an Agilent 1260 Infinity II HPLC system with a diode-array detector (Agilent Technologies, Santa Clara, CA, USA). Separation was carried out on a Zorbax 300SB-C18 column (150 mm × 4.6 mmi.d., 3.5 μm, 300 Å, Agilent Technologies, Santa Clara, CA, USA). The elution and detection parameters were determined according to the method of Barberousse et al. [24] with some modifications. Chromatographic separation was performed using solvent A (HPLC-grade water containing 0.05% trifluoroacetic acid, TFA) and solvent B (acetonitrile with 0.05% TFA). The gradient program was as follows: 100% solvent A, decreased to 70% A over 10 min, held for 5 min and then returned to 100% A within 3 min and maintained at 100% A for an additional 7 min. The column temperature was maintained at 30 °C with a constant flow rate of 1.0 mL min^−1^. Ferulic acid, used as the external standard, was monitored at 320 nm, while cinnamic acid, serving as the internal standard, was detected at 280 nm.

### 2.9. Determination of Antioxidant Activity

#### 2.9.1. ABTS

The reaction was initiated by mixing ABTS (7 mM) with potassium persulfate (2.45 mM) and then the mixture was allowed to stand in the dark at room temperature for 12 h. Mixing of the ABTS reagent (1 mL) with absolute ethanol (75 mL) was carried out the next day. Following this, absolute ethanol was added to the radical solution to dilute it to an absorbance of 0.7 at 750 nm. In the next step, the sample phenolic extract (20 µL) was mixed with the ABTS solution (230 µL) and allowed to stand in the dark for 30 min before the absorbance was measured at 734 nm. The results are reported as microgram per gram of Trolox equivalents (μg/g TE).

#### 2.9.2. FRAP

In this procedure, a FRAP assay was used to measure the reducing power of the polyphenolic extracts. Briefly, TPTZ (2,4,6- tripyridyl-s-triazine) and FeCl_3_ · 6H_2_O (iron (III) chloride hexahydrate) were dissolved in hydrochloric acid and deionized water before analysis. The FRAP working solution is prepared fresh each time and kept out of the light. After mixing the extracted sample with the FRAP reagent (280 μL), the mixture was allowed to stand in the dark for 30 min. In a microplate reader (Thermo Electron, Waltham, MA USA), samples were read at 595 nm and compared to a standard curve after 30 min. Results were reported as microgram per gram of Trolox equivalents (μg/g TE).

#### 2.9.3. DPPH

The extracts were evaluated for antioxidant activity using the DPPH assay according to Brand-Williams et al. [25], with some modifications. The DPPH radical was prepared by mixing the DPPH reagent (2 mg) with 80 μL of methanol. In this experiment, the absorbance of the solution was adjusted to 0.7 at 515 nm. A microplate reader (Thermo Electron, Vantaa, Finland) was used to measure the absorbance of the samples at 515 nm. The results were calculated using the following formula and the results are reported as microgram per gram of Trolox equivalents (μg/g TE).

DPPH radical scavenging activity (%) = 1 − (A sample − A control/A blank) × 100%

### 2.10. Fourier-Transform Infrared Spectroscopy

The Fourier-transform infrared (FT-IR) spectroscopy analysis on the FAX powder samples was recorded on a Thermo-Nicolet 6700 Series FTIR (Thermo Fisher, Waltham, MA, USA). FTIR spectroscopy was used to identify the characteristic absorption bands of the functional groups present in the FAXs. The examined sample was scanned by infrared spectroscopy, during which a computer-connected detector identified it as a continuous wave, reported its spectrum, and assessed its functional units. The lyophilized FAXs were ground into powder using a mortar and pestle. The powdered samples (∼30 mg) were analyzed. The FT-IR spectra were recorded directly using an ATR diamond crystal at a 2 cm^−1^ scan resolution over the 4000–500 cm^−1^ wave number range.

### 2.11. Scanning Electron Microscopy Imaging

The FAX powder samples were analyzed by scanning electron microscopy. For this purpose, a field-emission scanning electron microscope (FEI Teneo Volumescope SEM, Thermo Fisher Scientific, Hillsboro, OR, USA) was used to obtain images. The surface morphology was studied using scanning electron microscopy with a high-intensity electron beam in high-vacuum mode. The samples were analyzed with a platinum coating at low voltage (5 kv). The images were obtained in secondary and backscattered electron modes at magnifications of 2500× and 5000×, respectively.

### 2.12. Functional Properties of Ferulated Arabinoxylans

#### 2.12.1. Determination of Water-Holding Capacity (WHC)

The water-holding capacity (WHC) of the FAXs isolated from maize bran was determined according to the AACC Approved Method 88-04 [26] and Kaur et al. [27], with some modifications. Briefly, the FAXs (0.5 g) were placed in a centrifuge tube with a screw cap, and 24.5 mL of distilled water was added to each tube. The samples were sheared for 2 min at 10,000 rpm and 1 min at 15,000 rpm with a high-speed polytron. Then the tubes were shaken at 100 rpm for 24 h at room temperature (21–22 °C) and centrifuged at 2000 rpm for 15 min. The excess water from the centrifuge tubes was removed by decanting, then the tubes were inverted and each tube was weighed.

The WHC of the sample was calculated using the following equation:

WHC = g_water_/g_sample_ × 100

where g_water_ (g) represents the weight of water held by the sample, and g_sample_ (g) is the original weight of the dry sample.

#### 2.12.2. Oil-Holding Capacity

The oil-holding capacity (OHC) was determined according to Robertson et al. [28] with some modifications. Commercial soybean oil (25 mL) was added to 250 mg of dry sample, stirred, and left at room temperature for 1 h. After centrifugation, the residue was weighed. The oil-holding capacity was expressed as g of oil per g of sample.

OHC = g_oil_/g_sample_ × 100

where g_oil_ (g) represents the weight of oil held by the sample, and g_sample_ (g) is the original weight of the dry sample.

#### 2.12.3. Emulsifying Activity and Emulsion Stability

Emulsifying activity and emulsion stability were evaluated according to Gannasin et al. [29] with some modifications. A homogenizer was used to homogenize a 2% (*w*/*v*) sample suspension in water at 11,000 rpm for 30 s. Then, sunflower oil (100 mL) was added and homogenized for another 1 min. The emulsions were centrifuged in 15 mL graduated centrifuge tubes at 1200× *g* for 5 min, and the volume of the emulsion left was measured. To determine the emulsion stability, emulsions were prepared as described above, heated at 80 °C for 30 min in an oven, cooled to room temperature, and centrifuged at 1200× *g* for 5 min. The emulsifying activity and emulsion stability were calculated using the following equations:

$$Emulsifying\;Activity=\frac{Volume\;of\;emulsified\;layer\;(mL)}{Volume\;of\;whole\;layer\;in\;the\;centrifuge\;tube\;(mL)}\times 100$$

$$Emulsion\;Stability=\frac{Volume\;of\;remaining\;emulsified\;layer\left(mL\right)}{Original\;emulsion\;Volume\left(mL\right)}\times 100$$

### 2.13. Statistical Analysis

Minitab statistical software 2022 was used to conduct a DOE experimental design on the extraction of FAXs from maize bran by optimizing the following different modes: (1) concentration of KOH, (2) time, and (3) temperature to obtain the maximum yield. Twenty-seven treatments were produced by the DOE, as shown in Table 1. Each treatment was repeated three times. For comparison, bioactivities such as the phenolic content, antioxidant activity, and functional properties were measured, but were not included in the DOE response variables. Despite the limitations of this design, the DOE focused primarily on selecting the optimal extraction conditions. In addition, the characterization data obtained for each parameter were analyzed using mean squares and standard deviations, and the lettering of each parameter was conducted using the least significant difference (LSD). In the chemical characterization analysis, all trials were carried out in triplicate, except ferulic acid quantification by HPLC, which was not replicated.

## 3. Results and Discussion

### 3.1. Chemical Composition of Maize Bran

The chemical composition of maize bran is presented in Supplementary File S1 (Table S1). According to the current results, maize bran contains 59.4 ± 0.04% total carbohydrates and 57.3 ± 1.5% total dietary fiber, a 5.8 ± 0.04% soluble dietary fiber fraction, and a 51.9 ± 1.4% insoluble dietary fiber fraction. In addition, maize bran showed a 10.2 ± 0.04% moisture, a 8.2 ± 0.03% crude protein, a 7.5 ± 0.01% crude fiber, a 4.2 ± 0.02% crude fat, and a 1.4 ± 0.01% ash content. These results are consistent with the previous study of Herrera-Balandrano et al. [20], who reported that maize bran’s chemical composition showed a content of 7.47% moisture, 7.6% protein, 1.22% ash, 0.34% fat, and 13.40% crude fiber and a total carbohydrate content of 69.97%, an insoluble dietary fiber content of 54.9%, and a soluble dietary fiber content of 4.4%. However, this difference in the chemical composition of maize bran was due to varying milling methods or cultivation regions.

### 3.2. Extraction Yield of FAXs from Maize Bran

The extraction yields of FAXs from maize bran using different KOH concentration (alkaline solution), time, and temperature modes are shown in Table 1. Significant differences (*p* < 0.05) were observed in the FAX 2, FAX 3, FAX 5, FAX 8, and FAX 9 treatments due to differences in concentration, temperature, and time among the AX yields based on the alkaline method. However, the average yield of FAX 2 (18.9%) recovered from maize bran was higher than FAX 1 (14.8%), FAX 3 (16.2%), FAX 4 (15.2%), FAX 5 (16.1%), FAX 6 (14.7%), FAX 7 (15.8%), FAX 8 (16.3%), and FAX 9 (16.1%), respectively. In a previous study, Martínez-López et al. [30] reported that the FAX yield was 18% in nixtamalized maize bran. According to a previous study by Chanliaud et al. [31], the alkali type (sodium, potassium, or calcium hydroxide) significantly affected the yield. Still, the exact role of the cation remains unclear. Due to the rapid solubilization, the extraction time had little effect on the yield.

The following linear model equation describes the extraction yield of FAXs:

(1) $$Y_{FAX}=\beta_{0}+\beta_{1}X_{1}+\beta_{2}X_{2}+\beta_{3}X_{3}+\beta_{12}X_{1}X_{2}+\varepsilon$$

In this equation, X_1_ represents KOH concentration, X_2_ represents extraction time, and X_3_ represents extraction temperature, respectively. For the values of the equation, Y_FAX_ is the response, X_1_, X_2_, and X_3_ are the three experimental factors included in the model, β_0_, β_1_, β_2_, and β_3_, are the regression coefficients that are estimated from the data, and *ε* is a random variance. However, β_12_ represents the interaction coefficient between the KOH concentration and time. This is a simple polynomial model and describes only the linear relationship between the factors and the response (Equation (1)).

Table 2 presents the regression coefficient estimates for the factorial DOE analysis. The intercept represents (β_0_ = 16.048) the predicted FAX yield when all factors are at the corresponding reference or center levels. Among the individual factors, extraction time (X_2_) had a significant negative effect on yield (β = −2.68, *p* < 0.05), indicating that longer extraction periods reduced FAX recovery under the experimental conditions. Extraction temperature (X_3_) had a positive effect, which was marginally significant (β = 3.37). This suggests that moderate heating can enhance the release of polysaccharides. KOH concentration (X_1_) had a negative coefficient (β = −0.062) with a non-significant *p*-value, implying minimal impact. The interaction term between KOH concentration and extraction time (X_1_ × X_2_) was found to have an almost-zero coefficient (β ≈ 0), implying the two variables operate independently. Based on the regression coefficient estimates in Table 2, extraction time showed a statistically significant negative effect on FAX yield (*p* < 0.05), suggesting that prolonged extraction reduces yield up to the level studied. Temperature contributed positively but marginally, while KOH concentration and the interaction between them were not significant. Each variable contributes to the extraction process in a different way depending on the sign and magnitude of the coefficients. However, this factorial screen study found that extraction time and temperature are the most influential parameters for optimizing FAX recovery. ANOVA model for factorial screen study of extraction parameters on extraction yield shown in Table S2 (Supplementary File S1).

**Figure 1:**
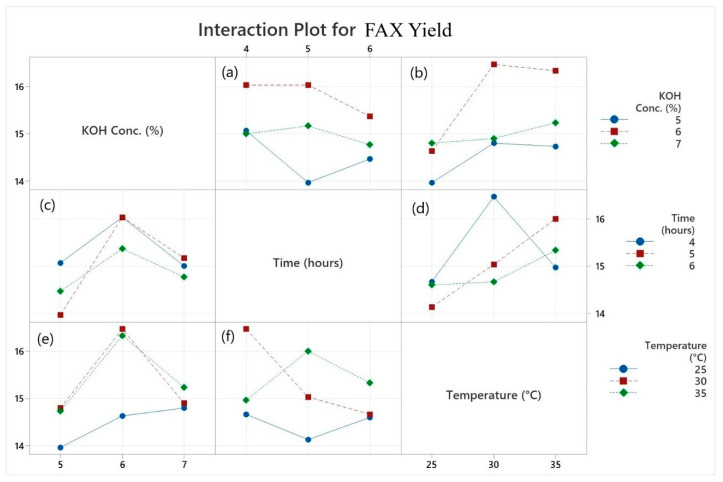
Interaction plot of alkaline concentration, temperature, and time for the extraction yield of ferulated arabinoxylans (FAXs) from maize bran. FAX; Ferulated arabinoxylans, (**a**): Interaction between time and the concentration of KOH, (**b**): Interaction between temperature and the concentration of KOH, (**c**): Relationship between KOH concentration and time, (**d**): Interaction between temperature and time, (**e**): Relationship between concentration of KOH and temperature, (**f**) Interaction between time and temperature.

The general factorial regression of FAX yield versus the treatments of KOH conc. %, time (hours), and temperature are shown in Supplementary File S1. The interaction plot of alkaline concentration, temperature, and time for the extraction yield of FAX from maize bran is shown in Figure 1. The plot shows an interaction plot of the input variables selected based on the extraction yield. Figure 1 provides the interplay among the control factors to increase the yield. The yield, plotted on the vertical axis, is shown as a function of the control factors along the horizontal axis. In Figure 1a, the chart shows the interaction between time and KOH concentration. Figure 1b illustrates the interaction between temperature and the concentration of KOH. At the same time, Figure 1c signifies the relationship between KOH concentration and time. Figure 1d elucidates the connection between temperature and time. Figure 1e signifies the relationship between the concentration of KOH and temperature. At the same time, Figure 1f illustrates the interaction between time and temperature. In these figures, a consistent pattern emerges. Notably, no instances of parallel lines are observed throughout the figures. This observation strongly implies a significant relationship among all the factors and their collective impact on FAX yield. To achieve a higher yield, certain combinations appear to be particularly advantageous. A parallel straight line does not reveal any interaction effects. At the same time, nonparallel lines display an interactional relationship. There is no discernible interaction between the KOH concentration and other parameters, and parallel lines for all values are shown in the interaction plot. However, there is considerable interaction among the 5% KOH solution, the 25 °C temperature, and other input parameters, and no parallel-line relationship exists among them. At all chosen levels, the temperature, time, and KOH solution treatment values show substantial variable interactions.

In this study, analyses of the monosaccharide composition and protein and phenolic content are presented as indicators of chemical composition and phenolic association relevant to functionality, rather than as a comprehensive structural characterization of arabinoxylans.

### 3.3. Protein Content

The protein content of the FAXs extracted from maize bran ranged from 0.96% to 3.58% (Table 3). The maximum protein content was found in FAX 1 (3.58 ± 0.05%) followed by protein contents of 2.86 ± 0.06, 3.37 ± 0.05, 0.96 ± 0.04, 1.23 ± 0.09, 1.51 ± 0.02, 2.09 ± 0.04, 2.01 ± 0.08 and 1.8 ± 0.06% found in FAX 2_,_ FAX 3_,_ FAX 4, FAX 5, FAX 6, FAX 7, FAX 8_,_ and FAX 9_,_ respectively. The current results showed that FAX 1 and FAX 3 had a significantly (*p* < 0.05) higher protein content than the other treatments. The results of the current study are higher than the data reported in a previous study by Herrera-Balandrano et al. [20], who revealed that the protein contents of feruloylated arabinoxylan extracts were 1.00%, 0.86%, and 0.63% under alkaline conditions (0.5 N NaOH) at various times of 2 h, 4 h, and 6 h, respectively.

### 3.4. Monosaccharides

FAXs were extracted from maize bran using an alkaline method with varying concentrations, temperatures, and time conditions. The arabinose, xylose, galactose, and glucose content and Ara/Xyl ratios for the FAXs obtained from different extraction conditions are shown in Table 3. The FAXs showed variations in their monosaccharide composition under different extraction conditions. As a result of the most effective combination of alkaline concentration, temperature, and time, significantly (*p* ≤ 0.05), FAX 4 exhibited the highest levels of arabinose (30.4 ± 0.86%) and xylose (44.7 ± 2.49%). These results indicated that optimized extraction conditions maximized the solubilization of these sugars, which contribute to the functional properties of arabinoxylans. However, FAX 2 showed the lowest arabinose (12.7 ± 1.74%) and xylose (18.3 ± 2.63%) concentrations under less-effective extraction conditions. To maximize yields, optimization of the extraction parameters is vital. In most samples, the Ara/Xyl ratio ranged from 0.68 to 0.74, indicating a balanced presence of arabinose and xylose. The polysaccharides must have these ratios to remain structurally and functionally stable. The galactose and glucose concentrations were significantly lower than those of arabinose and xylose in all treatments (*p* < 0.05). However, FAX 4 exhibited significantly (*p* ≤ 0.05) higher galactose (7.4 ± 0.39%) and glucose (9.2 ± 0.39%) concentrations. As a result of the arabinose and xylose ratio, arabinoxylans may be suitable for a variety of food applications due to their gelling and absorption properties [32].

Alkaline methods are used to extract FAXs from maize bran, demonstrating that extraction conditions meaningfully influence monosaccharide composition and yield [33]. It is possible to increase arabinose and xylose recovery by optimizing these parameters, resulting in higher yields of functional arabinoxylans with potential applications in the food and nutraceutical industries [9]. It is essential to investigate the functional characterization of these extracts in the future to better understand their potential health benefits and applications.

### 3.5. Total Phenolic Content

The current results regarding total phenolic content (TPC) in FAX treatments are presented in Table 4. In the current study, TPC was determined for free-form phenolic compounds and for alkali-extractable, acid-extractable, and bound-form (fractions I and II) phenolic compounds from maize bran FAXs. Free-form phenolics are those that are not covalently bound through ester linkages to the arabinoxylan backbone. These results confirmed that the alkaline extract showed higher TPC than the other fractions, while the FAXs contained lower free-form TPC. FAX 3 had significantly (*p* < 0.05) higher free-form (114.4 ± 1.76 μg/g FAE), alkaline- (978.8 ± 2.7 μg/g FAE), and acid-extracted (347.6 ± 0.22 μg/g FAE) TPC than the other treatments. In fraction I, bound-form TPC, FAX 1 showed a significantly (*p* < 0.05) higher amount (107.2 ± 0.48 μg/g FAE) compared to other treatments. In contrast, FAX 7 showed significantly (*p* < 0.05) higher TPC (292.8 ± 3.09 μg/g FAE) from the bound fraction II compared to other treatments.

Based on the results presented in Table 4, there are significant (*p* < 0.05) differences in the total phenolic content of FAXs extracted from maize bran depending on the extraction method and treatment. TPC yields were highest with alkaline extraction, indicating this method is more effective at liberating phenolic compounds than acid and free extraction methods. Alkaline conditions may enhance the solubility and extraction efficiency of phenolic compounds by disrupting cell wall structures, as suggested by previous studies [34,35,36]. A prior study by Huang et al. [37] demonstrated a significant increase in TPC in cereal byproducts, underscoring the potential of alkaline extraction to optimize phenolic extraction. The current study contributes to the understanding of how extraction methods and treatment variations influence phenolic compounds in maize bran, reinforcing the importance of optimizing extraction protocols to maximize TPC yields. Further studies may be conducted to investigate the bioactivity of these phenolic compounds and their potential health benefits.

### 3.6. Ferulic Acid Content

Free-form, alkaline, acidic, and bound fractions I and II phenolic compound extracts were used to measure ferulic acid content in FAX samples obtained from maize bran. A summary of these results is presented in Table 5, and HPLC graphs are shown in Supplementary File S3. Ferulic acid content varies significantly (*p* < 0.05) between different treatments and extraction methods. As for free-form ferulic acid, FAX 3 had the highest amount (44.71 μg/g), while FAX 5 and FAX 7 had the lowest, at 0.34 μg/g and 0.44 μg/g, respectively. The concentration of ferulic acid in the alkaline extract of FAX 3 was significantly higher (*p* < 0.05) than in any other treatment and was 267.88 μg/g. The acid extracts generally showed lower ferulic acid content; FAX 3 showed significantly higher (*p* < 0.05) ferulic acid content (11.62 μg/g) than the other treatments, while FAX 4 had the lowest content (1.78 μg/g). In the bound fraction I, FAX 1 showed the highest ferulic acid content (3.35 μg/g).

In contrast, in the bound fraction II, FAX 7 showed the highest concentration, at 127.32 μg/g. Bound fractions I and II contain ferulic acid, suggesting that bound forms are present in maize bran FAXs, which can be extracted to free this compound. On the other hand, free-form ferulic acid levels were generally low, especially in treatments FAX 5 and FAX 7. In this case, ferulic acid is mostly bound in maize bran and could be released through appropriate extraction techniques. In bound fraction II, FAX 7 had a significantly (*p* < 0.05) higher bound ferulic acid content (127.32 μg/g), indicating that some treatments can enhance the retention of these compounds. Further research is needed to examine their bioavailability and health benefits. It is clear from these findings that optimal extraction methods are crucial for maximizing the health-promoting properties of maize bran, as they provide a more comprehensive understanding of how they affect the yield of beneficial compounds, such as ferulic acid.

The results of this study demonstrate that different extracts had significantly different levels of ferulic acid content in FAXs from maize bran (*p* < 0.05). The alkaline-extractable phenolic compounds contained the highest levels of ferulic acid. FAX 3 showed a superior ferulic acid content, indicating its potential as a valuable source of phenolic compounds. Based on the previous literature, Wang et al. [19] reported that alkaline conditions disrupt cell wall structures and solubilize phenolic compounds, leading to higher concentrations of ferulic acid in alkaline extracts. A previous study by Bauer et al. [38] confirmed that ferulic acid is the primary phenolic compound in corn fiber and wheat bran. According to the findings of this study, high amounts of bound ferulic acid were released during the alkaline hydrolysis of corn fiber and wheat bran. The stability of ferulic acid against oxidative damage was not affected by alkaline hydrolysis.

### 3.7. Antioxidant Activity

In this study, phenolic compounds in maize bran FAXs were assessed using three assays: FRAP, ABTS, and DPPH. A summary of the results is presented in Table 6. Each extraction treatment produced a different level of antioxidant activity, revealing that the extraction method and the form of the bioactive compounds exert substantial influences on antioxidant activity.

Based on the FRAP results, the bound forms of arabinoxylans exhibit significantly (*p* < 0.05) higher antioxidant activity than the free forms, particularly when extracted using alkaline methods. Alkaline-extracted phenolic compounds from FAX 1 yielded 894.7 μg/g TE, whereas its free-form phenolic compounds yielded only 56.5 μg/g TE. According to this trend, bound forms have enhanced antioxidant properties due to their structural properties. The FAX 3 extract also showed high activity in both the alkaline-extracted phenolic compounds (905.0 μg/g TE) and fraction II (286.5 μg/g TE), indicating that specific structural features may contribute to its activity.

FAX 3 also showed superior antioxidant activity in the ABTS assay with a value of 428.7 μg/g TE in its free form. These findings indicated that FAX 3 again demonstrated superior antioxidant activity in the ABTS assay compared to other treatments. Generally, the bound forms retained substantial antioxidant activity, with the alkali-extractable phenolic compounds from FAX 6 reaching 705.7 μg/g TE. Due to their polyphenolic content and structural characteristics, FAXs appear to possess potent radical scavenging properties. In the DPPH assay, FAX 1 and FAX 3 showed notable antioxidant activity in their bound forms, with values of 511.5 and 560.1 μg/g TE, respectively. A significant (*p* < 0.05) difference was observed between the bound and free forms of the compounds, highlighting the effectiveness of bound compounds as antioxidants. Among all treatments, FAX 4 had the lowest DPPH activity, possibly due to reduced polyphenol content.

Based on these results, it is evident that FAXs extracted from maize bran have comparable antioxidant activity to other plant-derived antioxidants [32]. According to previous studies, the antioxidant capacity of bound phenolic compounds was significantly enhanced by their structural integrity and interactions with other compounds [39]. In addition to their antioxidant activity, bound FAXs have potential applications in food preservation and nutraceuticals. As a result of the superior activity of alkaline-extracted fractions, extraction methods must be optimized so that greater amounts of beneficial compounds can be recovered. In the future, researchers should investigate the mechanisms underlying the antioxidant activity of these FAXs and the potential health benefits associated with their consumption.

Alkaline hydrolysis facilitates the disruption of maize bran cell wall architecture by cleaving ester linkages between phenolic acids and hemicellulosic chains and by partially loosening hydrogen-bonded polysaccharide networks, thereby enhancing the solubilization of FAXs. Under the mild alkaline conditions applied in this study, ferulic acid remains predominantly associated with the arabinoxylan backbone rather than undergoing extensive degradation, which helps preserve phenolic functionality while increasing extractability. The observed increases in bound phenolic content and antioxidant activity are therefore consistent with improved accessibility of feruloylated sites and reduced steric constraints within the polysaccharide matrix. These structural and compositional changes also contribute to enhanced water-holding and emulsifying properties by promoting polymer hydration and interfacial activity, linking the extraction conditions directly to both bioactivity and functional performance.

### 3.8. Fourier-Transform Infrared (FT-IR) Spectroscopy

**Figure 2:**
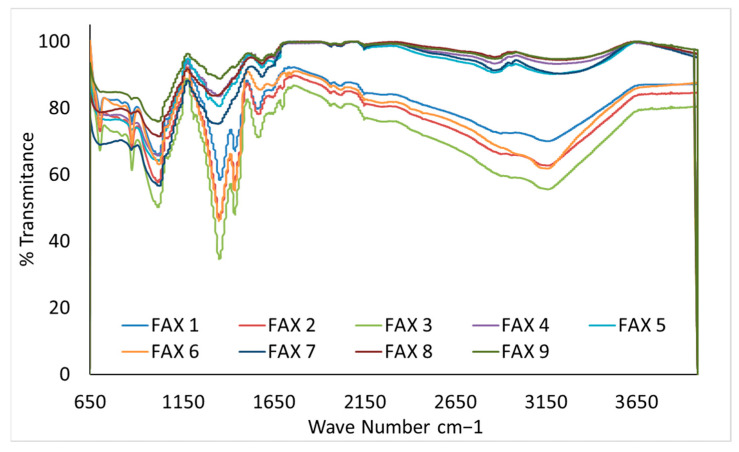
Infrared spectrograms of different treatments of ferulated arabinoxylans extracted from maize bran. FAX 1: KOH concentration 5%, time 4 h, temperature 25, 30 and 35 °C, FAX 2: KOH concentration 5%, time 5 h, temperature 25, 30 and 35 °C, FAX 3: KOH concentration 5%, time 6 h, temperature 25, 30 and 35 °C, FAX 4: KOH concentration 6%, time 4 h, temperature 25, 30 and 35 °C, FAX 5: KOH concentration 6%, time 5 h, temperature 25, 30 and 35 °C FAX 6: KOH concentration 6%, time 6 h, temperature 25, 30 and 35 °C, FAX 7: KOH concentration 7%, time 4 h, temperature 25, 30 and 35 °C, FAX 8: KOH concentration 7%, time 5 h, temperature 25, 30 and 35 °C, FAX 9: KOH concentration 7%, time 6 h, temperature 25, 30 and 35 °C.

FTIR spectroscopy was used as a confirmatory, qualitative technique to identify the functional groups and characteristic glycosidic linkages associated with FAXs. FTIR enables the qualitative identification of functional groups. However, HPLC and GCMS were employed to quantitatively determine the ferulic acid content, antioxidant activity, and neutral sugar levels. The spectra primarily demonstrate the preservation of the AX backbone and associated functional groups across extraction treatments. While minor variations in band intensity were observed, FTIR was not applied for quantitative comparison among samples, and no major structural alterations were detected as a function of the extraction conditions. Figure 2 shows the infrared spectrograms of the FAX samples. In this study, FT-IR spectroscopy was used as a supplementary technique to explore the characteristic organic groups of polysaccharides. The FTIR spectra in the range 900–1300 cm^−1^ are characteristic of functional groups in polysaccharides and nucleic acids. In the current study, the spectra 1050–1150 cm^−1^ are indicative of the glycosidic linkages in polysaccharides. The spectra in this range most probably correspond to polysaccharides. The FTIR spectra obtained from the FAX powder samples extracted from maize bran are shown in Supplementary File S2 (Figure S1 FAX1 to FAX9). According to Supplementary File S1, the FTIR results revealed a similar chemical structure, and the different extraction methods did not change their molecular identity. However, the corresponding bands observed in the FAX samples showed some differences. In general, the corresponding peak band at 704 cm^−1^ is observed in FAX 1, FAX 2, FAX 3, FAX 6, FAX 7, and FAX 8, indicating the polymer backbone.

FAX 9 exhibited a band at 710 cm^−1^, while FAX 5 and FAX 4 showed bands at 761 and 708 cm^−1^, respectively. All samples (FAX 1–FAX 9) displayed characteristic bands at 878–881 cm^−1^. Peaks observed at 1024–1032 cm^−1^ across all samples correspond to glycosidic and (1 → 4) linkages between sugar monomers, reflecting C–OH bending and C–O–C glycosidic bond vibrations. The principal band at 1024 cm^−1^ is assigned to C–OH bending, and strong C–O stretching vibrations are typical of xylose-based polysaccharides. The arabinose side chain at the O-3 position of xylose produced bands at 990–1020 cm^−1^, with decreasing intensity in the 990–1150 cm^−1^ region as the Ara/Xyl ratio increased. The characteristic AX region (1200–900 cm^−1^) includes a maximum absorption band near 1045 cm^−1^ (C–OH bending), a band at 1163 cm^−1^ related to asymmetric C–O–C stretching, and the α-(1 → 4) linkage band at 898 cm^−1^. The low intensity of the band at 1078 cm^−1^ is associated with a high degree of arabinose substitution at the C3 position of xylose. For gel structures, a broad triad of bands at 1112, 1045, and 994 cm^−1^ is attributed to C–C and C–O bending in alcohol groups, while weaker C–O–H bending vibrations decrease in intensity at 1357–1365 cm^−1^.

Bands at 1550 and 1670 cm^−1^ correspond to amide II and amide I (proteins), respectively, while phenolic acids exhibit characteristic absorption in the 1500–1800 cm^−1^ range [40]. In samples FAX 1 to FAX 9, signals between 1625 and 1542 cm^−1^ are associated with the carbonyl stretching of amide I and N–H stretching of amide II, with the band at 1625 cm^−1^ attributed to asymmetric stretching of carboxyl C=O bonds. In the region 1600–1650 cm^−1^, there are IR peaks corresponding to aromatic C–C stretching, indicating the phenolic moiety of ferulic acid, which is covalently bonded to arabinoxylan. The band at 1422 cm^−1^ corresponds to the symmetric C–O stretching of uronic acid groups in AX. The absorption peak at 2925 cm^−1^ is associated with methylene groups. The FTIR spectra of crosslinked materials indicate that the FAX polymer backbone is not significantly altered by crosslinking between arabinoxylans and ferulic acid [41]. Broad bands at 3272–3281 cm^−1^ correspond to O–H stretching vibrations, while bands near 2922 cm^−1^ are attributed to CH_2_ groups. Overall, the FT-IR data confirm that the extracted biopolymer is a ferulated arabinoxylan. It has both polysaccharide backbone features and phenolic ester substitutions. These structural features are important for its antioxidant activity, crosslinking potential, and functional properties in food and nutraceutical applications. The characteristic FTIR wave numbers and associated functional groups are summarized in Table 7.

### 3.9. Scanning Electron Microscopy

**Figure 3:**
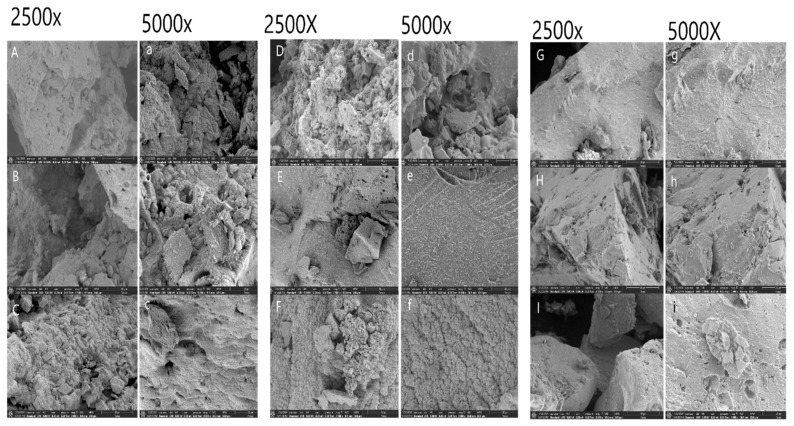
SEM images of ferulated arabinoxylans extracted from maize bran at magnifications of ×2500 and ×5000. (**A**,**a**) = FAX 1, (**B**,**b**) = FAX 2, (**C**,**c**) = FAX 3, (**D**,**d**) = FAX 4, (**E**,**e**) = FAX 5, (**F**,**f**) = FAX 6, (**G**,**g**) = FAX 7, (**H**,**h**) = FAX 8, and (**I**,**i**) = FAX 9. FAX 1: KOH concentration 5%, time 4 h, temperature 25, 30 and 35 °C, FAX 2: KOH concentration 5%, time 5 h, temperature 25, 30 and 35 °C, FAX 3: KOH concentration 5%, time 6 h, temperature 25, 30 and 35 °C, FAX 4: KOH concentration 6%, time 4 h, temperature 25, 30 and 35 °C, FAX 5: KOH concentration 6%, time 5 h, temperature 25, 30 and 35 °C, FAX 6: KOH concentration 6%, time 6 h, temperature 25, 30 and 35 °C, FAX 7: KOH concentration 7%, time 4 h, temperature 25, 30 and 35 °C, FAX 8: KOH concentration 7%, time 5 h, temperature 25, 30 and 35 °C, FAX 9: KOH concentration 7%, time 6 h, temperature 25, 30 and 35 °C.

Scanning electron microscopy was employed to qualitatively assess the surface morphology of FAXs extracted under different alkaline conditions. The SEM images were used to visualize the general structural features characteristic of FAXs, including the porosity, fibrillar networks, and surface roughness, rather than to provide quantitative or treatment-to-treatment morphological comparisons. The SEM micrographs at magnifications of ×2500 and ×5000 (Figure 3) revealed that all FAX samples consisted of thin filamentous structures intertwined with irregular, sponge-like domains, resulting in a loose and porous microstructure.

While no pronounced or systematic morphological differences were observed among treatments, FAX 4 and FAX 6 exhibited more evident sponge-like networks with larger voids, which is consistent with their higher water-holding capacity measured in Section 3.10. The presence of large gaps and porous regions likely enhances water penetration and polymer hydration, thereby contributing to improved swelling and solubility behavior. These observations align with the functional data and support the role of microstructural openness in governing hydration-related properties.

The observed porous and irregular morphologies are consistent with the effects of alkaline extraction, which can partially disrupt hydrogen bonding and polysaccharide associations, leading to fragmentation and loosening of the arabinoxylan network [53]. Although subtle variations in surface roughness and filament organization were noted—particularly the more compact, filament-dominated morphology of FAX 5—these differences did not show a clear quantitative correlation with the extraction conditions. Overall, the SEM results provide qualitative structural context that complements the chemical composition and functional property data, supporting the relationship between alkaline-induced microstructural loosening and the observed water-holding and emulsifying behaviors of FAXs. The high water-holding capacity and emulsifying performance observed for the FAXs are consistent with the porous, loosely organized microstructures revealed by SEM analysis (Section 3.9), in which increased surface area and open filamentous networks facilitate polymer hydration and interfacial interactions.

### 3.10. Functional Properties

The ferulated arabinoxylan fraction is wholly composed of dietary fiber arabinoxylans, protein, and phenolic compounds (Ferulic acid), and thus could be effectively used as a non-caloric texturizing and bulking agent in health-promoting food products designed for weight loss and controlling diabetes [9,54]. Table 8 shows the water-holding capacity of FAXs extracted from maize bran.

The water-holding capacity depends on the branching, molecular structure of the fiber and the carbohydrate composition. The water-holding capacity of cellulose-rich fractions has been attributed to their highly branched, porous nature. All treatments of FAXs showed high water-holding capacities ranging from 8.2 to 9.7 g/g. FAX 4 showed the highest water-holding capacity compared to other treatments. The variation in water-holding capacity among different FAXs could be due to their inherent porosity, fibril arrangement, and available surface area for hydrophilic interactions. The higher water-holding capacity of FAX treatments from maize bran may be due to its larger pore size, more branched structure, looser fibril arrangement, and higher hydrophilic interactions. Results regarding the oil-holding capacity of all treatments showed 2.96 ± 0.01 to 3.78 ± 0.03 g/g absorbed oil/sample.

The results of the emulsion properties of the FAXs are shown in Table 8. The emulsifying activity indicates the amount of oil a protein can emulsify per unit of water. At the same time, emulsion stability (ES) refers to its ability to produce an emulsion that remains stable against droplet aggregation. The current results showed that FAX exhibited 41 ± 0.04 to 51 ± 0.08% emulsifying activity across different treatments. Emulsion stability ranged from 72.5 ± 2.9 to 89.7 ± 2.4% across all treatments. Previous literature has shown that polysaccharides exhibit strong emulsifying properties and are commonly used in industrial production [55,56]. In the current case, FAX’s polysaccharide emulsifying properties are due to the interaction between polysaccharides and their associated proteins. The proteins are hydrophobic, while the polysaccharide chains are hydrophilic. The FAX moieties in protein–polysaccharide conjugates provide steric and electrostatic repulsions, which stabilize emulsions.

### 3.11. Implications and Limitations

The results of this study demonstrate that maize bran can be effectively valorized as a source of FAXs using mild alkaline hydrolysis, yielding biopolymers with retained phenolic association, antioxidant capacity, and favorable functional properties such as water-holding and emulsifying behavior. These attributes support the potential application of maize bran-derived FAXs as functional ingredients in food, nutraceutical, and related biopolymer systems where dietary fiber functionality and antioxidant activity are desirable.

This study is subject to certain limitations. Structural characterization was limited to compositional analysis and spectroscopic confirmation, and therefore the molecular weight distribution and fine chain-branching architecture were not resolved. In addition, DOE was applied to identify practical extraction conditions rather than to develop a fully predictive kinetic model. Despite these limitations, the integrated assessment of extraction performance, chemical composition, phenolic retention, antioxidant activity, and functional properties provides a robust framework for evaluating the applicability of maize bran FAXs and identifies clear directions for future work, including advanced structural analysis and application-specific performance testing.

## 4. Conclusions

This study demonstrates the effective valorization of maize bran as a source of FAXs through a systematically optimized alkaline hydrolysis process. By applying a design-of-experiments framework under mild, application-relevant conditions, the work establishes robust extraction parameters that maximize FAX recovery while preserving phenolic association and functional integrity. Rather than emphasizing individual numerical outcomes, the study highlights how extraction conditions influence the balance between polysaccharide solubilization, ferulic acid retention, antioxidant activity, and functional performance. However, the phenolic content, antioxidant activity, and functional properties were measured comparatively but were not included in the DOE.

This work provides insight into the chemical composition, phenolic association, and functionality of maize bran FAXs. In particular, the results demonstrate that mild alkaline treatment enhances the accessibility of bound phenolics without substantial disruption of the arabinoxylan backbone, leading to biopolymers with strong antioxidant capacity, high water-holding ability, and effective emulsifying behavior. Qualitative microstructural observations further support the link between alkaline-induced loosening of the polysaccharide network and improved functional properties. Based on the integrated evaluation of extraction yield, phenolic association, antioxidant capacity, and functional properties, alkaline hydrolysis conducted at moderate alkali concentration and extraction time under mild temperature conditions (25–35 °C) produced ferulated arabinoxylans that best balanced recovery efficiency with preservation of phenolic content and functional performance.

Overall, this work advances current knowledge by coupling systematic process optimization with multi-level characterization to define maize bran FAXs as functional, phenolic-rich biopolymers with potential utility in food, nutraceutical, and related applications. While more advanced structural analyses and application-specific performance testing are warranted, the findings provide a practical and scientifically grounded framework for the recovery and utilization of cereal byproduct-derived arabinoxylans. Importantly, these results demonstrate that optimal FAX recovery does not require elevated temperatures, supporting the feasibility of robust, energy-efficient extraction under mild processing conditions.

## Figures and Tables

**Table 1:** Extraction of ferulated arabinoxylans from maize bran at different KOH concentrations, times, and temperatures.

Sample Run	Factors			Purified Yield of FAXs (%)	Coding of Selected Samples
	KOH Conc. (%)	Time (Hours)	Temperature (°C)		
1	5	4	25	14.50 ± 0.04 g	FAX 1
2	5	4	30	14.50 ± 0.04 g	-
3	5	4	35	14.55 ± 0.05 g	-
4	5	5	25	18.90 ± 0.05 a	FAX 2
5	5	5	30	18.90 ± 0.04 a	-
6	5	5	35	18.90 ± 0.04 a	-
7	5	6	25	16.20 ± 0.04 c	FAX 3
8	5	6	30	16.20 ± 0.04 c	-
9	5	6	35	16.20 ± 0.04 c	-
10	6	4	25	15.20 ± 0.04 e	FAX 4
11	6	4	30	15.20 ± 0.04 e	-
12	6	4	35	15.20 ± 0.04 e	-
13	6	5	25	16.10 ± 0.02 d	FAX 5
14	6	5	30	16.10 ± 0.03 d	-
15	6	5	35	16.10 ± 0.04 d	-
16	6	6	25	14.70 ± 0.02 f	FAX 6
17	6	6	30	14.70 ± 0.04 f	-
18	6	6	35	14.70 ± 0.04 f	-
19	7	4	25	15.80 ± 0.05 e	FAX 7
20	7	4	30	15.80 ± 0.04 e	-
21	7	4	35	15.80 ± 0.04 e	-
22	7	5	25	16.30 ± 0.04 b	FAX 8
23	7	5	30	16.30 ± 0.04 b	-
24	7	5	35	16.30 ± 0.04 b	-
25	7	6	25	16.10 ± 0.04 d	FAX 9
26	7	6	30	16.10 ± 0.04 d	-
27	7	6	35	16.10 ± 0.04 d	-

FAX 1: KOH concentration 5%, time 4 h, temperature 25, 30 and 35 °C, FAX 2: KOH concentration 5%, time 5 h, temperature 25, 30 and 35 °C, FAX 3: KOH concentration 5%, time 6 h, temperature 25, 30 and 35 °C, FAX 4: KOH concentration 6%, time 4 h, temperature 25, 30 and 35 °C, FAX 5: KOH concentration 6%, time 5 h, temperature 25, 30 and 35 °C, FAX 6: KOH concentration 6%, time 6 h, temperature 25, 30 and 35 °C, FAX 7: KOH concentration 7%, time 4 h, temperature 25, 30 and 35 °C, FAX 8: KOH concentration 7%, time 5 h, temperature 25, 30 and 35 °C, FAX 9: KOH concentration 7%, time 6 h, temperature 25, 30 and 35 °C, Different letters (a–g) within the column indicated the interactions of treatments are significantly different (*p* ≤ 0.05). Values with the same lettering showed no significant differences. Results are expressed as the mean value ± standard deviation (n = 2).

**Table 2:** Estimates of regression coefficients for the factorial DOE model explaining FAX yields.

Term	Coefficient β	Standard Error Coefficient	95% Confidence Interval	t-Value	*p*-Value	Variance Inflation Factor (VIF)
Constant	16.048	0.606	(14.747, 17.348)	26.47	0.000	-
Treatments	−0.0620	0.0408	(−0.1496, 0.0256)	−1.52	0.151	1.69
KOH Concentration % X_1_						
5	−0.062	0.377	(−0.870, 0.746)	−0.16	0.871	1.57
6	−0.000	0.374	(−0.803, 0.803)	−0.00	1.000	1.56
Time (hours) X_2_						
4	−2.68	1.23	(−5.32, −0.03)	−2.17	0.048	15.64
5	0.300	0.707	(−1.217, 1.817)	0.42	0.678	6.17
Temperature (°C) X_3_						
25	3.37	1.70	(−0.27, 7.02)	1.98	0.067	10.82
30	1.254	0.928	(−0.736, 3.245)	1.35	0.198	4.25
35	−1.524	0.960	(−3.582, 0.535)	−1.59	0.135	4.55
KOH Concentration % Time (hours) X_1_X_2_						
5 4	−0.000	0.566	(−1.214, 1.214)	−0.00	1.000	2.34
5 5	0.000	0.510	(−1.094, 1.094)	0.00	1.000	2.08
6 4	−0.000	0.566	(−1.214, 1.214)	−0.00	1.000	2.17
6 5	0.000	0.510	(−1.094, 1.094)	0.00	1.000	2.22

**Table 3:** Monosaccharide composition and protein content of ferulated arabinoxylans extracted from maize bran.

Chemical Components	FAX 1	FAX 2	FAX 3	FAX 4	FAX 5	FAX 6	FAX 7	FAX 8	FAX 9
Arabinose (%)	16.0 ± 1.29 ^e^	12.7 ± 1.74 ^f^	17.2 ± 4.12 ^e^	30.4 ± 0.86 ^a^	25.5 ± 6.10 ^c^	15.7 ± 1.43 ^e^	27.4 ± 4.54 ^b^	23.9 ± 2.20 ^d^	26.2 ± 1.33 ^b^
Xylose (%)	22.4 ± 2.82 ^e^	18.3 ± 2.63 ^f^	23.3 ± 6.52 ^e^	44.7 ± 2.49 ^a^	36.0 ± 9.85 ^c^	21.8 ± 1.92 ^e^	38.1 ± 6.28 ^b^	32.1 ± 2.82 ^d^	33.5 ± 1.26 ^d^
Galactose (%)	3.1 ± 0.09 ^e^	2.7 ± 0.40 ^f^	3.9 ± 0.66 ^d^	7.4 ± 0.39 ^a^	5.8 ± 1.52 ^c^	3.5 ± 0.20 ^d^	6.6 ± 1.26 ^b^	5.6 ± 0.53 ^c^	6.1 ± 0.30 ^c^
Glucose (%)	6.1 ± 0.37 ^c^	4.8 ± 0.54 ^e^	7.3 ± 0.85 ^b^	9.2 ± 0.39 ^a^	7.3 ± 1.61 ^b^	4.6 ± 0.36 ^e^	6.5 ± 1.17 ^c^	5.9 ± 0.59 ^d^	6.0 ± 0.23 ^c^
Ara/Xyl Ratio	0.71 ± 0.02 ^d^	0.69 ± 0.01 ^e^	0.73 ± 0.03 ^b^	0.68 ± 0.01 ^f^	0.68 ± 0.03 ^f^	0.72 ± 0.03 ^c^	0.71 ± 0.02 ^d^	0.74 ± 0.03 ^a^	0.73 ± 0.02 ^b^
Protein (%)	3.58 ± 0.05 ^a^	2.86 ± 0.06 ^c^	3.37 ± 0.05 ^b^	0.96 ± 0.04 ^g^	1.23 ± 0.09 ^f^	1.51 ± 0.02 ^e^	2.09 ± 0.04 ^d^	2.01 ± 0.08 ^d^	1.8 ± 0.06 ^e^

FAX 1: KOH concentration 5%, time 4 h, temperature 25, 30 and 35 °C, FAX 2: KOH concentration 5%, time 5 h, temperature 25, 30 and 35 °C, FAX 3: KOH concentration 5%, time 6 h, temperature 25, 30 and 35 °C, FAX 4: KOH concentration 6%, time 4 h, temperature 25, 30 and 35 °C, FAX 5: KOH concentration 6%, time 5 h, temperature 25, 30 and 35 °C, FAX 6: KOH concentration 6%, time 6 h, temperature 25, 30 and 35 °C, FAX 7: KOH concentration 7%, time 4 h, temperature 25, 30 and 35 °C, FAX 8: KOH concentration 7%, time 5 h, temperature 25, 30 and 35 °C, FAX 9: KOH concentration 7%, time 6 h, temperature 25, 30 and 35 °C. Different letters (a–g) within the row indicate that the interaction of treatments is significantly different (*p* ≤ 0.05). Values with the same lettering showed no significant differences. Results are expressed as the mean value ± standard deviation (n = 3).

**Table 4:** Total phenolic content of different treatments of ferulated arabinoxylans extracted from maize bran.

Total Phenolic Content	FAX 1	FAX 2	FAX 3	FAX 4	FAX 5	FAX 6	FAX 7	FAX 8	FAX 9
Free-form (μg/g FAE)	75.7 ± 0.83 ^bE^	39.4 ± 0.08 ^dD^	114.4 ± 1.76 ^aD^	23.5 ± 0.44 ^eE^	17.7 ± 1.10 ^fE^	48.1 ± 0.08 ^cE^	9.0 ± 0.13 ^gE^	18.3 ± 0.13 ^fE^	9.8 ± 0.08 ^gE^
Alkaline-extracted (μg/g FAE)	909.7 ± 1.32 ^bA^	592.8 ± 0.44 ^dA^	978.8 ± 2.7 ^aA^	155.0 ± 5.3 ^iA^	408.8 ± 4.4 ^gA^	634.4 ± 3.5 ^cA^	566.9 ± 5.3 ^eA^	536.3 ± 0.9 ^fA^	303.4 ± 2.2 ^hB^
Acid-extracted (μg/g FAE)	291.1 ± 1.0 ^cB^	264.3 ± 0.7 ^eB^	347.6 ± 0.22 ^aB^	105.4 ± 0.22 ^iB^	184.1 ± 0.57 ^hC^	281.0 ± 0.75 ^dB^	225.3 ± 0.97 ^fC^	188.2 ± 0.13 ^gC^	308.8 ± 0.04 ^bA^
Fraction I, bound (μg/g FAE)	107.2 ± 0.48 ^aD^	27.1 ± 0.13 ^iE^	90.2 ± 0.39 ^dE^	100.8 ± 0.26 ^bC^	55.0 ± 0.53 ^hD^	88.5 ± 0.44 ^eD^	79.8 ± 0.13 ^fD^	67.5 ± 0.30 ^gD^	99.4 ± 0.44 ^cC^
Fraction II, bound (μg/g FAE)	253.8 ± 10.6 ^bC^	255.9 ± 10.1 ^bC^	196.9 ± 1.76 ^eC^	58.1 ± 1.76 ^gD^	223.8 ± 4.41 ^dB^	186.3 ± 5.30 ^fC^	292.8 ± 3.09 ^aB^	230.9 ± 3.09 ^cB^	48.8 ± 0.00 ^hD^

FAX 1: KOH concentration 5%, time 4 h, temperature 25, 30 and 35 °C, FAX 2: KOH concentration 5%, time 5 h, temperature 25, 30 and 35 °C, FAX 3: KOH concentration 5%, time 6 h, temperature 25, 30 and 35 °C, FAX 4: KOH concentration 6%, time 4 h, temperature 25, 30 and 35 °C, FAX 5: KOH concentration 6%, time 5 h, temperature 25, 30 and 35 °C, FAX 6: KOH concentration 6%, time 6 h, temperature 25, 30 and 35 °C, FAX 7: KOH concentration 7%, time 4 h, temperature 25, 30 and 35 °C, FAX 8: KOH concentration 7%, Time 5 h, temperature 25, 30 and 35 °C, FAX 9: KOH concentration 7%, time 6 h, temperature 25, 30 and 35 °C; TPC: Total phenolic content; different letters (a–i) within the row indicate the interaction of treatments are significantly different (*p* ≤ 0.05). Different letters (A–E) within the column suggest that the interaction of the extracts is significantly different (*p* ≤ 0.05). Results are expressed as the mean value ± standard deviation (n = 2).

**Table 5:** Quantification of ferulic acid contents through HPLC in different treatments of ferulated arabinoxylans extracted from maize bran.

Ferulic Acid	FAX 1	FAX 2	FAX 3	FAX 4	FAX 5	FAX 6	FAX 7	FAX 8	FAX 9
FFFA (μg/g)	16.33	5.05	44.71	3.56	0.34	4.25	0.44	0.53	2.73
Alkaline-extracted FA (μg/g)	252.21	165.56	267.88	63.49	140.59	262.5	204.36	181.56	115.93
Acid-extracted FA (μg/g)	8.68	7.18	11.62	1.78	2.45	2.85	6.43	4.62	7.49
Fraction I BFFA (μg/g)	3.35	1.25	2.09	2.96	2.78	1.58	2.31	2.01	4.11
Fraction II BFFA (μg/g)	75.88	78.77	70.29	28.56	92.3	66.48	127.32	83.85	48.54

FAX 1: KOH concentration 5%, time 4 h, temperature 25, 30 and 35 °C, FAX 2: KOH concentration 5%, time 5 h, temperature 25, 30 and 35 °C, FAX 3: KOH concentration 5%, time 6 h, temperature 25, 30 and 35 °C, FAX 4: KOH concentration 6%, time 4 h, temperature 25, 30 and 35 °C, FAX 5: KOH concentration 6%, time 5 h, temperature 25, 30 and 35 °C, FAX 6: KOH concentration 6%, time 6 h, temperature 25, 30 and 35 °C, FAX 7: KOH concentration 7%, time 4 h, temperature 25, 30 and 35 °C, FAX 8: KOH concentration 7%, time 5 h, temperature 25, 30 and 35 °C, FAX 9: KOH concentration 7%, time 6 h, temperature 25, 30 and 35 °C; FFFA: free-form ferulic acid, FA: ferulic acid, BFFA: bound-form ferulic acid, HPLC: high-performance liquid chromatography.

**Table 6:** Antioxidant activity of ferulated arabinoxylan extracts.

Treatments	FAX1	FAX2	FAX3	FAX4	FAX5	FAX6	FAX7	FAX8	FAX9	
Ferric Reducing Antioxidant Power (FRAP)										
Free form of bioactive FAX extract μg/g TE	56.5 ± 0.19 ^bE^	49.5 ± 0.12 ^cD^	122.6 ± 0.24 ^aD^	29.4 ± 0.19 ^dE^	11.6 ± 0.05 ^iE^	19.5 ± 0.01 ^fE^	16.5 ± 0.19 ^gE^	25.7 ± 0.01 ^eE^	13.2 ± 0.01 ^hE^	
Alkaline-extracted (Bound-form extract) μg/g TE	894.7 ± 1.99 ^bA^	616.1 ± 0.34 ^eA^	905.0 ± 3.55 ^aA^	214.5 ± 0.04 ^iA^	384.2 ± 0.87 ^gA^	727.5 ± 1.55 ^cA^	624.3 ± 0.92 ^dA^	594.7 ± 1.60 ^fA^	374.7 ± 0.48 ^hA^	
Acid-extracted (Bound-form extract) μg/g TE	246.7 ± 3.39 ^cC^	223.6 ± 2.36 ^dC^	260.0 ± 0.75 ^aC^	119.8 ± 0.83 ^hB^	177.1 ± 0.54 ^gC^	245.2 ± 0.47 ^cB^	217.8 ± 0.40 ^eC^	205.9 ± 2.28 ^fC^	256.1 ± 1.41 ^bB^	
Fraction I (Bound-form extract) μg/g TE	108.6 ± 0.20 ^aD^	25.3 ± 0.06 ^hE^	94.3 ± 0.12 ^bE^	81.3 ± 0.20 ^dD^	46.7 ± 0.01 ^gD^	83.6 ± 0.36 ^cD^	63.6 ± 0.13 ^eD^	48.0 ± 0 ^fD^	80.5 ± 0.20 ^eD^	
Fraction II (Bound-form extract) μg/g TE	298.6 ± 1.07 ^dB^	310.5 ± 1.07 ^cB^	286.5 ± 0.43 ^eB^	118.7 ± 0.43 ^iC^	283.7 ± 0.53 ^fB^	242.3 ± 1.16 ^gC^	367.9 ± 1.36 ^aB^	318.8 ± 0.48 ^bB^	188.3 ± 0.24 ^hC^	
2,2′-azinobis-(3-ethylbenzothiazoline-6-sulfonic acid) (ABTS)										
Free form of bioactive FAX extract μg/g TE	75.1 ± 0.01 ^bC^	74.7 ± 0.03 ^bC^	428.7 ± 4.19 ^aB^	74.0 ± 0.11 ^bB^	29.3 ± 0.31 ^eD^	73.7 ± 0.12 ^bC^	34.0 ± 0.25 ^dE^	40.5 ± 0.30 ^cD^	74.2 ± 0.16 ^bC^	
Alkaline-extracted (Bound-form extract) μg/g TE	596.0 ± 10.6 ^CA^	376.7 ± 2.53 ^iA^	578.1 ± 2.37 ^dA^	413.6 ± 1.97 ^hA^	627.5 ± 4.19 ^bA^	705.7 ± 2.05 ^aA^	549.3 ± 2.05 ^eA^	534.2 ± 3.87 ^fA^	491.5 ± 2.45 ^gA^	
Acid-extracted (Bound-form extract) μg/g TE	71.7 ± 0.18 ^bD^	68.0 ± 0.29 ^dD^	74.5 ± 0.03 ^aD^	51.5 ± 0.37 ^iD^	60.8 ± 0.27 ^hC^	67.6 ± 0.23 ^eD^	65.7 ± 0.30 ^fC^	61.8 ± 0.34 ^gC^	70.3 ± 0.19 ^cD^	
Fraction I (Bound-form extract) μg/g TE	39.3 ± 1.03 ^dE^	11.0 ± 0.63 ^iE^	35.1 ± 0.55 ^fE^	37.0 ± 0.45 ^eE^	14.8 ± 0.25 ^hE^	46.9 ± 0.42 ^aE^	45.5 ± 0.33 ^bD^	32.0 ± 0.30 ^gE^	40.7 ± 0.49 ^cE^	
Fraction II (Bound-form extract) μg/g TE	250.2 ± 3.00 ^cB^	264.2 ± 3.24 ^bB^	266.6 ± 2.45 ^aC^	67.9 ± 2.21 ^iC^	200.7 ± 3.16 ^fB^	146.4 ± 3.32 ^gB^	220.3 ± 3.32 ^eB^	221.6 ± 4.03 ^dB^	136.2 ± 4.11 ^hB^	
2,2-diphenyl-1-picrylhydrazyl (DPPH)										
Free form of bioactive FAX extract μg/g TE	20.6 ± 0.23 ^cE^	21.1 ± 0.58 ^bE^	64.4 ± 3.74 ^aE^	13.4 ±0.49 ^dE^	13.7 ± 0.03 ^dE^	4.8 ± 0.35 ^gE^	12.3 ± 0.04 ^eE^	9.3 ± 0.02 ^fE^	2.9 ± 0.21 ^hE^	
Alkaline-extracted (Bound-form extract) μg/g TE	511.5 ± 1.14 ^bA^	292.6 ± 1.66 ^fA^	560.1 ± 2.59 ^aA^	140.1 ± 2.07 ^iB^	251.7 ± 0.72 ^gA^	435.9 ± 2.28 ^cA^	397.8 ± 2.28 ^dA^	373.0 ± 1.14 ^eA^	236.1 ± 0.31 ^hA^	
Acid-extracted (Bound-form extract) μg/g TE	73.6 ± 0.29 ^bD^	67.6 ± 0.34 ^dD^	80.7 ± 0.18 ^aC^	43.3 ± 0.17 ^hD^	55.5 ± 0.31 ^gD^	68.2 ± 0.41 ^dC^	60.1 ± 0.31 ^fC^	60.8 ± 0.38 ^eC^	71.5 ± 0.36 ^cC^	
Fraction I (Bound-form extract) μg/g TE	78.6 ± 0.27 ^bC^	74.5 ± 0.32 ^cC^	79.6 ± 0.23 ^aD^	69.3 ± 0.32 ^dC^	59.5 ± 0.39 ^eC^	34.8 ± 0.20 ^fD^	34.2 ± 0.13 ^fD^	32.6 ± 0.21 ^gD^	43.1 ± 0.31 ^eD^	
Fraction II (Bound-form extract) μg/g TE	200.8 ± 2.59 ^eB^	212.4 ± 2.59 ^dB^	197.2 ± 2.91 ^eB^	180.7 ± 1.66 ^gA^	246.0 ± 3.63 ^bB^	289.0 ± 3.95 ^aB^	241.5 ± 2.80 ^cB^	223.1 ± 4.78 ^cB^	196.8 ± 2.28 ^fB^	

FAX1: KOH concentration 5%, time 4 h, temperature 25, 30 and 35 °C, FAX2: KOH concentration 5%, time 5 h, temperature 25, 30 and 35 °C, FAX3: KOH concentration 5%, time 6 h, temperature 25, 30 and 35 °C, FAX4: KOH concentration 6%, time 4 h, temperature 25, 30 and 35 °C, FAX5: KOH concentration 6%, time 5 h, temperature 25, 30 and 35 °C, FAX6: KOH concentration 6%, time 6 h, temperature 25, 30 and 35 °C, FAX7: KOH concentration 7%, time 4 h, temperature 25, 30 and 35 °C, FAX8: KOH concentration 7%, time 5 h, temperature 25, 30 and 35 °C, FAX9: KOH concentration 7%, time 6 h, temperature 25, 30 and 35 °C; Antioxidant activity; different letters (a–i) within the row indicate the interaction of treatments are significantly different (*p* ≤ 0.05). Different letters (A–E) within the column suggest that the interaction of the extracts is significantly different (*p* ≤ 0.05). Values with the same lettering showed no significant differences. Results are expressed as the mean value ± standard deviation (n = 2). TE = Trolox equivalents. Values are expressed as μg Trolox equivalent per gram of extract (μg/g TE).

**Table 7:** Wave numbers and their associated functional groups on FT-IR spectra of different treatments of ferulated arabinoxylans extracted from maize bran.

Wave Number (cm^−1^)	Functional Groups	Current Results	Reference
520–760	Polymer backbone	Peaks confirmed	[27,42]
900–1300	Polysaccharides and nucleic acids (C-O-H bending)	Peaks confirmed	[43,44]
1500–1700	Proteins (amide I and amide II)	Peaks confirmed	[45,46]
2100–2200	Alkyne or nitrile bonds in polymer structure (triple bond of C≡C and N=N=N in azides)	Peaks confirmed	[47,48]
2880, 2900–3100	CH_2_ groups in glycerol; C–H stretching bond (aliphatic H)	Peaks confirmed	[49,50]
3100–3300	Broad absorbance bands for –OH-group stretching	Peaks confirmed	[51,52]

**Table 8:** Functional characteristics of ferulated arabinoxylans extracted from maize bran.

Treatments	Water-Holding Capacity (g/g)	Oil-Holding Capacity (g/g)	Emulsion Activity (%)	Emulsion Stability (%)
FAX 1	9.2 ± 0.05 ^a^	2.96 ± 0.01 ^f^	51 ± 0.08 ^a^	89.7 ± 2.4 ^a^
FAX 2	8.2 ± 0.04 ^b^	3.62 ± 0.02 ^b^	45 ± 0.06 ^b^	82.6 ± 2.3 ^b^
FAX 3	8.9 ± 0.06 ^a^	3.42 ± 0.02 ^c^	49 ± 0.07 ^a^	88.7 ± 2.9 ^a^
FAX 4	9.7 ± 0.03 ^a^	3.28 ± 0.03 ^d^	41 ± 0.04 ^c^	72.5 ± 2.9 ^d^
FAX 5	8.6 ± 0.05 ^b^	3.64 ± 0.05 ^b^	43 ± 0.05 ^c^	77.2 ± 2.2 ^c^
FAX 6	8.5 ± 0.04 ^b^	3.78 ± 0.03 ^a^	45 ± 0.09 ^b^	79.6 ± 2.1 ^c^
FAX 7	8.3 ± 0.06 ^b^	3.44 ± 0.04 ^c^	46 ± 0.04 ^b^	82.5 ± 1.8 ^b^
FAX 8	8.8 ± 0.07 ^a^	3.54 ± 0.05 ^c^	45 + 0.08 ^b^	80.2 ± 2.4 ^b^
FAX 9	9.4 ± 0.03 ^a^	3.12 ± 0.03 ^e^	42 ± 0.07 ^c^	81.6 ± 3.2 ^b^

FAX 1: KOH concentration 5%, time 4 h, temperature 25, 30 and 35 °C, FAX 2: KOH concentration 5%, time 5 h, temperature 25, 30 and 35 °C, FAX 3: KOH concentration 5%, time 6 h, temperature 25, 30 and 35 °C, FAX 4: KOH concentration 6%, time 4 h, temperature 25, 30 and 35 °C, FAX 5: KOH concentration 6%, time 5 h, temperature 25, 30 and 35 °C, FAX 6: KOH concentration 6%, time 6 h, temperature 25, 30 and 35 °C, FAX 7: KOH concentration 7%, time 4 h, temperature 25, 30 and 35 °C, FAX 8: KOH concentration 7%, time 5 h, temperature 25, 30 and 35 °C, FAX 9: KOH concentration 7%, time 6 h, temperature 25, 30 and 35 °C. Different letters (a–f) within the column indicate the interaction of the treatments is significantly different (*p* ≤ 0.05). Values with the same lettering show non-significant differences from each other. Results are expressed as the mean value ± standard deviation (n = 3).

## Data Availability

The data presented in this study are available on request from the corresponding author due to privacy.

## Supplementary Materials

The following supporting information can be downloaded at https://www.mdpi.com/article/10.3390/polym18060689/s1, File S1: Supplementary Tables; File S2: (FAX 1 to FAX 9): FTIR spectra of FAX samples; File S3: (FAX1 to FAX9): High performance liquid chromatography chromatograms of ferulic acid analysis.

## References

[1] Mujtaba M., Fraceto L.F., Fazeli M., Mukherjee S., Savassa S.M., de Medeiros G.A., Santo Pereira A.D.E., Mancini S.D., Lipponen J., Vilaplana F. (2023). Lignocellulosic biomass from agricultural waste to the circular economy: A review with focus on biofuels, biocomposites and bioplastics. J. Clean. Prod..

[2] Akin M., Jukic M., Lukinac J., Yilmaz B., Özogul F., Rocha J.M., Spizzirri U.G. (2023). Valorization and Functionalization of Cereal-Based Industry Byproducts for Nutraceuticals. Nutraceutics from Agri-Food By-Products.

[3] Skendi A., Zinoviadou K.G., Papageorgiou M., Rocha J.M. (2020). Advances on the valorisation and functionalization of byproducts and wastes from cereal-based processing industry. Foods.

[4] Lugo-Arias J., Vargas S.B., Maturana A., González-Álvarez J., Lugo-Arias E., Rico H. (2024). Nutrient Removal from Aqueous Solutions Using Biosorbents Derived from Rice and Corn Husk Residues: A Systematic Review from the Environmental Management Perspective. Water.

[5] Saeed F., Hussain M., Arshad M.S., Afzaal M., Munir H., Imran M., Tufail T., Anjum F.M. (2021). Functional and nutraceutical properties of maize bran cell wall non-starch polysaccharides. Int. J. Food Prop..

[6] Liu S., Ding W., Yang Q., Rose D.J. (2024). Alkali treatment of maize bran affects utilization of arabinoxylan and other non-digestible carbohydrates by the human gut microbiota in vitro in a dose-dependent manner. Food Hydrocoll..

[7] Mule T.A., Sawant S.S., Odaneth A.A. (2024). Maize bran as a potential substrate for production of β-glucosidase. Biomass Convers. Biorefinery.

[8] Raza M.A., Saeed F., Afzaal M., Imran A., Niaz B., Hussain M., Rasheed A., Kashif Mukhtar M., Waleed M., Al Jbawi E. (2022). Comparative study of cross-and uncross-linked arabinoxylans extracted from maize bran with special reference to their structural and antioxidant potential. Int. J. Food Prop..

[9] Zannini E., Bravo Núñez Á., Sahin A.W., Arendt E.K. (2022). Arabinoxylans as functional food ingredients: A review. Foods.

[10] Weng V., Cardeira M., Bento-Silva A., Serra A.T., Brazinha C., Bronze M.R. (2023). Arabinoxylan from corn fiber obtained through alkaline extraction and membrane purification: Relating bioactivities with the phenolic compounds. Molecules.

[11] Liu Y., Guo C., Wang C. (2024). Biochemical characterization of an organic solvent-and salt-tolerant xylanase and its application of arabinoxylan-oligosaccharides production from corn fiber gum. Int. J. Biol. Macromol..

[12] Chateigner-Boutin A.L., Saulnier L. (2022). Ferulic and coumaric acids in the cereal grain: Occurrence, biosynthesis, biological and technological functions. Advances in Botanical Research.

[13] Zhang Z., Yang P., Zhao J. (2022). Ferulic acid mediates prebiotic responses of cereal-derived arabinoxylans on host health. Anim. Nutr..

[14] Valério R., Crespo J.G., Galinha C.F., Brazinha C. (2021). Effect of ultrafiltration operating conditions for separation of ferulic acid from arabinoxylans in corn fibre alkaline extract. Sustainability.

[15] Marquez-Escalante J.A., Carvajal-Millan E., Martínez-López A.L., Martínez-Robinson K.G., Campa-Mada A.C., Rascon-Chu A. (2023). Fine structural features and antioxidant capacity of ferulated arabinoxylans extracted from nixtamalized maize bran. J. Sci. Food Agric..

[16] AlYammahi J., Rambabu K., Thanigaivelan A., Bharath G., Hasan S.W., Show P.L., Banat F. (2023). Advances of non-conventional green technologies for phyto-saccharides extraction: Current status and future perspectives. Phytochem. Rev..

[17] Jiang Y., Bai X., Lang S., Zhao Y., Liu C., Yu L. (2019). Optimization of ultrasonic-microwave assisted alkali extraction of arabinoxylan from the corn bran using response surface methodology. Int. J. Biol. Macromol..

[18] Munk L., Muschiol J., Li K., Liu M., Perzon A., Meier S., Ulvskov P., Meyer A.S. (2020). Selective enzymatic release and gel formation by crosslinking of feruloylated glucurono-arabinoxylan from corn bran. ACS Sustain. Chem. Eng..

[19] Wang Y.L., Wang W.K., Wu Q.C., Yang H.J. (2022). The release and catabolism of ferulic acid in plant cell wall by rumen microbes: A review. Anim. Nutr..

[20] Herrera-Balandrano D.D., Báez-González J.G., Carvajal-Millán E., Muy-Rangel D., Urías-Orona V., Martínez-López A.L., Márquez-Escalante J.A., Heredia J.B., Niño-Medina G. (2020). Alkali-extracted feruloylated arabinoxylans from nixtamalized maizebran byproduct: A synonymous with soluble antioxidant dietary fiber. Waste Biomass Valorization.

[21] AACC (2000). Approved Methods of American Association of Cereal Chemists.

[22] Pettolino F.A., Walsh C., Fincher G.B., Bacic A. (2012). Determining the polysaccharide composition of plant cell walls. Nat. Protoc..

[23] Kulathunga J., Simsek S. (2024). Stone milling conditions and starter culture source influence phytic acid content and antioxidant activity in whole-grain sourdough bread. Cereal Chem..

[24] Barberousse H., Kamoun A., Chaabouni M., Giet J.M., Roiseux O., Paquot M., Deroanne C., Blecker C. (2009). Optimization of enzymatic extraction of ferulic acid from wheat bran, using response surface methodology, and characterization of the resulting fractions. J. Sci. Food Agric..

[25] Brand-Williams W., Cuvelier M.E., Berset C. (1995). Use of a free radical method to evaluate antioxidant activity. LWT-Food Sci. Technol..

[26] AACC International (2012). Approved Methods of the American Association of Cereal Chemists International.

[27] Kaur A., Singh B., Yadav M.P., Bhinder S., Singh N. (2021). Isolation of arabinoxylan and cellulose-rich arabinoxylan from wheat bran of different varieties and their functionalities. Food Hydrocoll..

[28] Robertson J.A., de Monredon F.D., Dysseler P., Guillon F., Amado R., Thibault J.F. (2000). Hydration properties of dietary fibre and resistant starch: A European collaborative study. LWT-Food Sci. Technol..

[29] Gannasin S.P., Ramakrishnan Y., Adzahan N.M., Muhammad K. (2012). Functional and preliminary characterisation of hydrocolloid from tamarillo (*Solanum betaceum* Cav.) puree. Molecules.

[30] Martínez-López A.L., Carvajal-Millan E., Lizardi-Mendoza J., Rascón-Chu A., López-Franco Y.L., Salas-Muñoz E., Ramírez-Wong B. (2012). Ferulated arabinoxylans as byproduct from maize wet-milling process: Characterization and gelling capability. Maize Cultiv. Uses Health Benefits.

[31] Chanliaud E., Saulnier L., Thibault J.F. (1995). Alkaline extraction and characterisation of heteroxylans from maize bran. J. Cereal Sci..

[32] Mendez-Encinas M.A., Carvajal-Millan E., Rascon-Chu A., Astiazaran-Garcia H.F., Valencia-Rivera D.E. (2018). Ferulated arabinoxylans and their gels: Functional properties and potential application as antioxidant and anticancer agent. Oxidative Med. Cell. Longev..

[33] Méndez D.A., Fabra M.J., Odriozola-Serrano I., Martín-Belloso O., Salvia-Trujillo L., López-Rubio A., Martínez-Abad A. (2022). Influence of the extraction conditions on the carbohydrate and phenolic composition of functional pectin from persimmon waste streams. Food Hydrocoll..

[34] Dai J., Mumper R.J. (2010). Plant phenolics: Extraction, analysis and their antioxidant and anticancer properties. Molecules.

[35] Shi L., Zhao W., Yang Z., Subbiah V., Suleria H.A.R. (2022). Extraction and characterization of phenolic compounds and their potential antioxidant activities. Environ. Sci. Pollut. Res..

[36] Sombutsuwan P., Durand E., Aryusuk K. (2024). Effect of acidity/alkalinity of deep eutectic solvents on the extraction profiles of phenolics and biomolecules in defatted rice bran extract. PeerJ Anal. Chem..

[37] Huang W., Tian F., Wang H., Wu S., Jin W., Shen W., Hu Z., Cai Q., Liu G. (2023). Comparative assessment of extraction, composition, and in vitro antioxidative properties of wheat bran polyphenols. LWT.

[38] Bauer J.L., Harbaum-Piayda B., Schwarz K. (2012). Phenolic compounds from hydrolyzed and extracted fiber-rich byproducts. LWT.

[39] Rocchetti G., Gregorio R.P., Lorenzo J.M., Barba F.J., Oliveira P.G., Prieto M.A., Simal-Gandara J., Mosele J.I., Motilva M.J., Tomas M. (2022). Functional implications of bound phenolic compounds and phenolics–food interaction: A review. Compr. Rev. Food Sci. Food Saf..

[40] Ji Y., Yang X., Ji Z., Zhu L., Ma N., Chen D., Jia X., Tang J., Cao Y. (2020). DFT-calculated IR spectrum amide I, II, and III band contributions of N-methylacetamide fine components. ACS Omega.

[41] Hussain M., Saeed F., Niaz B., Imran A., Tufail T. (2022). Biochemical and structural characterization of ferulated arabinoxylans extracted from nixtamalized and non-nixtamalized maize bran. Foods.

[42] Sohni S., Begum S., Hashim R., Khan S.B., Mazhar F., Syed F., Khan S.A. (2024). Physicochemical characterization of microcrystalline cellulose derived from underutilized orange peel waste as a sustainable resource under biorefinery concept. Bioresour. Technol. Rep..

[43] Rehman Z.U., Vrouwenvelder J.S., Saikaly P.E. (2021). Physicochemical properties of extracellular polymeric substances produced by three bacterial isolates from biofouled reverse osmosis membranes. Front. Microbiol..

[44] Brian-Jaisson F., Molmeret M., Fahs A., Guentas-Dombrowsky L., Culioli G., Blache Y., Cérantola S., Ortalo-Magné A. (2016). Characterization and anti-biofilm activity of extracellular polymeric substances produced by the marine biofilm-forming bacterium Pseudoalteromonas ulvae strain TC14. Biofouling.

[45] Jiao Y., Cody G.D., Harding A.K., Wilmes P., Schrenk M., Wheeler K.E., Banfield J.F., Thelen M.P. (2010). Characterization of extracellular polymeric substances from acidophilic microbial biofilms. Appl. Environ. Microbiol..

[46] Tian K., Shao Z., Chen X. (2012). Investigation on thermally-induced conformation transition of soy protein film with variable-temperature FTIR spectroscopy. J. Appl. Polym. Sci..

[47] Nandiyanto A.B.D., Ragadhita R., Fiandini M. (2023). Interpretation of Fourier transform infrared spectra (FTIR): A practical approach in the polymer/plastic thermal decomposition. Indones. J. Sci. Technol..

[48] Shurvell H.F. (2006). Spectra–structure correlations in the mid-and far-infrared. Handb. Vib. Spectrosc..

[49] Peng F., Ren J.L., Xu F., Bian J., Peng P., Sun R.C. (2009). Comparative study of hemicelluloses obtained by graded ethanol precipitation from sugarcane bagasse. J. Agric. Food Chem..

[50] Coelho R.R., Hovell I., Moreno E.L., de Souza A.L., Rajagopal K. (2007). Characterization of functional groups of asphaltenes in vacuum residues using molecular modelling and FTIR techniques. Pet. Sci. Technol..

[51] Bokovets S.P., Pertsevoi F.V., Murlykina N.V., Smetanska I.M., Borankulova A.S., Ianchyk M.V., Omelchenko S.B., Grinchenko O.O., Grychenko N.G., Dikhtyar A.M. (2023). Investigation of infrared spectra of agar-based gel systems for the production of jelly bars. J. Chem. Technol..

[52] Ludwig B. (2022). Infrared spectroscopy studies of aluminum oxide and metallic aluminum powders, part II: Adsorption reactions of organofunctional silanes. Powders.

[53] Chen S., Qin L., Xie L., Yu Q., Chen Y., Chen T., Lu H., Xie J. (2022). Physicochemical characterization, rheological and antioxidant properties of three alkali-extracted polysaccharides from mung bean skin. Food Hydrocoll..

[54] Schendel R.R., Meyer M.R., Bunzel M. (2016). Quantitative profiling of feruloylated arabinoxylan side-chains from graminaceous cell walls. Front. Plant Sci..

[55] Tang Q., Huang G. (2022). Improving method, properties and application of polysaccharide as emulsifier. Food Chem..

[56] Bai L., Huan S., Li Z., McClements D.J. (2017). Comparison of emulsifying properties of food-grade polysaccharides in oil-in-water emulsions: Gum arabic, beet pectin, and corn fiber gum. Food Hydrocoll..

