# Supplementary material for: Optimized Alkaline Hydrolysis for Recovering Ferulated Arabinoxylan Biopolymers from Maize Bran with Antioxidant Functionality
Source: Polymers (Basel). 2026 Mar 12;18(6):689. doi: 10.3390/polym18060689 (PMC13030836; doi:10.3390/polym18060689)
Supplement: Supplementary file 1 [file polymers-18-00689-s001.zip › Supplementary File S1.pdf]

Supplementary Tables

Table S1: Chemical composition of maize bran (% dry weight basis).

Components	Composition (%)
Moisture	10.2±0.04
Crude Protein	8.2±0.03
Ash	1.4±0.01
Crude Fat	4.2±0.02
Crude Fiber	7.5±0.01
Total Carbohydrates	59.4±0.04
Soluble dietary fibers	5.8±0.04
Insoluble dietary fibers	51.9±1.4
Total dietary fibers	57.3±1.5

Values are expressed as mean ± standard deviation (n = 3).

Table S2: Analysis of Variance ANOVA for the factorial design evaluating the effect of extraction parameters on FAX yield.

Source	DF	Seq SS	Contribution	Adj SS	Adj MS	F-Value	P-Value
Model	12	14.6675	39.25%	14.6675	1.22229	0.75	0.685
Covariates	1	0.7224	1.93%	3.7408	3.74083	2.31	0.151
Treatments	1	0.7224	1.93%	3.7408	3.74083	2.31	0.151
Linear	7	13.9451	37.32%	12.5018	1.78597	1.10	0.414
KOH Conc. %	2	0.0669	0.18%	0.0585	0.02923	0.02	0.982
Time (hours)	2	5.1394	13.75%	7.7772	3.88859	2.40	0.127
Temperature (°C)	3	8.7387	23.39%	7.6686	2.55620	1.58	0.239
2-Way Interactions	4	0.0000	0.00%	0.0000	0.00000	0.00	1.000
KOH Conc. %*Time (hours)	4	0.0000	0.00%	0.0000	0.00000	0.00	1.000
Error	14	22.6992	60.75%	22.6992	1.62137		
Total	26	37.3667	100.00%				

DF = degrees of freedom; Seq SS = sequential sum of squares; Adj SS = adjusted sum of squares; Adj MS = adjusted mean square; F-value = test statistic for significance; P-value = probability value; Contribution (%) represents the relative influence of each factor on the response.
