# Supplementary material for: Optimized Alkaline Hydrolysis for Recovering Ferulated Arabinoxylan Biopolymers from Maize Bran with Antioxidant Functionality
Source: Polymers (Basel). 2026 Mar 12;18(6):689. doi: 10.3390/polym18060689 (PMC13030836; doi:10.3390/polym18060689)
Supplement: Supplementary file 1 [file polymers-18-00689-s001.zip › Supplementary File S2.pdf]

Supplementary File 2

Figure S1 (FAX 1 to FAX 9); FTIR spectra of FAX samples

FAX 1

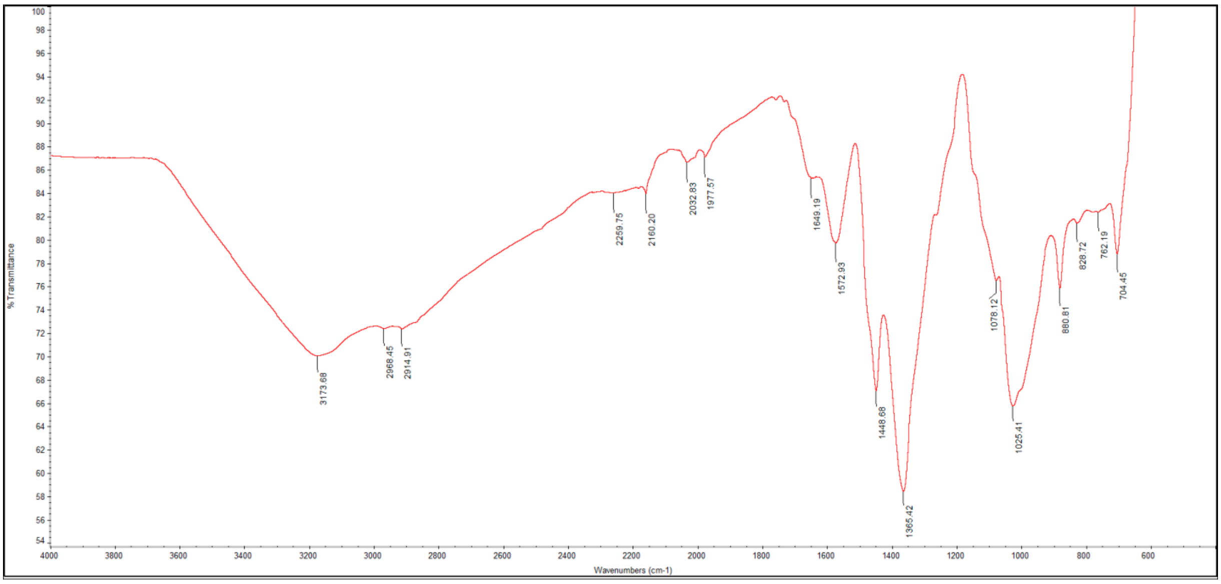

FAX 2

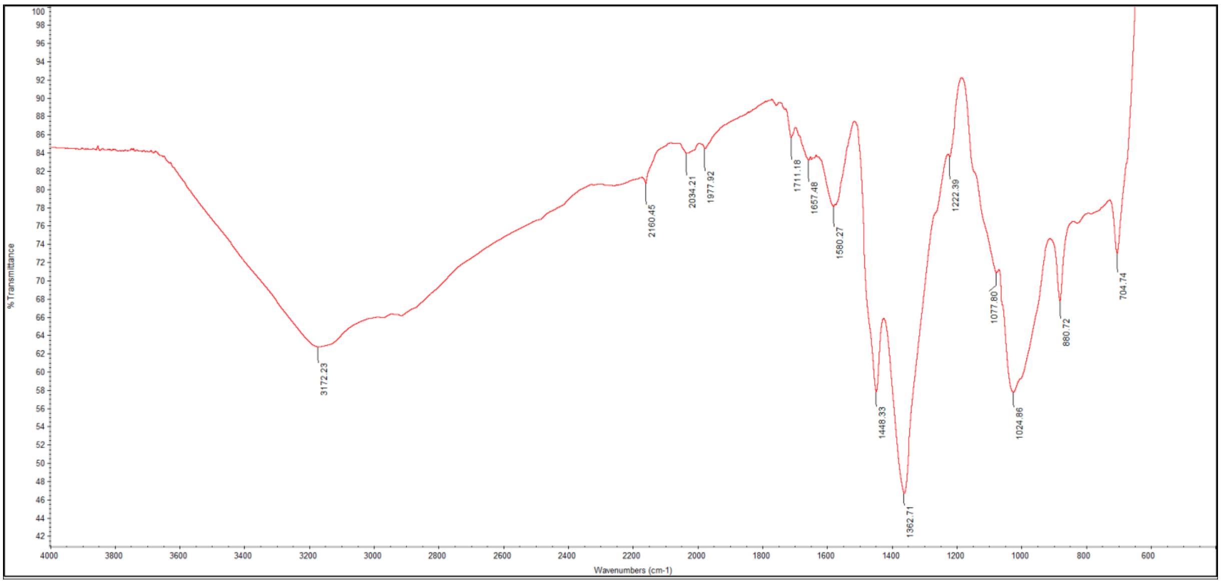

FAX 3

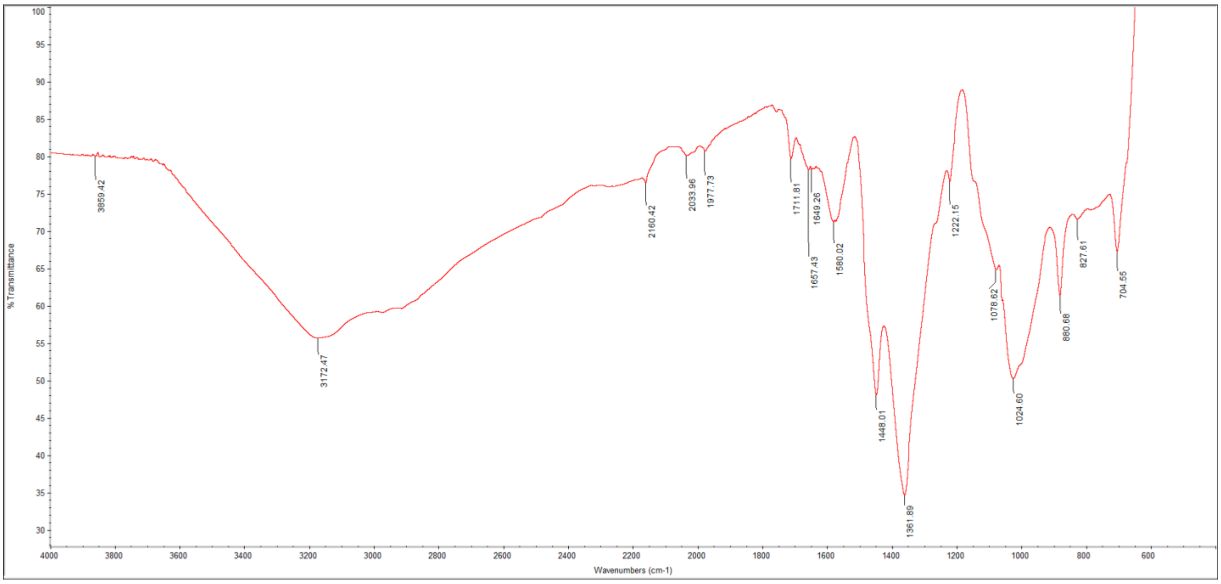
% Transmittance
Wavenumbers (cm-1)
3859.42
3172.47
2160.42
2033.96
1977.73
1711.81
1649.26
1657.43
1580.02
1448.01
1361.89
1222.15
1078.62
1024.60
880.68
827.61
704.55

FAX 4

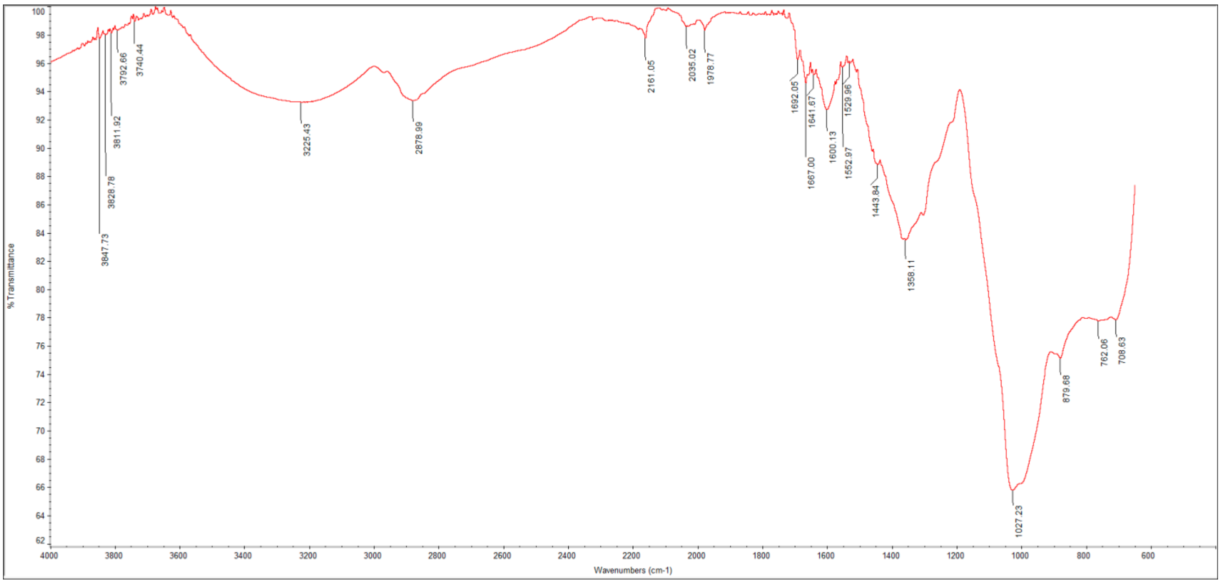
% Transmittance
Wavenumbers (cm-1)
3847.73
3828.78
3811.92
3792.66
3740.44
3225.43
2878.99
2161.05
2035.02
1978.77
1692.05
1667.00
1641.67
1600.13
1552.97
1529.96
1443.84
1358.11
1027.23
879.68
762.06
708.63

FAX 5

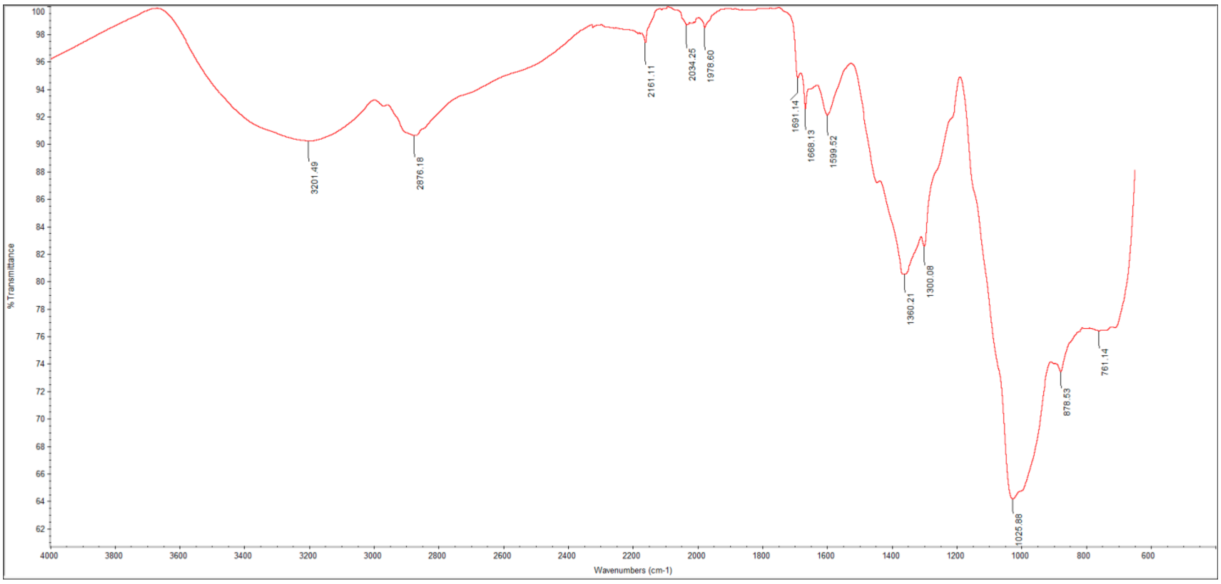

% Transmittance
Wavenumbers (cm-1)
3201.49
2876.18
2161.11
2034.25
1978.60
1691.14
1668.13
1599.52
1360.21
1300.08
1025.88
878.53
761.14

FAX 6

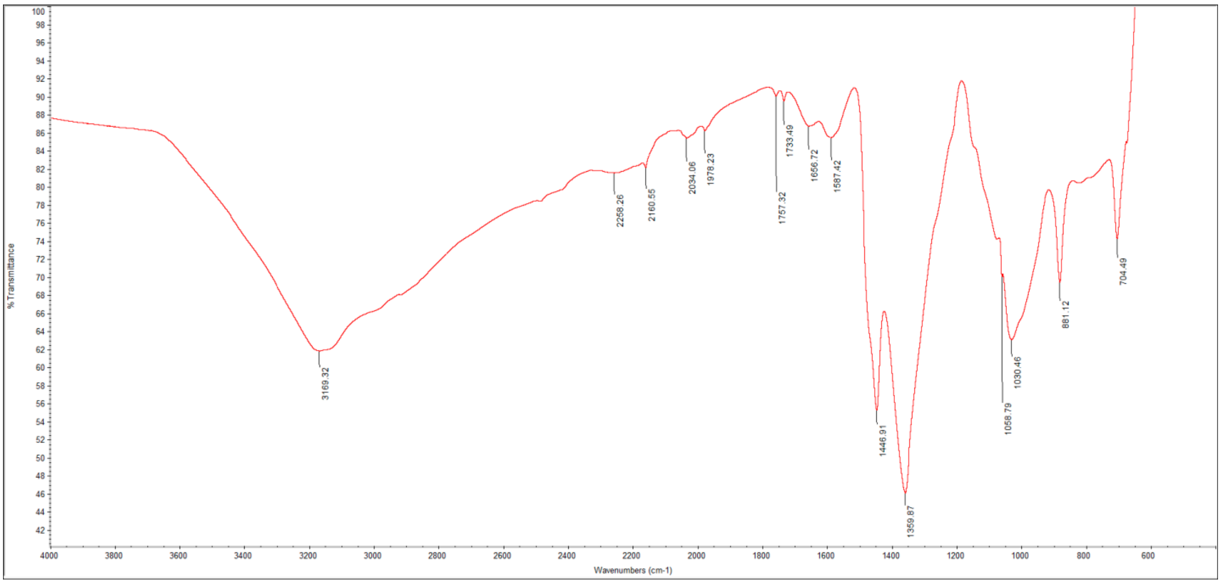

% Transmittance
Wavenumbers (cm-1)
3169.32
2258.26
2160.55
2034.06
1978.23
1757.32
1733.49
1656.72
1587.42
1446.91
1359.87
1058.79
1030.46
881.12
704.49

FAX 7

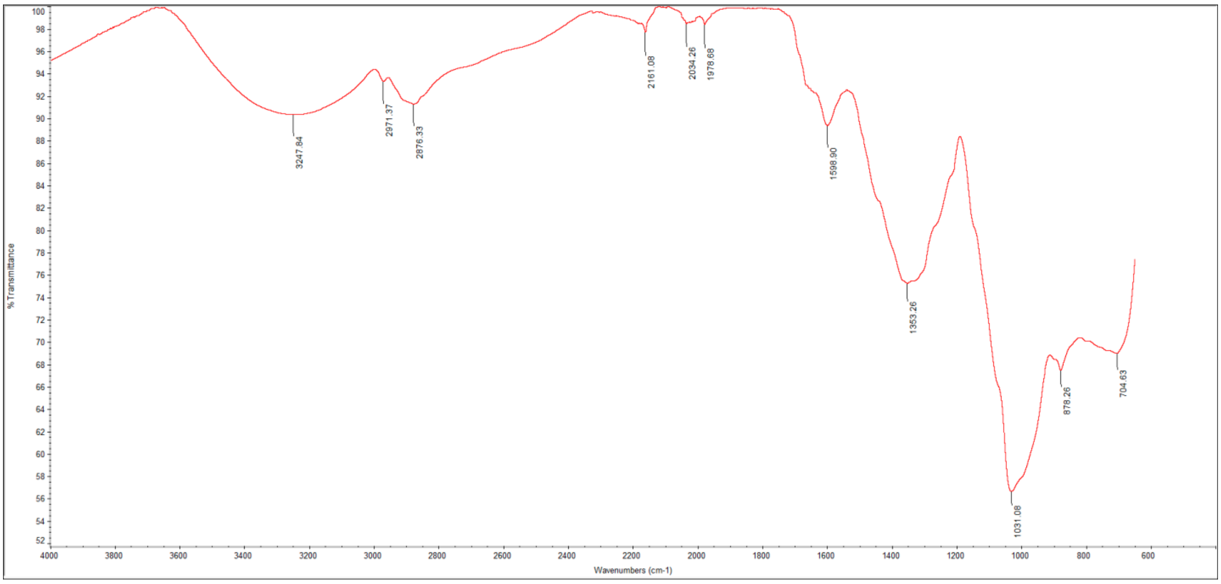

% Transmittance
Wavenumbers (cm-1)
3247.84
2971.37
2876.33
2161.08
2034.26
1978.68
1598.90
1353.26
1031.08
878.26
704.63

FAX 8

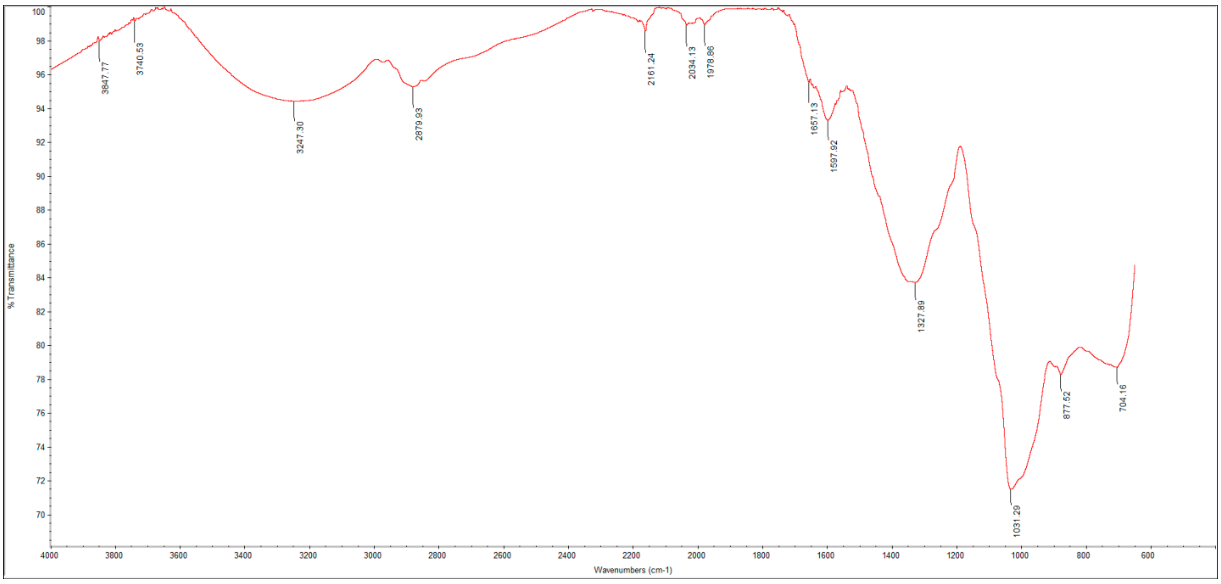

% Transmittance
Wavenumbers (cm-1)
3847.77
3740.53
3247.30
2879.93
2161.24
2034.13
1978.86
1657.13
1597.92
1327.89
1031.29
877.52
704.16

FAX 9

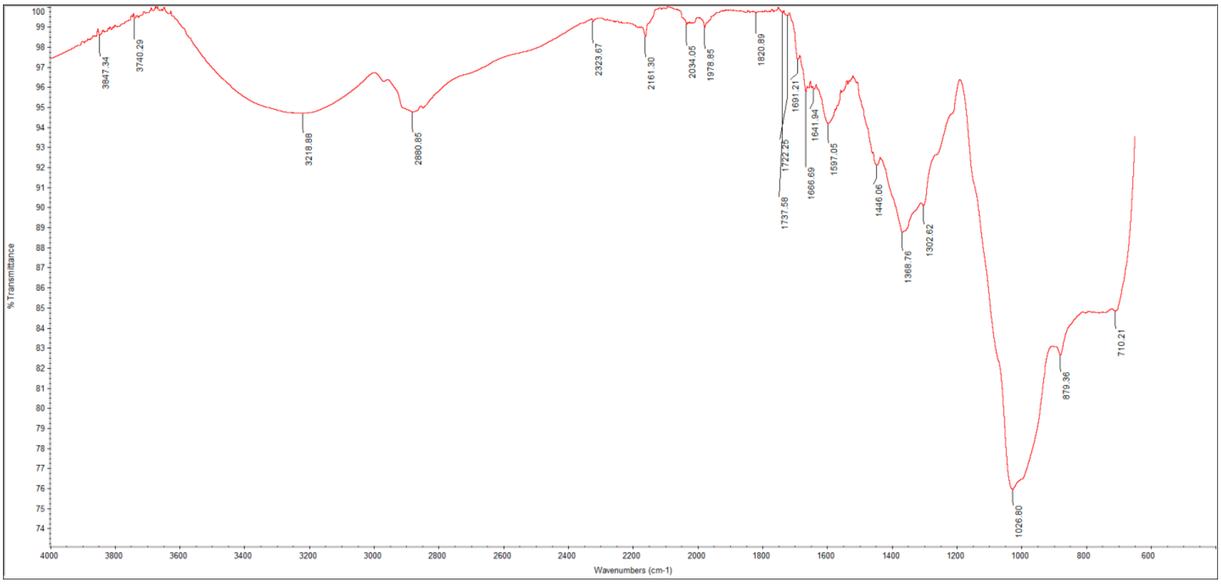

% Transmittance
100
99
98
97
96
95
94
93
92
91
90
89
88
87
86
85
84
83
82
81
80
79
78
77
76
75
74
3847.34
3740.29
3218.88
2880.85
2323.67
2161.30
2034.05
1978.85
1820.89
1737.58
1722.25
1691.21
1666.69
1641.94
1597.05
1446.06
1368.76
1302.62
1026.80
879.36
710.21
4000
3800
3600
3400
3200
3000
2800
2600
2400
2200
2000
1800
1600
1400
1200
1000
800
600
Wavenumbers (cm-1)
