# Supplementary material for: Optimized Alkaline Hydrolysis for Recovering Ferulated Arabinoxylan Biopolymers from Maize Bran with Antioxidant Functionality
Source: Polymers (Basel). 2026 Mar 12;18(6):689. doi: 10.3390/polym18060689 (PMC13030836; doi:10.3390/polym18060689)
Supplement: Supplementary file 1 [file polymers-18-00689-s001.zip › Suppllemetary file S3.pdf]

===

Acq. Operator : SYSTEM   Seq. Line : 6
Sample Operator : SYSTEM
Acq. Instrument : LC   Location : P1-A-07
Injection Date : 10/2/2024 11:51:41 AM   Inj : 1
Inj Volume : 5.000 µl
Different Inj Volume from Sample Entry! Actual Inj Volume : 10.000 µl
Acq. Method : C:\Users\Public\Documents\ChemStation\1\Data\R1Phenolics07122024\Kristin Sequence 2024-10-02 09-40-51\Ferulic Acid 300SB C18.M
Last changed : 10/2/2024 9:37:39 AM by SYSTEM
Analysis Method : C:\Users\Public\Documents\ChemStation\1\Data\R1Phenolics07122024\Kristin Sequence 2024-10-02 09-40-51\Ferulic Acid 300SB C18.M (Sequence Method)
Last changed : 10/3/2024 8:35:21 AM by SYSTEM
(modified after loading)

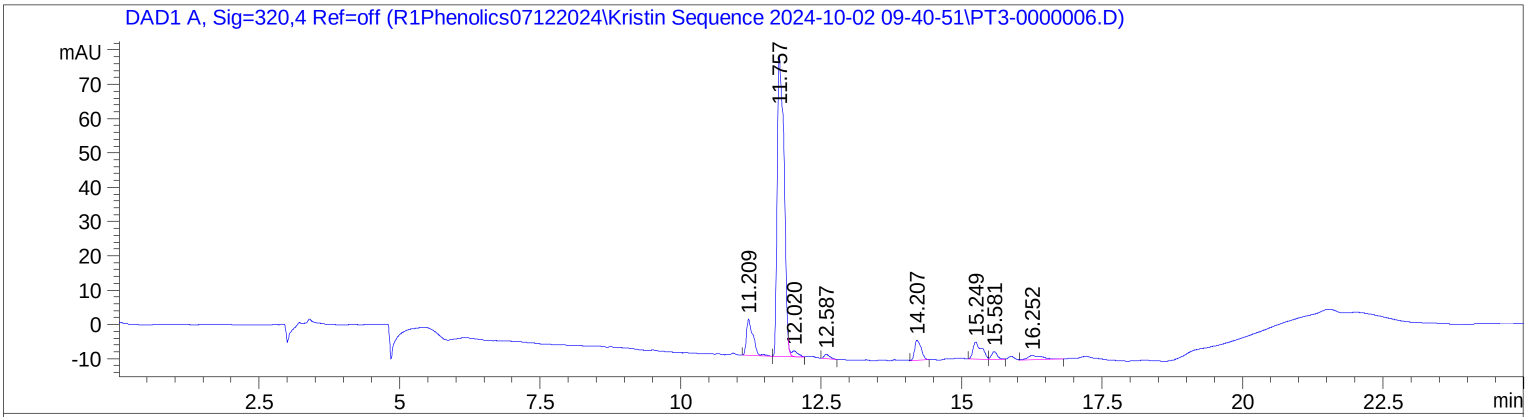

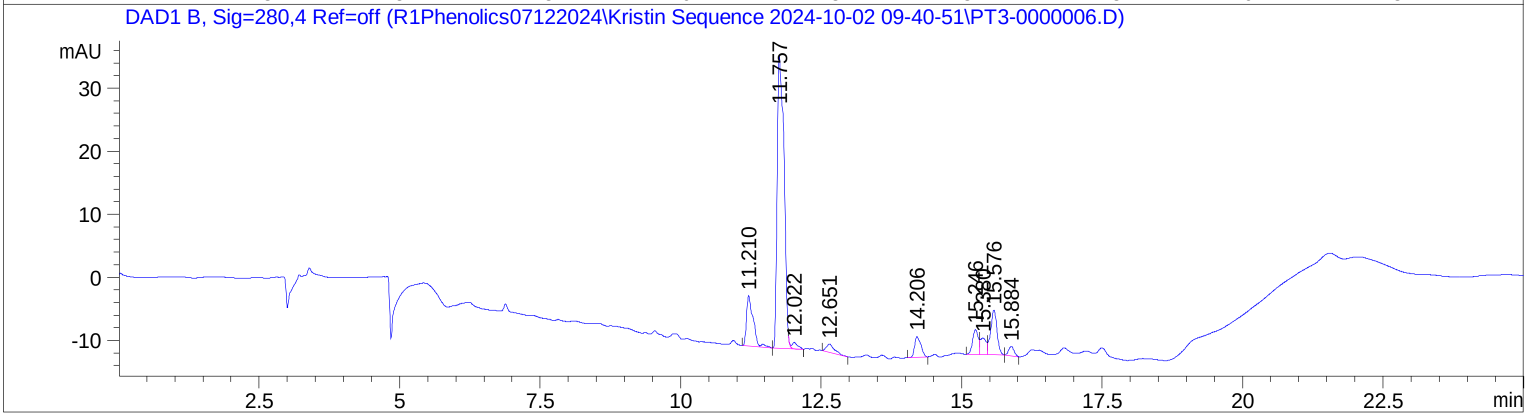

===

Fraction Information

===

No Fractions found.

===

===

===

External Standard Report

===

Sorted By : Signal
Calib. Data Modified : Thursday, October 3, 20248:35:21 AM
Multiplier : 1.0000
Dilution : 1.0000
Do not use Multiplier & Dilution Factor with ISTDs

Signal 1: DAD1 A, Sig=320,4 Ref=off

RetTime [min]	Type	Area [mAU*s]	Amt/Area	Amount [ng/ul]	Grp	Name
11.757	BV R	743.78094	2.92761e-2	21.77500		Ferulic Acid

Totals : 21.77500

Signal 2: DAD1 B, Sig=280,4 Ref=off

RetTime [min]	Type	Area [mAU*s]	Amt/Area	Amount [ng/ul]	Grp	Name
15.884	BB	9.68212	7.30072e-2	7.06864e-1		Cinnamic Acid

Totals : 7.06864e-1

*** End of Report ***

Data File C:\USERS\P...HENOLICS07122024\KRISTIN SEQUENCE 2024-10-02 09-40-51\PT3-0000015.D
Sample Name: FAX 10

===

Acq. Operator : SYSTEM   Seq. Line : 15
Sample Operator : SYSTEM
Acq. Instrument : LC   Location : P1-B-05
Injection Date : 10/2/2024 3:45:25 PM   Inj : 1
Inj Volume : 5.000 µl
Different Inj Volume from Sample Entry! Actual Inj Volume : 10.000 µl
Acq. Method : C:\Users\Public\Documents\ChemStation\1\Data\R1Phenolics07122024\Kristin Sequence 2024-10-02 09-40-51\Ferulic Acid 300SB C18.M
Last changed : 10/2/2024 9:37:39 AM by SYSTEM
Analysis Method : C:\Users\Public\Documents\ChemStation\1\Data\R1Phenolics07122024\Kristin Sequence 2024-10-02 09-40-51\Ferulic Acid 300SB C18.M (Sequence Method)
Last changed : 10/3/2024 8:35:21 AM by SYSTEM
(modified after loading)

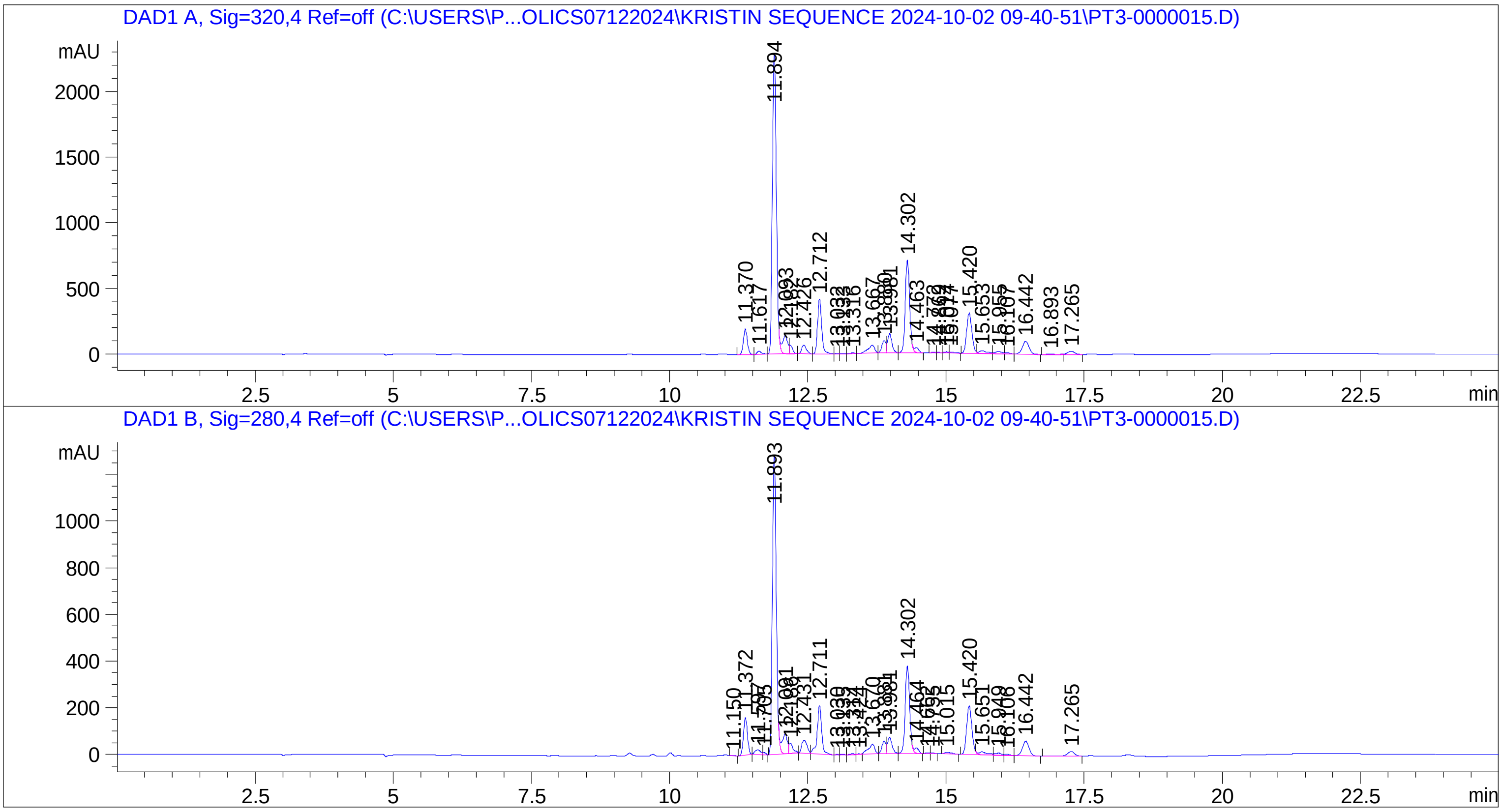

===

Fraction Information

===

No Fractions found.

===

===

===

External Standard Report

===

Sorted By : Signal
Calib. Data Modified : Thursday, October 3, 20248:35:21 AM
Multiplier : 1.0000
Dilution : 1.0000
Do not use Multiplier & Dilution Factor with ISTDs

Signal 1: DAD1 A, Sig=320,4 Ref=off

RetTime [min]	Type	Area [mAU*s]	Amt/Area	Amount [ng/ul]	Grp	Name
11.894	BV R	1.16207e4	2.89381e-2	336.28047		Ferulic Acid
Totals :				336.28047		

Signal 2: DAD1 B, Sig=280,4 Ref=off

RetTime [min]	Type	Area [mAU*s]	Amt/Area	Amount [ng/ul]	Grp	Name
16.106	VB E	33.42316	3.42550e-2	1.14491		Cinnamic Acid
Totals :				1.14491		

==

*** End of Report ***

Data File C:\USERS\P...HENOLICS07122024\KRISTIN SEQUENCE 2024-10-02 09-40-51\PT3-0000016.D
Sample Name: FAX 11

===

Acq. Operator : SYSTEM Seq. Line : 16
Sample Operator : SYSTEM
Acq. Instrument : LC Location : P1-B-06
Injection Date : 10/2/2024 4:11:24 PM Inj : 1
Inj Volume : 5.000 µl
Different Inj Volume from Sample Entry! Actual Inj Volume : 10.000 µl
Acq. Method : C:\Users\Public\Documents\ChemStation\1\Data\R1Phenolics07122024\Kristin Sequence 2024-10-02 09-40-51\Ferulic Acid 300SB C18.M
Last changed : 10/2/2024 9:37:39 AM by SYSTEM
Analysis Method : C:\Users\Public\Documents\ChemStation\1\Data\R1Phenolics07122024\Kristin Sequence 2024-10-02 09-40-51\Ferulic Acid 300SB C18.M (Sequence Method)
Last changed : 10/3/2024 8:35:21 AM by SYSTEM
(modified after loading)

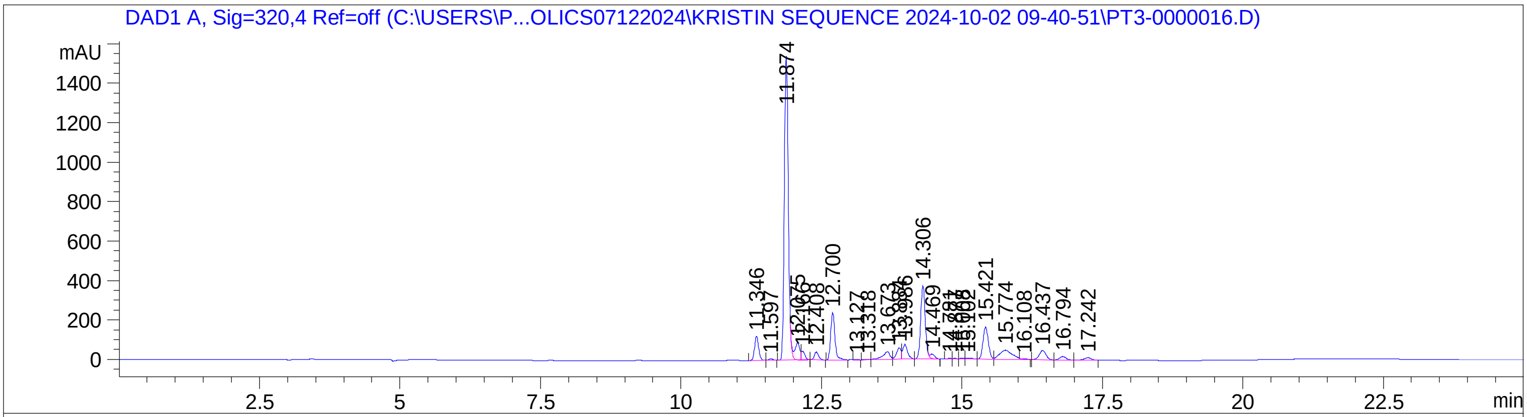

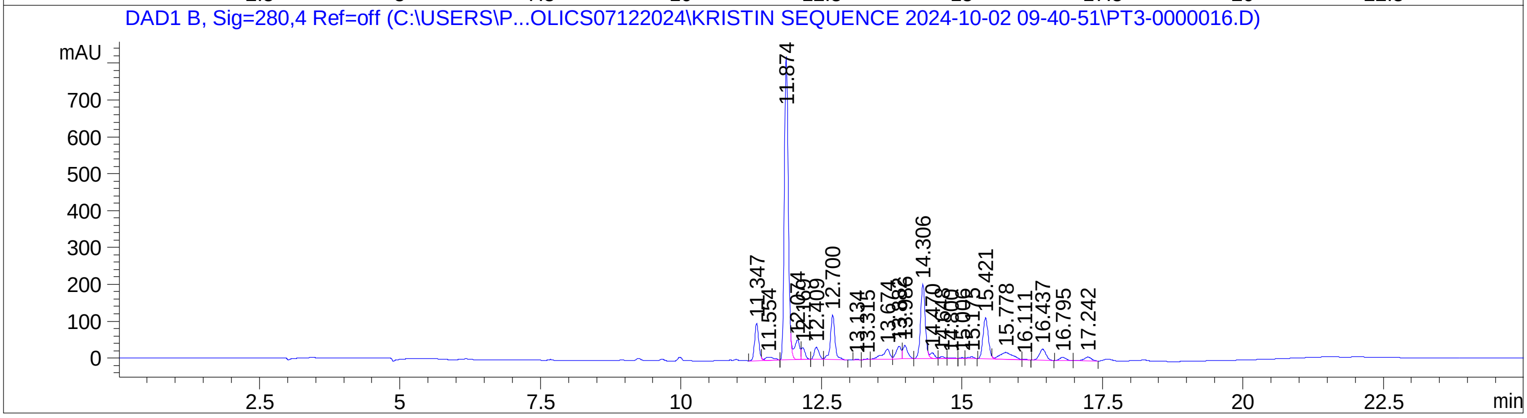

==

Fraction Information

==

No Fractions found.

==

==

===

External Standard Report

===

Sorted By : Signal
Calib. Data Modified : Thursday, October 3, 20248:35:21 AM
Multiplier : 1.0000
Dilution : 1.0000
Do not use Multiplier & Dilution Factor with ISTDs

Signal 1: DAD1 A, Sig=320,4 Ref=off

RetTime [min]	Type	Area [mAU*s]	Amt/Area	Amount [ng/ul]	Grp	Name
11.874	BV R	7625.12793	2.89503e-2	220.74942		Ferulic Acid

Totals : 220.74942

Signal 2: DAD1 B, Sig=280,4 Ref=off

RetTime [min]	Type	Area [mAU*s]	Amt/Area	Amount [ng/ul]	Grp	Name
16.111	VB E	12.63881	6.02445e-2	7.61418e-1		Cinnamic Acid

Totals : 7.61418e-1

===

*** End of Report ***

Data File C:\USERS\P...HENOLICS07122024\KRISTIN SEQUENCE 2024-10-02 09-40-51\PT3-0000017.D
Sample Name: FAX 12

===
Acq. Operator : SYSTEM   Seq. Line : 17
Sample Operator : SYSTEM
Acq. Instrument : LC   Location : P1-B-07
Injection Date : 10/2/2024 4:37:22 PM   Inj : 1
Inj Volume : 5.000 µl
Different Inj Volume from Sample Entry! Actual Inj Volume : 10.000 µl
Acq. Method : C:\Users\Public\Documents\ChemStation\1\Data\R1Phenolics07122024\Kristin Sequence 2024-10-02 09-40-51\Ferulic Acid 300SB C18.M
Last changed : 10/2/2024 9:37:39 AM by SYSTEM
Analysis Method : C:\Users\Public\Documents\ChemStation\1\Data\R1Phenolics07122024\Kristin Sequence 2024-10-02 09-40-51\Ferulic Acid 300SB C18.M (Sequence Method)
Last changed : 10/3/2024 8:35:21 AM by SYSTEM
(modified after loading)

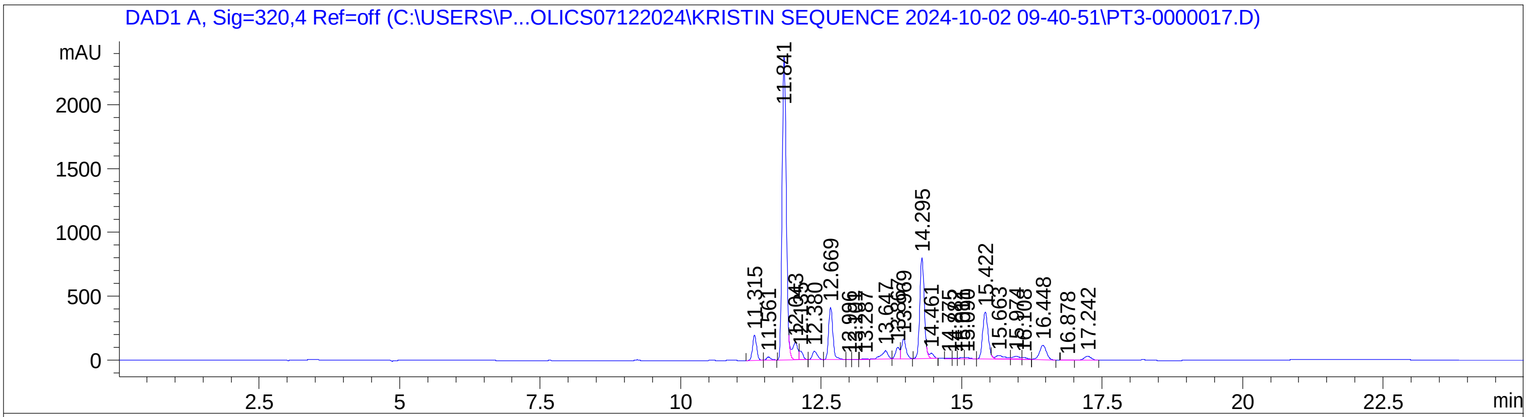

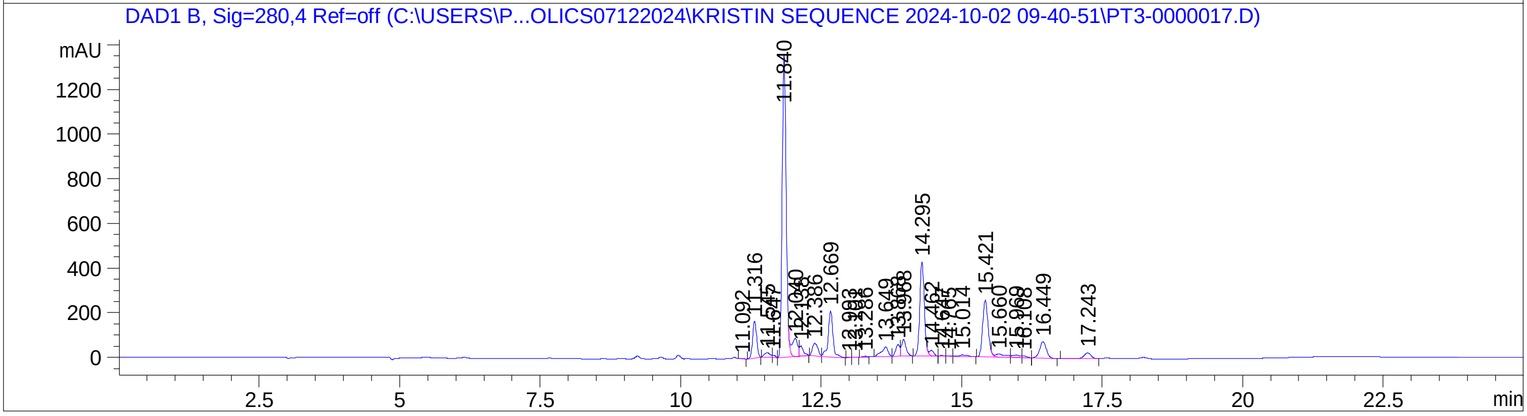

===
Fraction Information
===
No Fractions found.
===
===
===
External Standard Report
===

Sorted By : Signal
Calib. Data Modified : Thursday, October 3, 20248:35:21 AM
Multiplier : 1.0000
Dilution : 1.0000
Do not use Multiplier & Dilution Factor with ISTDs

Signal 1: DAD1 A, Sig=320,4 Ref=off

RetTime [min]	Type	Area [mAU*s]	Amt/Area	Amount [ng/ul]	Grp	Name
11.841	BV R	1.23432e4	2.89368e-2	357.17283		Ferulic Acid

Totals : 357.17283

Signal 2: DAD1 B, Sig=280,4 Ref=off

RetTime [min]	Type	Area [mAU*s]	Amt/Area	Amount [ng/ul]	Grp	Name
16.108	VB E	42.92803	3.07558e-2	1.32028		Cinnamic Acid

Totals : 1.32028

===

*** End of Report ***

===

Acq. Operator : SYSTEM Seq. Line : 18
Sample Operator : SYSTEM
Acq. Instrument : LC Location : P1-B-08
Injection Date : 10/2/2024 5:03:20 PM Inj : 1
Inj Volume : 5.000 µl
Different Inj Volume from Sample Entry! Actual Inj Volume : 10.000 µl
Acq. Method : C:\Users\Public\Documents\ChemStation\1\Data\R1Phenolics07122024\Kristin Sequence 2024-10-02 09-40-51\Ferulic Acid 300SB C18.M
Last changed : 10/2/2024 9:37:39 AM by SYSTEM
Analysis Method : C:\Users\Public\Documents\ChemStation\1\Data\R1Phenolics07122024\Kristin Sequence 2024-10-02 09-40-51\Ferulic Acid 300SB C18.M (Sequence Method)
Last changed : 10/3/2024 8:35:21 AM by SYSTEM
(modified after loading)

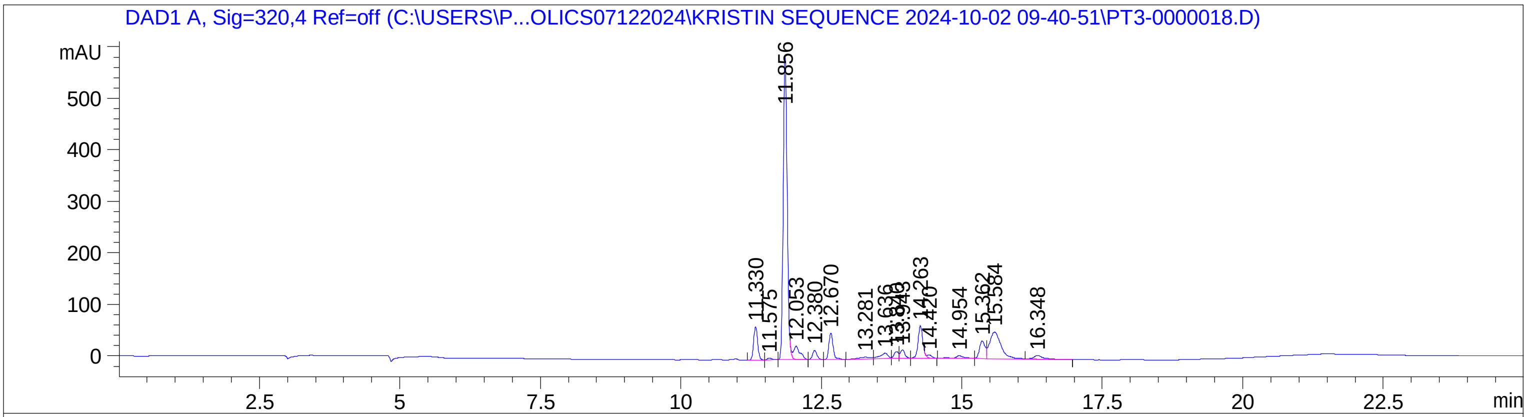

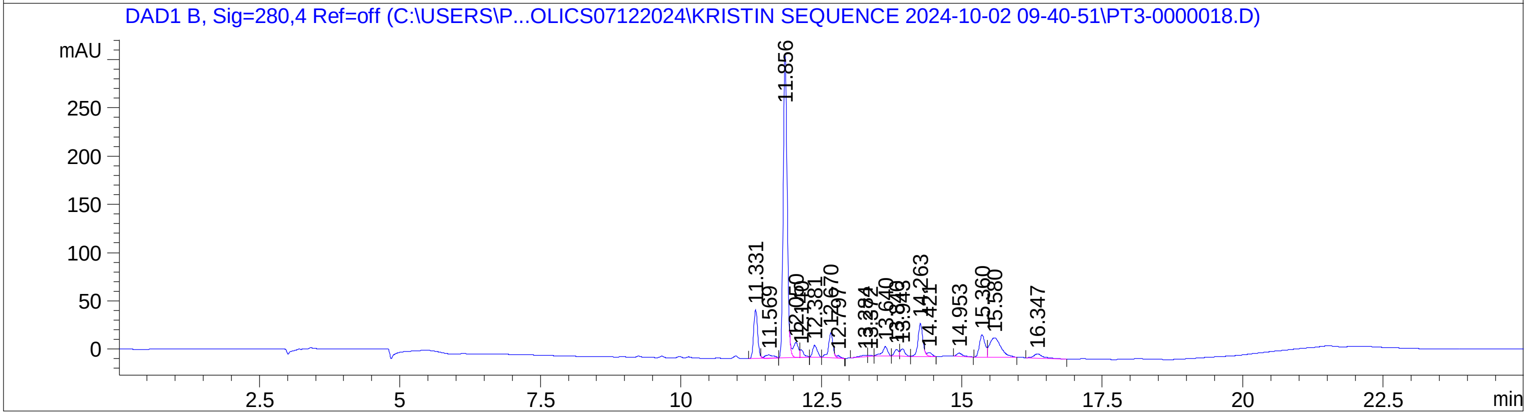

===

Fraction Information

===

No Fractions found.

===

===

===

External Standard Report

===

Sorted By : Signal
Calib. Data Modified : Thursday, October 3, 20248:35:21 AM
Multiplier : 1.0000
Dilution : 1.0000
Do not use Multiplier & Dilution Factor with ISTDs

Signal 1: DAD1 A, Sig=320,4 Ref=off

RetTime [min]	Type	Area [mAU*s]	Amt/Area	Amount [ng/ul]	Grp	Name
11.856	BV R	2918.43970	2.90071e-2	84.65534		Ferulic Acid

Totals : 84.65534

Signal 2: DAD1 B, Sig=280,4 Ref=off

RetTime [min]	Type	Area [mAU*s]	Amt/Area	Amount [ng/ul]	Grp	Name
16.347	BB	48.50798	2.93403e-2	1.42324		Cinnamic Acid

Totals : 1.42324

*** End of Report ***

===

Acq. Operator : SYSTEM Seq. Line : 19
Sample Operator : SYSTEM
Acq. Instrument : LC Location : P1-B-09
Injection Date : 10/2/2024 5:29:18 PM Inj : 1
Inj Volume : 5.000 µl
Different Inj Volume from Sample Entry! Actual Inj Volume : 10.000 µl
Acq. Method : C:\Users\Public\Documents\ChemStation\1\Data\R1Phenolics07122024\Kristin Sequence 2024-10-02 09-40-51\Ferulic Acid 300SB C18.M
Last changed : 10/2/2024 9:37:39 AM by SYSTEM
Analysis Method : C:\Users\Public\Documents\ChemStation\1\Data\R1Phenolics07122024\Kristin Sequence 2024-10-02 09-40-51\Ferulic Acid 300SB C18.M (Sequence Method)
Last changed : 10/3/2024 8:35:21 AM by SYSTEM
(modified after loading)

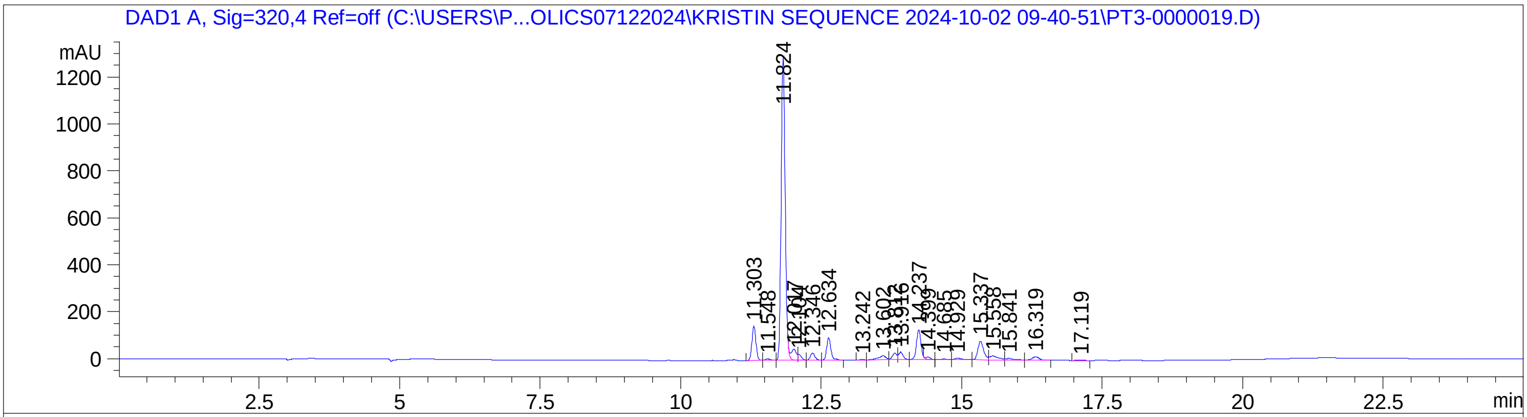

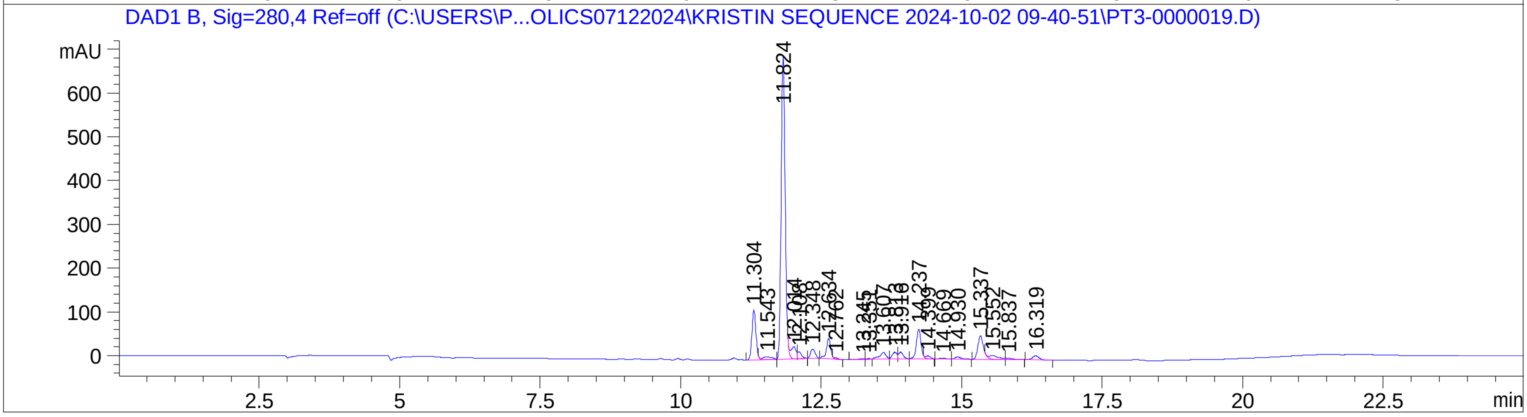

===

Fraction Information

===

No Fractions found.

===

===

===

External Standard Report

===

Sorted By : Signal
Calib. Data Modified : Thursday, October 3, 20248:35:21 AM
Multiplier : 1.0000
Dilution : 1.0000
Do not use Multiplier & Dilution Factor with ISTDs

Signal 1: DAD1 A, Sig=320,4 Ref=off

RetTime [min]	Type	Area [mAU*s]	Amt/Area	Amount [ng/ul]	Grp	Name
11.824	BV R	6473.57324	2.89565e-2	187.45217		Ferulic Acid

Totals : 187.45217

Signal 2: DAD1 B, Sig=280,4 Ref=off

RetTime [min]	Type	Area [mAU*s]	Amt/Area	Amount [ng/ul]	Grp	Name
16.319	BB	83.63685	2.47666e-2	2.07140		Cinnamic Acid

Totals : 2.07140

==

*** End of Report ***

Data File C:\USERS\P...HENOLICS07122024\KRISTIN SEQUENCE 2024-10-02 09-40-51\PT3-0000020.D
Sample Name: FAX 15

===

Acq. Operator : SYSTEM Seq. Line : 20
Sample Operator : SYSTEM
Acq. Instrument : LC Location : P1-B-10
Injection Date : 10/2/2024 5:55:17 PM Inj : 1
Inj Volume : 5.000 µl
Different Inj Volume from Sample Entry! Actual Inj Volume : 10.000 µl
Acq. Method : C:\Users\Public\Documents\ChemStation\1\Data\R1Phenolics07122024\Kristin Sequence 2024-10-02 09-40-51\Ferulic Acid 300SB C18.M
Last changed : 10/2/2024 9:37:39 AM by SYSTEM
Analysis Method : C:\Users\Public\Documents\ChemStation\1\Data\R1Phenolics07122024\Kristin Sequence 2024-10-02 09-40-51\Ferulic Acid 300SB C18.M (Sequence Method)
Last changed : 10/3/2024 8:35:21 AM by SYSTEM
(modified after loading)

DAD1 A, Sig=320,4 Ref=off (C:\USERS\P...OLICS07122024\KRISTIN SEQUENCE 2024-10-02 09-40-51\PT3-0000020.D)

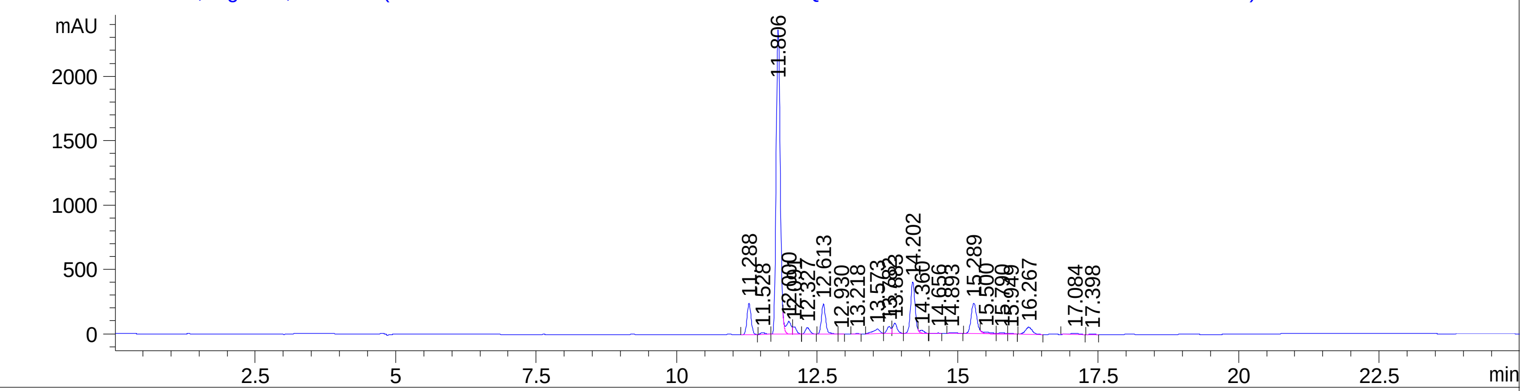

DAD1 B, Sig=280,4 Ref=off (C:\USERS\P...OLICS07122024\KRISTIN SEQUENCE 2024-10-02 09-40-51\PT3-0000020.D)

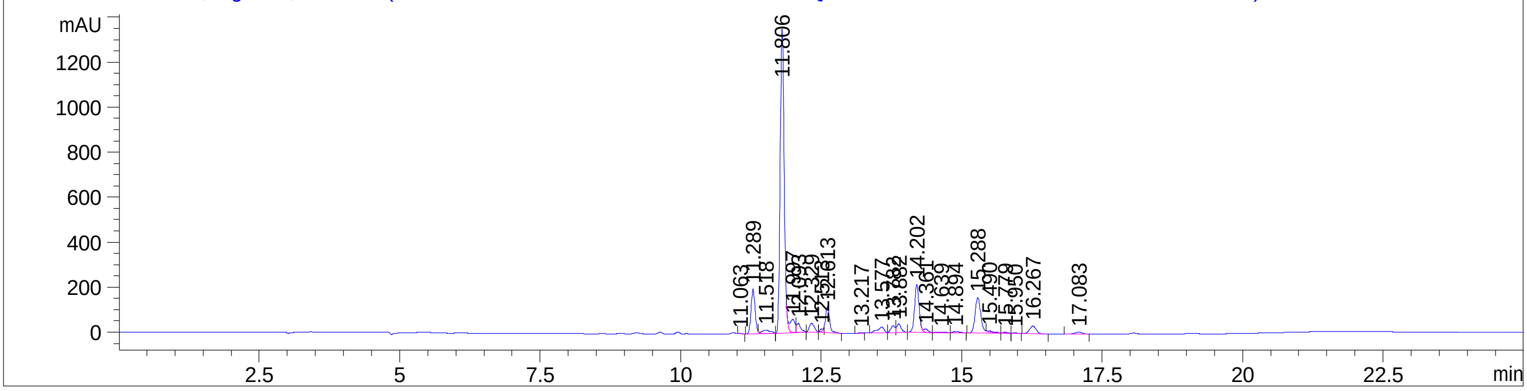

===

Fraction Information

===

No Fractions found.

===

===

===

External Standard Report

===

Sorted By : Signal
Calib. Data Modified : Thursday, October 3, 20248:35:21 AM
Multiplier : 1.0000
Dilution : 1.0000
Do not use Multiplier & Dilution Factor with ISTDs

Signal 1: DAD1 A, Sig=320,4 Ref=off

RetTime [min]	Type	Area [mAU*s]	Amt/Area	Amount [ng/ul]	Grp	Name
11.806	BV R	1.20951e4	2.89372e-2	349.99842		Ferulic Acid

Totals : 349.99842

Signal 2: DAD1 B, Sig=280,4 Ref=off

RetTime [min]	Type	Area [mAU*s]	Amt/Area	Amount [ng/ul]	Grp	Name
16.267	BB	294.77734	2.02429e-2	5.96715		Cinnamic Acid

Totals : 5.96715

*** End of Report ***

Data File C:\USERS\P...HENOLICS07122024\KRISTIN SEQUENCE 2024-10-02 09-40-51\PT3-0000021.D
Sample Name: FAX 16

===
Acq. Operator : SYSTEM Seq. Line : 21
Sample Operator : SYSTEM
Acq. Instrument : LC Location : P1-B-11
Injection Date : 10/2/2024 6:21:17 PM Inj : 1
Inj Volume : 5.000 µl
Different Inj Volume from Sample Entry! Actual Inj Volume : 10.000 µl
Acq. Method : C:\Users\Public\Documents\ChemStation\1\Data\R1Phenolics07122024\Kristin Sequence 2024-10-02 09-40-51\Ferulic Acid 300SB C18.M
Last changed : 10/2/2024 9:37:39 AM by SYSTEM
Analysis Method : C:\Users\Public\Documents\ChemStation\1\Data\R1Phenolics07122024\Kristin Sequence 2024-10-02 09-40-51\Ferulic Acid 300SB C18.M (Sequence Method)
Last changed : 10/3/2024 8:35:21 AM by SYSTEM
(modified after loading)

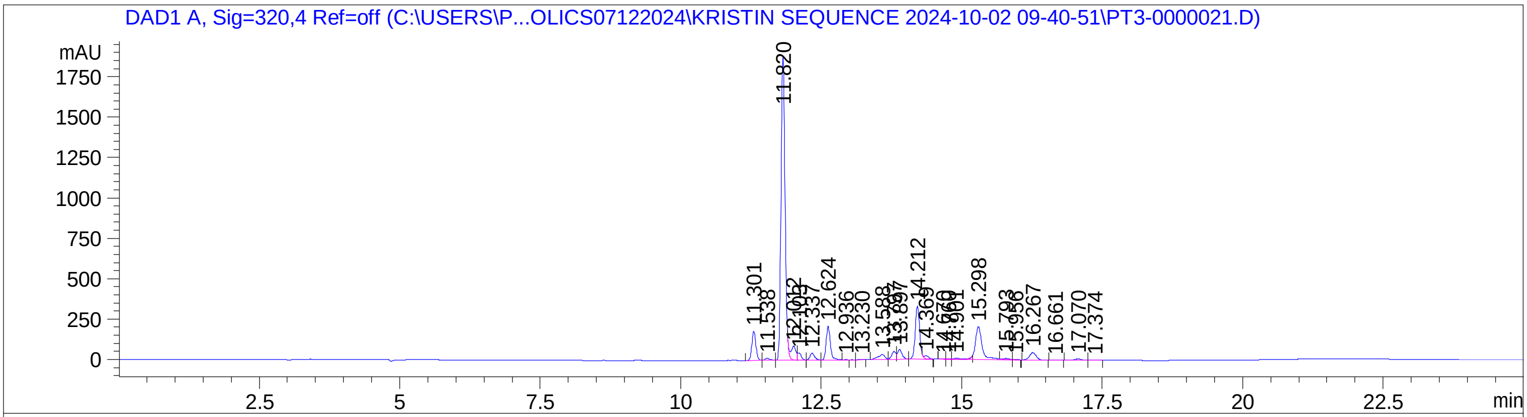

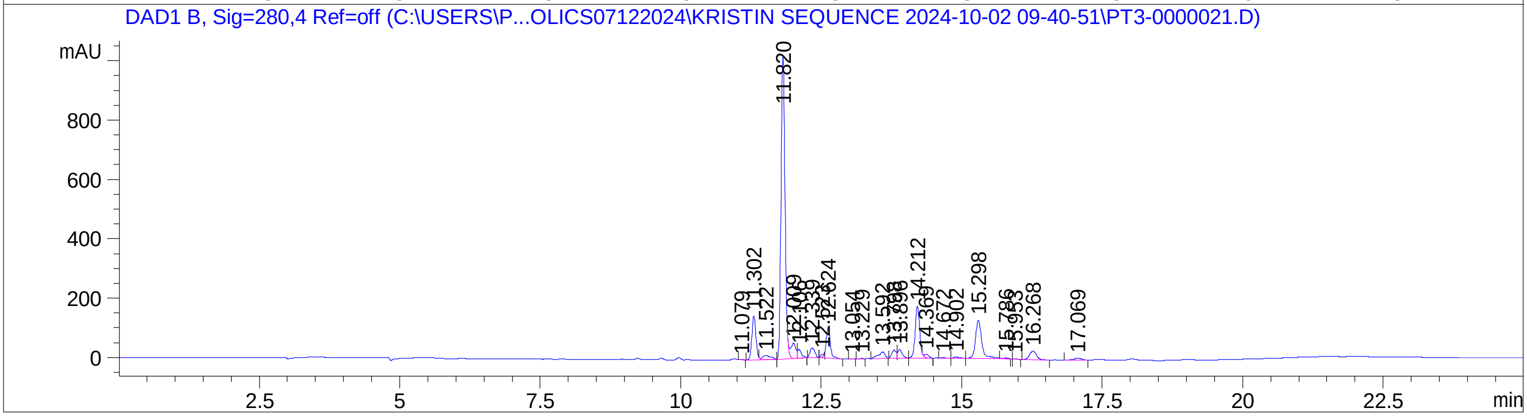

===
Fraction Information
===
No Fractions found.
===
===

===
External Standard Report
===

Sorted By : Signal
Calib. Data Modified : Thursday, October 3, 20248:35:21 AM
Multiplier : 1.0000
Dilution : 1.0000
Do not use Multiplier & Dilution Factor with ISTDs

Signal 1: DAD1 A, Sig=320,4 Ref=off

RetTime [min]	Type	Area [mAU*s]	Amt/Area	Amount [ng/ul]	Grp	Name
11.820	BV R	9414.33887	2.89436e-2	272.48452		Ferulic Acid

Totals : 272.48452

Signal 2: DAD1 B, Sig=280,4 Ref=off

RetTime [min]	Type	Area [mAU*s]	Amt/Area	Amount [ng/ul]	Grp	Name
16.268	BB	251.22992	2.05535e-2	5.16366		Cinnamic Acid

Totals : 5.16366

===

*** End of Report ***

===

Acq. Operator : SYSTEM Seq. Line : 22
Sample Operator : SYSTEM
Acq. Instrument : LC Location : P1-C-01
Injection Date : 10/2/2024 6:47:17 PM Inj : 1
Inj Volume : 5.000 µl
Different Inj Volume from Sample Entry! Actual Inj Volume : 10.000 µl
Acq. Method : C:\Users\Public\Documents\ChemStation\1\Data\R1Phenolics07122024\Kristin Sequence 2024-10-02 09-40-51\Ferulic Acid 300SB C18.M
Last changed : 10/2/2024 9:37:39 AM by SYSTEM
Analysis Method : C:\Users\Public\Documents\ChemStation\1\Data\R1Phenolics07122024\Kristin Sequence 2024-10-02 09-40-51\Ferulic Acid 300SB C18.M (Sequence Method)
Last changed : 10/3/2024 8:35:21 AM by SYSTEM
(modified after loading)

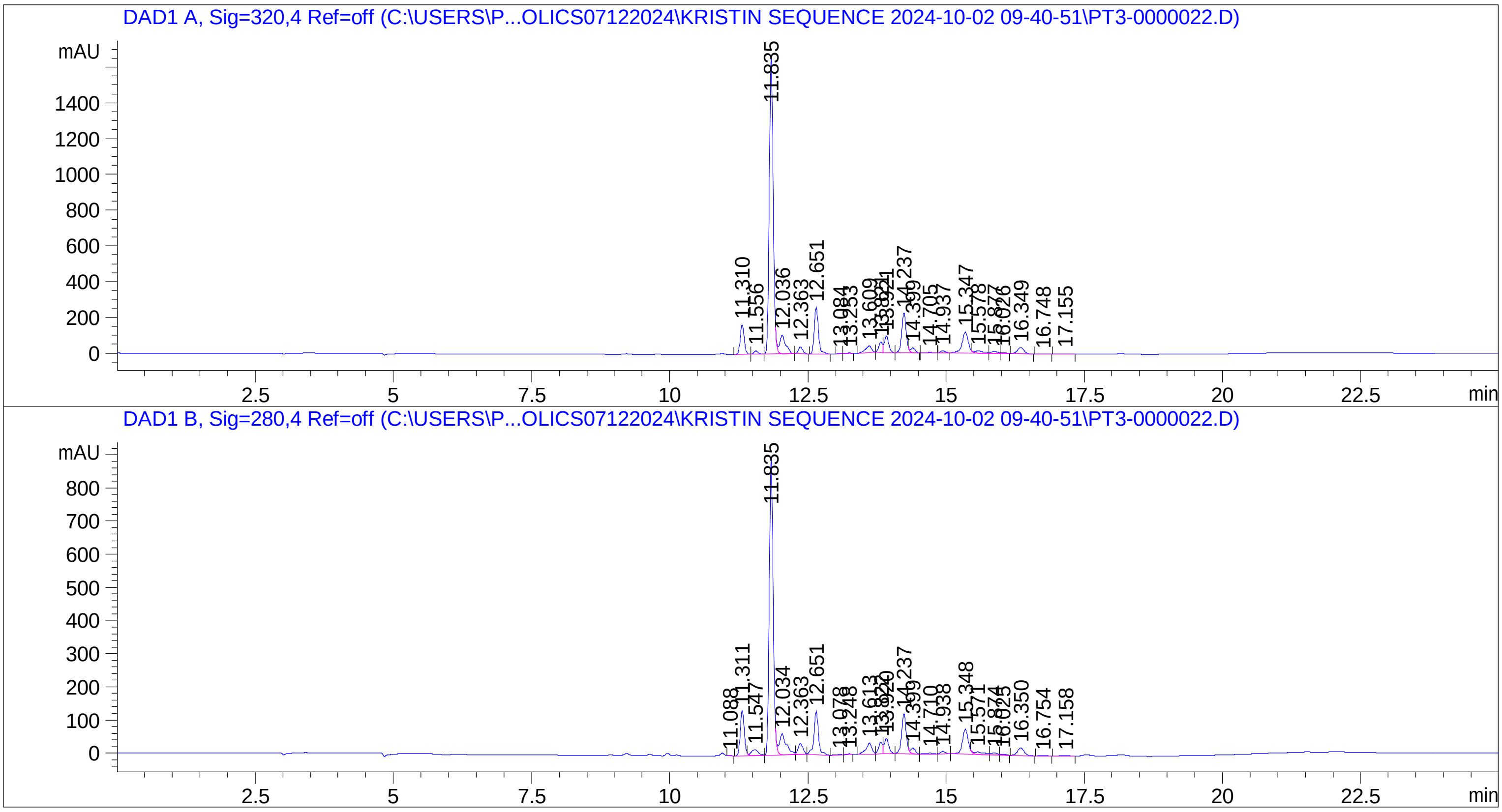

===

Fraction Information

===

No Fractions found.

===

===

===

External Standard Report

===

Sorted By : Signal
Calib. Data Modified : Thursday, October 3, 20248:35:21 AM
Multiplier : 1.0000
Dilution : 1.0000
Do not use Multiplier & Dilution Factor with ISTDs

Signal 1: DAD1 A, Sig=320,4 Ref=off

RetTime [min]	Type	Area [mAU*s]	Amt/Area	Amount [ng/ul]	Grp	Name
11.835	VV R	8362.80371	2.89472e-2	242.07934		Ferulic Acid

Totals : 242.07934

Signal 2: DAD1 B, Sig=280,4 Ref=off

RetTime [min]	Type	Area [mAU*s]	Amt/Area	Amount [ng/ul]	Grp	Name
16.025	VB E	24.50464	4.00069e-2	9.80355e-1		Cinnamic Acid

Totals : 9.80355e-1

*** End of Report ***

===

Acq. Operator : SYSTEM Seq. Line : 23
Sample Operator : SYSTEM
Acq. Instrument : LC Location : P1-C-02
Injection Date : 10/2/2024 7:13:15 PM Inj : 1
Inj Volume : 5.000 µl
Different Inj Volume from Sample Entry! Actual Inj Volume : 10.000 µl
Acq. Method : C:\Users\Public\Documents\ChemStation\1\Data\R1Phenolics07122024\Kristin Sequence 2024-10-02 09-40-51\Ferulic Acid 300SB C18.M
Last changed : 10/2/2024 9:37:39 AM by SYSTEM
Analysis Method : C:\Users\Public\Documents\ChemStation\1\Data\R1Phenolics07122024\Kristin Sequence 2024-10-02 09-40-51\Ferulic Acid 300SB C18.M (Sequence Method)
Last changed : 10/3/2024 8:35:21 AM by SYSTEM
(modified after loading)

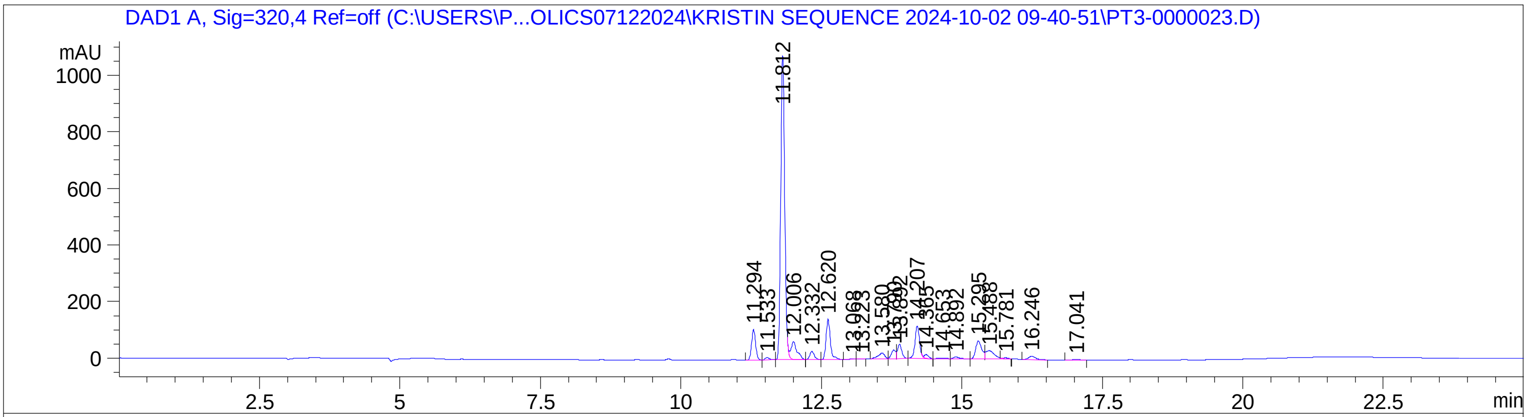

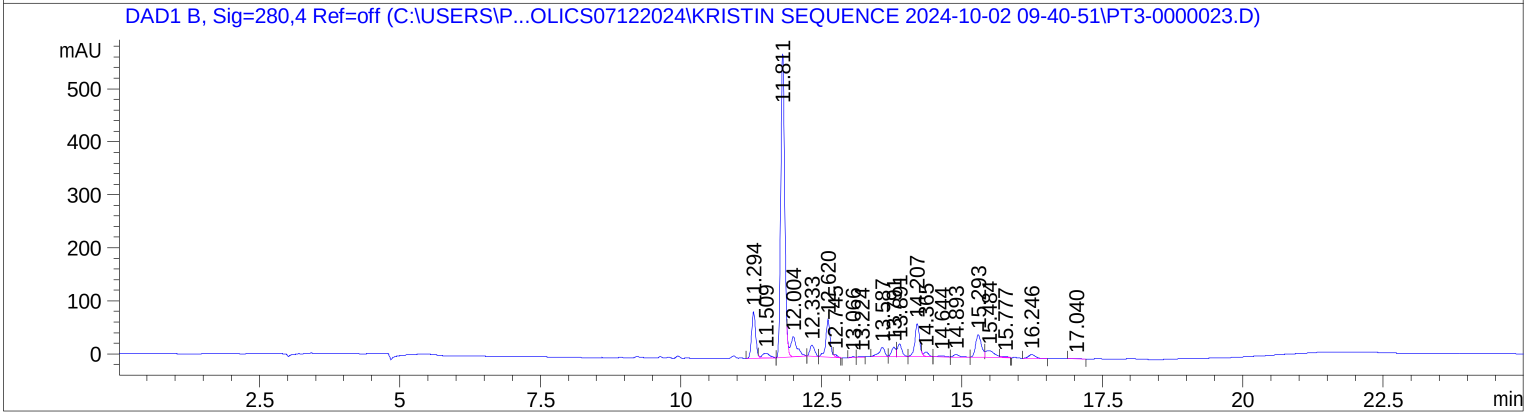

===

Fraction Information

===

No Fractions found.

===

===

===

External Standard Report

===

Sorted By : Signal
Calib. Data Modified : Thursday, October 3, 20248:35:21 AM
Multiplier : 1.0000
Dilution : 1.0000
Do not use Multiplier & Dilution Factor with ISTDs

Signal 1: DAD1 A, Sig=320,4 Ref=off

RetTime [min]	Type	Area [mAU*s]	Amt/Area	Amount [ng/ul]	Grp	Name
11.812	BV R	5336.32227	2.89654e-2	154.56851		Ferulic Acid

Totals : 154.56851

Signal 2: DAD1 B, Sig=280,4 Ref=off

RetTime [min]	Type	Area [mAU*s]	Amt/Area	Amount [ng/ul]	Grp	Name
16.246	BB	64.10494	2.66909e-2	1.71102		Cinnamic Acid

Totals : 1.71102

*** End of Report ***

Data File C:\USERS\P...HENOLICS07122024\KRISTIN SEQUENCE 2024-10-02 09-40-51\PT3-0000024.D
Sample Name: FAX 19

===

Acq. Operator : SYSTEM Seq. Line : 24
Sample Operator : SYSTEM
Acq. Instrument : LC Location : P1-C-03
Injection Date : 10/2/2024 7:39:13 PM Inj : 1
Inj Volume : 5.000 µl
Different Inj Volume from Sample Entry! Actual Inj Volume : 10.000 µl
Acq. Method : C:\Users\Public\Documents\ChemStation\1\Data\R1Phenolics07122024\Kristin Sequence 2024-10-02 09-40-51\Ferulic Acid 300SB C18.M
Last changed : 10/2/2024 9:37:39 AM by SYSTEM
Analysis Method : C:\Users\Public\Documents\ChemStation\1\Data\R1Phenolics07122024\Kristin Sequence 2024-10-02 09-40-51\Ferulic Acid 300SB C18.M (Sequence Method)
Last changed : 10/3/2024 8:35:21 AM by SYSTEM
(modified after loading)

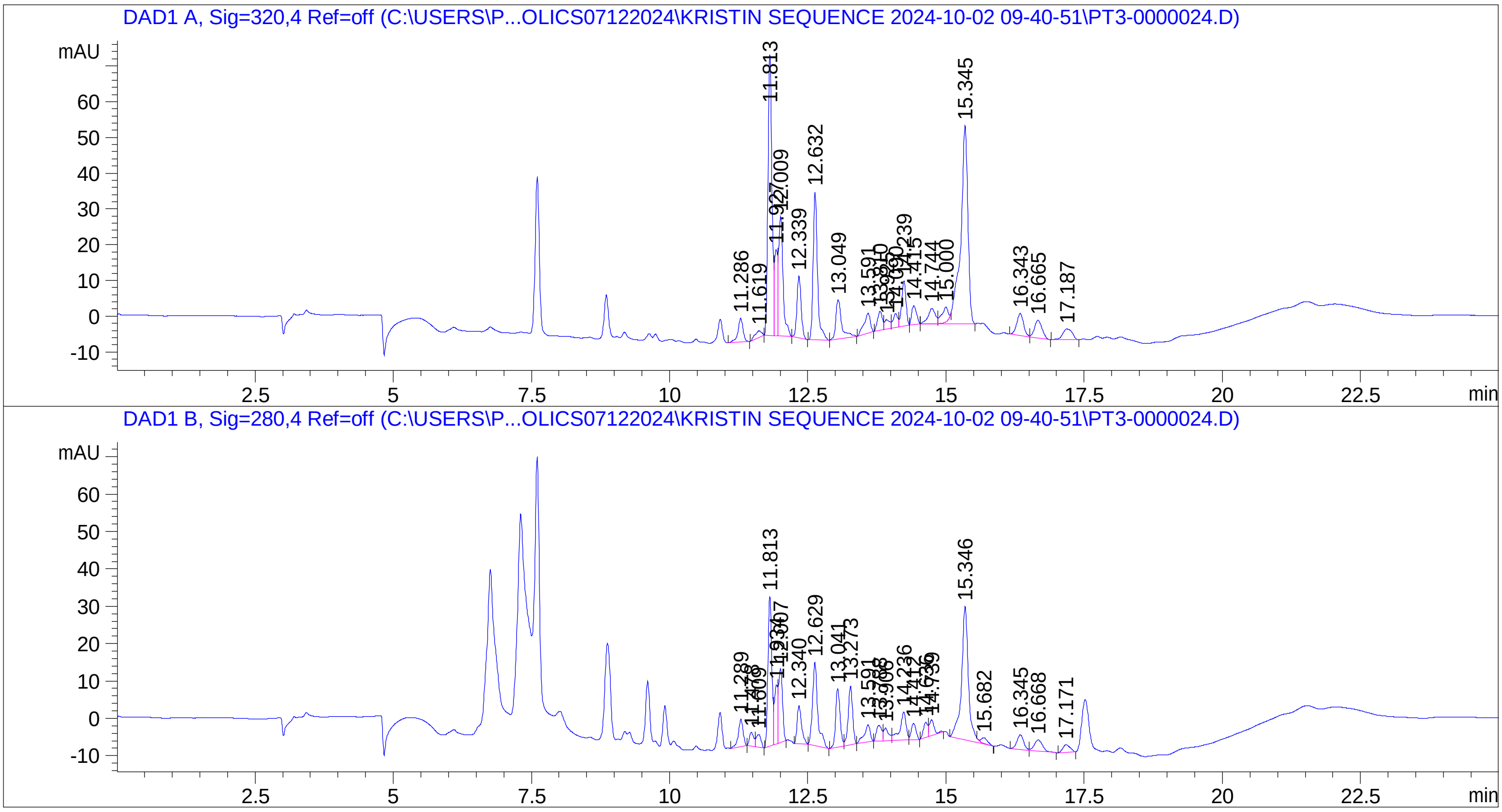

===

Fraction Information

===

No Fractions found.

===

===

===

External Standard Report

===

Sorted By : Signal
Calib. Data Modified : Thursday, October 3, 20248:35:21 AM
Multiplier : 1.0000
Dilution : 1.0000
Do not use Multiplier & Dilution Factor with ISTDs

Signal 1: DAD1 A, Sig=320,4 Ref=off

RetTime [min]	Type	Area [mAU*s]	Amt/Area	Amount [ng/ul]	Grp	Name
11.813	BV	390.79935	2.96022e-2	11.56853		Ferulic Acid

Totals : 11.56853

Signal 2: DAD1 B, Sig=280,4 Ref=off

RetTime [min]	Type	Area [mAU*s]	Amt/Area	Amount [ng/ul]	Grp	Name
16.345	BV	33.22510	3.43492e-2	1.14126		Cinnamic Acid

Totals : 1.14126

==

*** End of Report ***

===

Acq. Operator : SYSTEM   Seq. Line : 7
Sample Operator : SYSTEM
Acq. Instrument : LC   Location : P1-A-08
Injection Date : 10/2/2024 12:17:38 PM   Inj : 1
Inj Volume : 5.000 µl
Different Inj Volume from Sample Entry! Actual Inj Volume : 10.000 µl
Acq. Method : C:\Users\Public\Documents\ChemStation\1\Data\R1Phenolics07122024\Kristin Sequence 2024-10-02 09-40-51\Ferulic Acid 300SB C18.M
Last changed : 10/2/2024 9:37:39 AM by SYSTEM
Analysis Method : C:\Users\Public\Documents\ChemStation\1\Data\R1Phenolics07122024\Kristin Sequence 2024-10-02 09-40-51\Ferulic Acid 300SB C18.M (Sequence Method)
Last changed : 10/3/2024 8:35:21 AM by SYSTEM
(modified after loading)

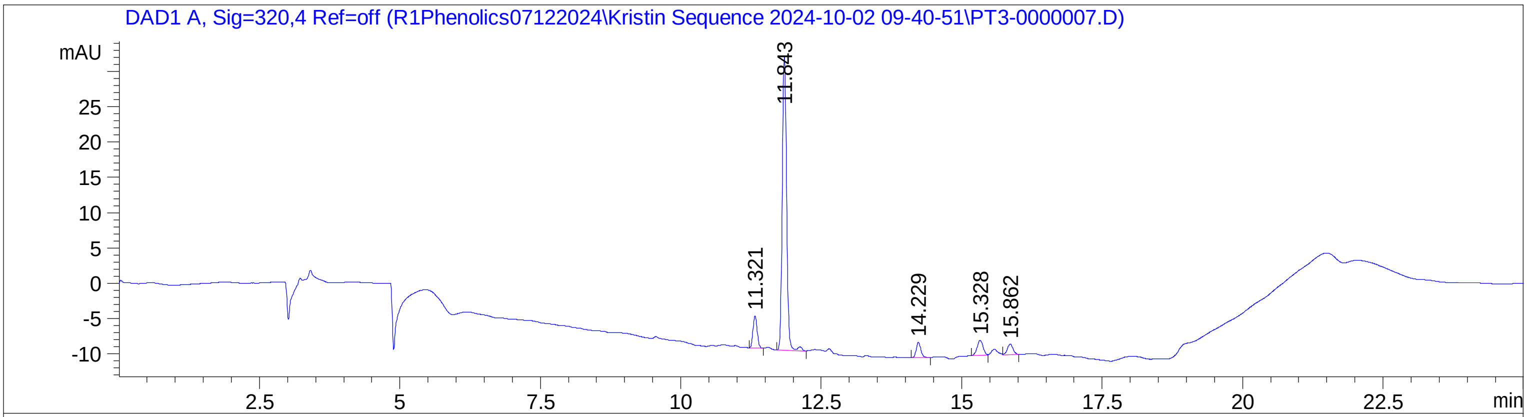

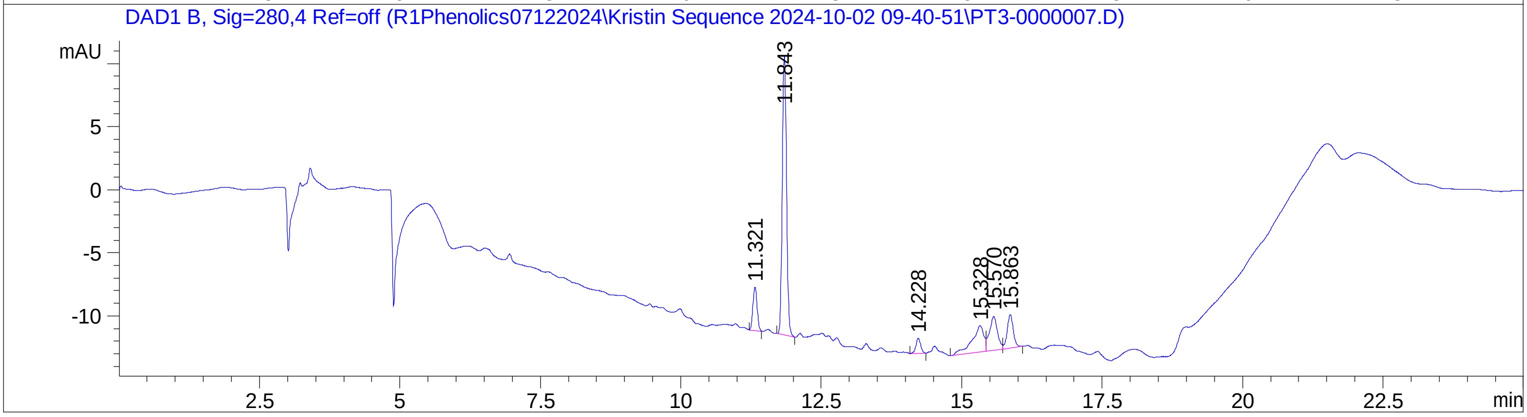

===

Fraction Information

===

No Fractions found.

===

===

===

External Standard Report

===

Sorted By : Signal
Calib. Data Modified : Thursday, October 3, 20248:35:21 AM
Multiplier : 1.0000
Dilution : 1.0000
Do not use Multiplier & Dilution Factor with ISTDs

Signal 1: DAD1 A, Sig=320,4 Ref=off

RetTime [min]	Type	Area [mAU*s]	Amt/Area	Amount [ng/ul]	Grp	Name
11.843	BV R	223.75241	3.01152e-2	6.73836		Ferulic Acid

Totals : 6.73836

Signal 2: DAD1 B, Sig=280,4 Ref=off

RetTime [min]	Type	Area [mAU*s]	Amt/Area	Amount [ng/ul]	Grp	Name
15.863	VB	19.95181	4.49258e-2	8.96350e-1		Cinnamic Acid

Totals : 8.96350e-1

==

*** End of Report ***

===

Acq. Operator : SYSTEM   Seq. Line : 25
Sample Operator : SYSTEM
Acq. Instrument : LC   Location : P1-C-04
Injection Date : 10/2/2024 8:05:13 PM   Inj : 1
                                          Inj Volume : 5.000 µl
Different Inj Volume from Sample Entry! Actual Inj Volume : 10.000 µl
Acq. Method : C:\Users\Public\Documents\ChemStation\1\Data\R1Phenolics07122024\Kristin Sequence 2024-10-02 09-40-51\Ferulic Acid 300SB C18.M
Last changed : 10/2/2024 9:37:39 AM by SYSTEM
Analysis Method : C:\Users\Public\Documents\ChemStation\1\Data\R1Phenolics07122024\Kristin Sequence 2024-10-02 09-40-51\Ferulic Acid 300SB C18.M (Sequence Method)
Last changed : 10/3/2024 8:35:21 AM by SYSTEM
(modified after loading)

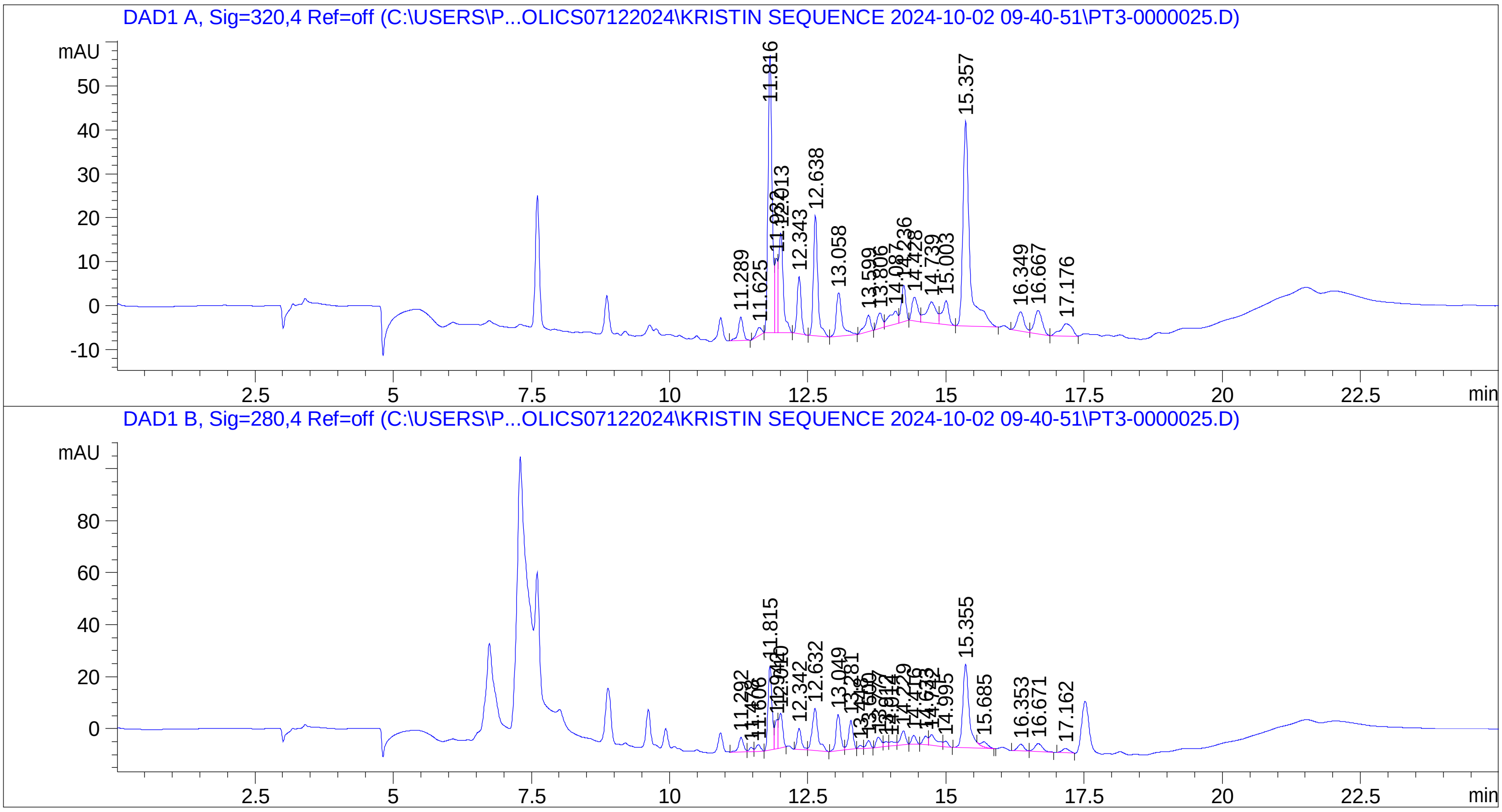

===

Fraction Information

===

No Fractions found.

===

===

===

External Standard Report

===

Sorted By : Signal
Calib. Data Modified : Thursday, October 3, 20248:35:21 AM
Multiplier : 1.0000
Dilution : 1.0000
Do not use Multiplier & Dilution Factor with ISTDs

Signal 1: DAD1 A, Sig=320,4 Ref=off

RetTime [min]	Type	Area [mAU*s]	Amt/Area	Amount [ng/ul]	Grp	Name
11.816	BV	321.77710	2.97496e-2	9.57275		Ferulic Acid

Totals : 9.57275

Signal 2: DAD1 B, Sig=280,4 Ref=off

RetTime [min]	Type	Area [mAU*s]	Amt/Area	Amount [ng/ul]	Grp	Name
16.353	BV	21.41144	4.31210e-2	9.23282e-1		Cinnamic Acid

Totals : 9.23282e-1

==

*** End of Report ***

Data File C:\USERS\P...HENOLICS07122024\KRISTIN SEQUENCE 2024-10-02 09-40-51\PT3-0000026.D
Sample Name: FAX 21

===

Acq. Operator	: SYSTEM	Seq. Line :	26
Sample Operator	: SYSTEM		
Acq. Instrument	: LC	Location :	P1-C-05
Injection Date	: 10/2/2024 8:31:13 PM	Inj :	1
		Inj Volume :	5.000 µl

Different Inj Volume from Sample Entry! Actual Inj Volume : 10.000 µl

Acq. Method : C:\Users\Public\Documents\ChemStation\1\Data\R1Phenolics07122024\Kristin Sequence 2024-10-02 09-40-51\Ferulic Acid 300SB C18.M

Last changed : 10/2/2024 9:37:39 AM by SYSTEM

Analysis Method : C:\Users\Public\Documents\ChemStation\1\Data\R1Phenolics07122024\Kristin Sequence 2024-10-02 09-40-51\Ferulic Acid 300SB C18.M (Sequence Method)

Last changed : 10/3/2024 8:35:21 AM by SYSTEM
(modified after loading)

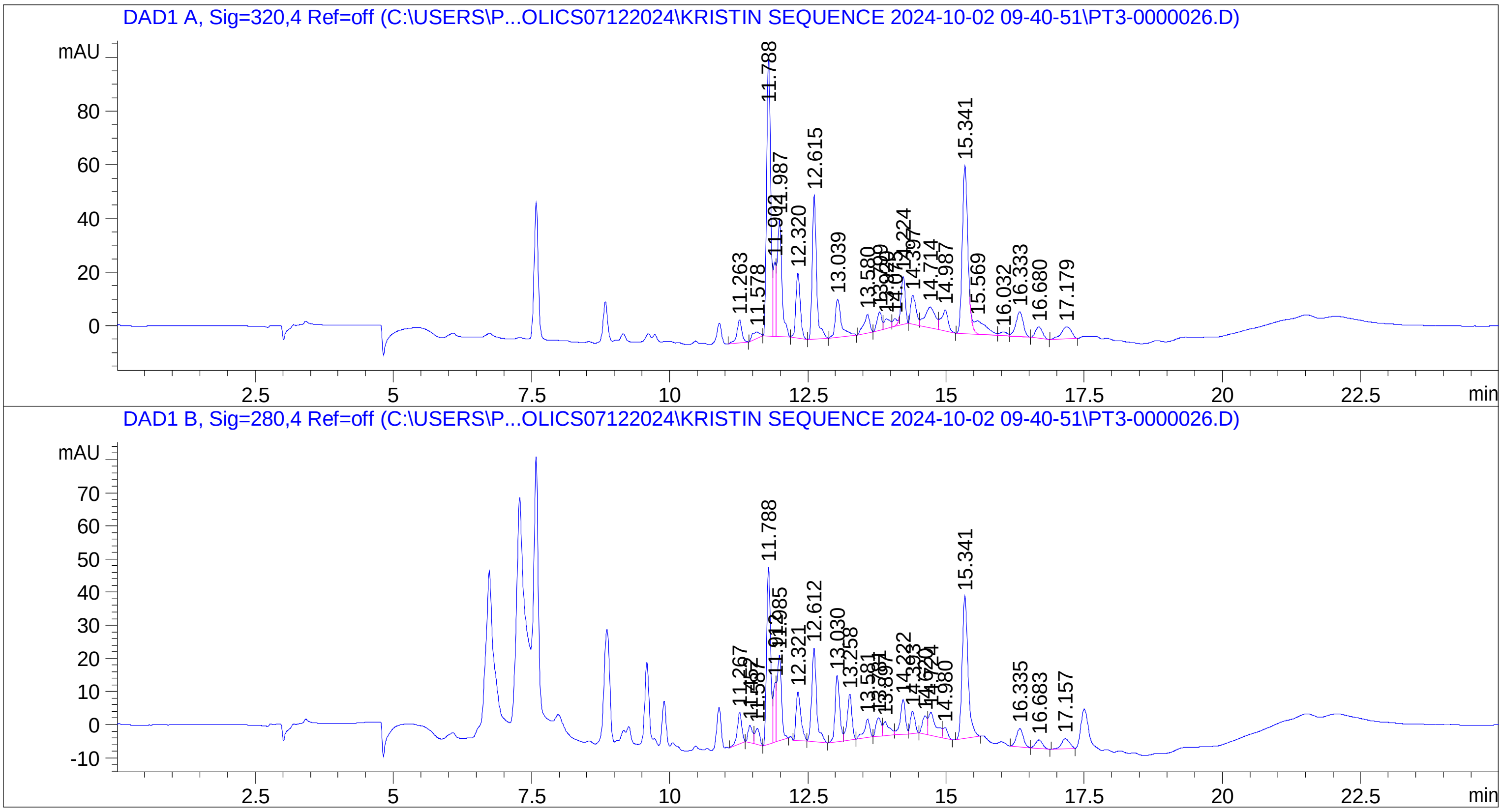

===

Fraction Information

===

No Fractions found.

===

===

===

External Standard Report

===

Sorted By	:	Signal
Calib. Data Modified	:	Thursday, October 3, 20248:35:21 AM
Multiplier	:	1.0000
Dilution	:	1.0000

Do not use Multiplier & Dilution Factor with ISTDs

Signal 1: DAD1 A, Sig=320,4 Ref=off

RetTime [min]	Type	Area [mAU*s]	Amt/Area	Amount [ng/ul]	Grp	Name
11.788	BV	526.31995	2.94253e-2	15.48711		Ferulic Acid

Totals : 15.48711

Signal 2: DAD1 B, Sig=280,4 Ref=off

RetTime [min]	Type	Area [mAU*s]	Amt/Area	Amount [ng/ul]	Grp	Name
16.335	BV	47.92514	2.94728e-2	1.41249		Cinnamic Acid

Totals : 1.41249

*** End of Report ***

===

Acq. Operator	: SYSTEM	Seq. Line :	27
Sample Operator	: SYSTEM		
Acq. Instrument	: LC	Location :	P1-C-06
Injection Date	: 10/2/2024 8:57:12 PM	Inj :	1
		Inj Volume :	5.000 µl

Different Inj Volume from Sample Entry! Actual Inj Volume : 10.000 µl

Acq. Method : C:\Users\Public\Documents\ChemStation\1\Data\R1Phenolics07122024\Kristin Sequence 2024-10-02 09-40-51\Ferulic Acid 300SB C18.M

Last changed : 10/2/2024 9:37:39 AM by SYSTEM

Analysis Method : C:\Users\Public\Documents\ChemStation\1\Data\R1Phenolics07122024\Kristin Sequence 2024-10-02 09-40-51\Ferulic Acid 300SB C18.M (Sequence Method)

Last changed : 10/3/2024 8:35:21 AM by SYSTEM
(modified after loading)

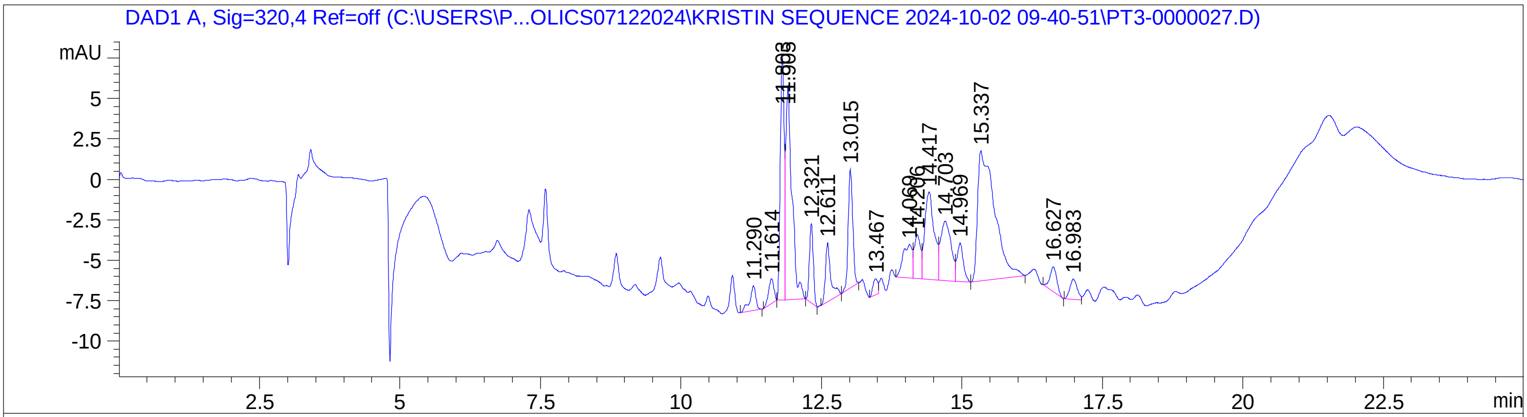

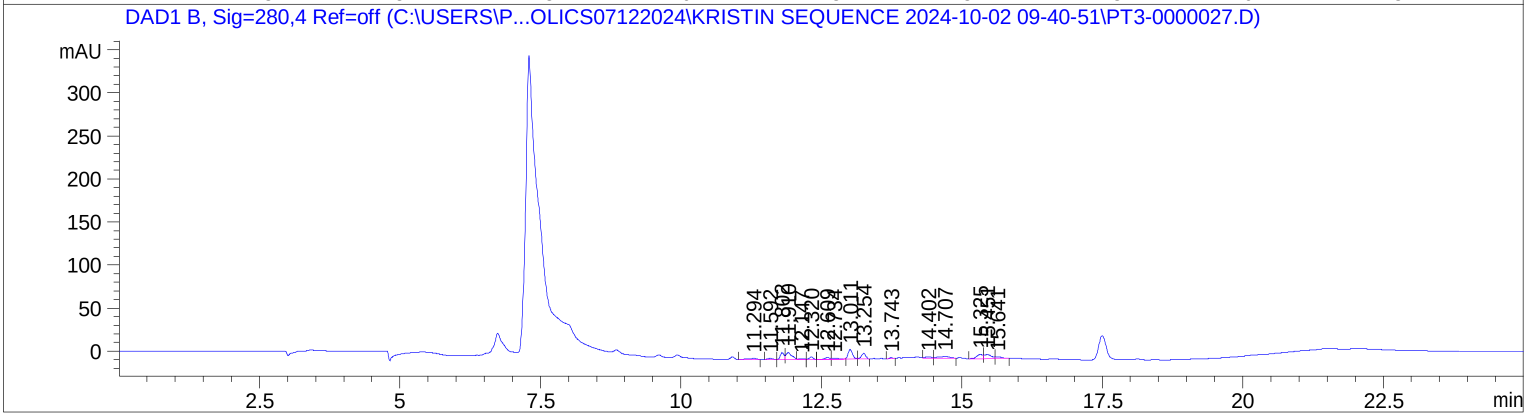

===

Fraction Information

===

No Fractions found.

===

===

===

External Standard Report

===

Sorted By	:	Signal
Calib. Data Modified	:	Thursday, October 3, 20248:35:21 AM
Multiplier	:	1.0000
Dilution	:	1.0000

Do not use Multiplier & Dilution Factor with ISTDs

Signal 1: DAD1 A, Sig=320,4 Ref=off

RetTime [min]	Type	Area [mAU*s]	Amt/Area	Amount [ng/ul]	Grp	Name
11.803	BV	72.90863	3.25984e-2	2.37670		Ferulic Acid

Totals : 2.37670

Signal 2: DAD1 B, Sig=280,4 Ref=off

RetTime [min]	Type	Area [mAU*s]	Amt/Area	Amount [ng/ul]	Grp	Name
16.111		-	-	-		Cinnamic Acid

Totals : 0.00000

1 Warnings or Errors :

Warning : Calibrated compound(s) not found

===

*** End of Report ***

Data File C:\USERS\P...HENOLICS07122024\KRISTIN SEQUENCE 2024-10-02 09-40-51\PT3-0000028.D
Sample Name: FAX 23

===

Acq. Operator : SYSTEM   Seq. Line : 28
Sample Operator : SYSTEM
Acq. Instrument : LC   Location : P1-C-07
Injection Date : 10/2/2024 9:23:11 PM   Inj : 1
Inj Volume : 5.000 µl
Different Inj Volume from Sample Entry! Actual Inj Volume : 10.000 µl
Acq. Method : C:\Users\Public\Documents\ChemStation\1\Data\R1Phenolics07122024\Kristin Sequence 2024-10-02 09-40-51\Ferulic Acid 300SB C18.M
Last changed : 10/2/2024 9:37:39 AM by SYSTEM
Analysis Method : C:\Users\Public\Documents\ChemStation\1\Data\R1Phenolics07122024\Kristin Sequence 2024-10-02 09-40-51\Ferulic Acid 300SB C18.M (Sequence Method)
Last changed : 10/3/2024 8:35:21 AM by SYSTEM
(modified after loading)

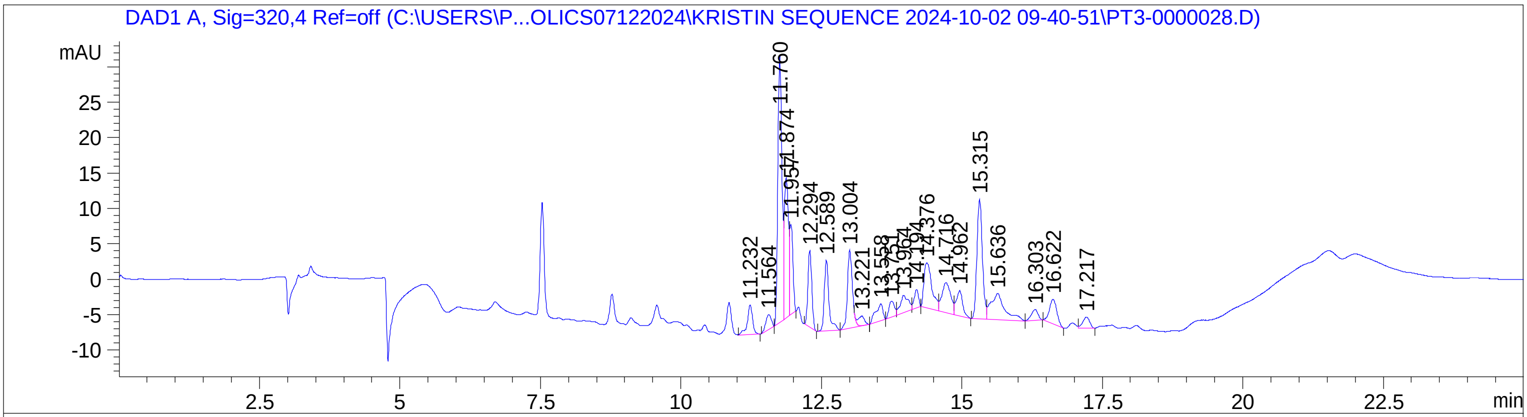

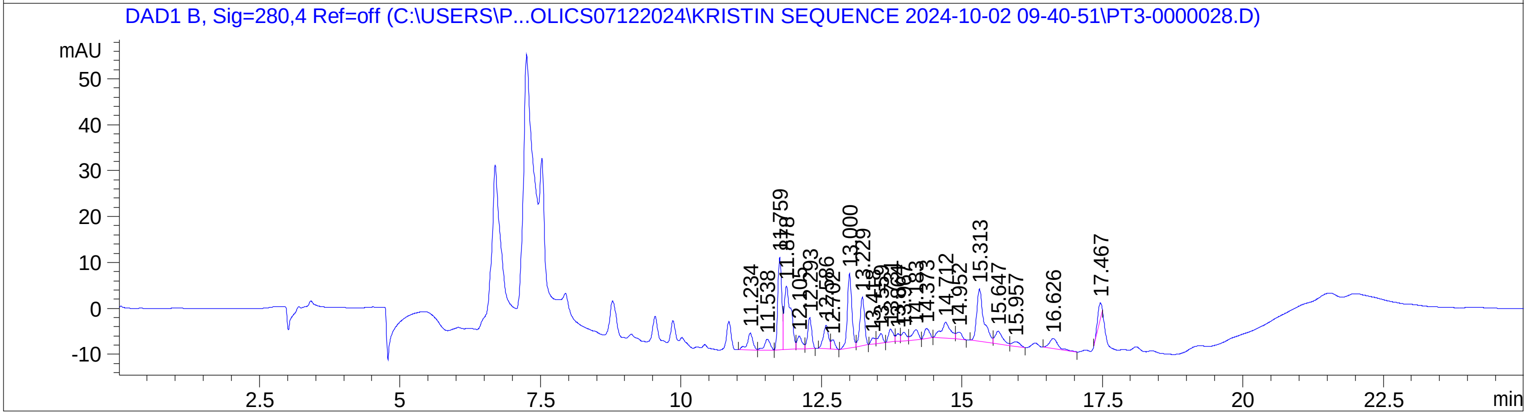

===

Fraction Information

===

No Fractions found.

===

===

===

External Standard Report

===

Sorted By : Signal
Calib. Data Modified : Thursday, October 3, 20248:35:21 AM
Multiplier : 1.0000
Dilution : 1.0000
Do not use Multiplier & Dilution Factor with ISTDs

Signal 1: DAD1 A, Sig=320,4 Ref=off

RetTime [min]	Type	Area [mAU*s]	Amt/Area	Amount [ng/ul]	Grp	Name
11.874	VV	103.81430	3.15019e-2	3.27034		Ferulic Acid

Totals : 3.27034

Signal 2: DAD1 B, Sig=280,4 Ref=off

RetTime [min]	Type	Area [mAU*s]	Amt/Area	Amount [ng/ul]	Grp	Name
15.957	VB	9.59846	7.34827e-2	7.05321e-1		Cinnamic Acid

Totals : 7.05321e-1

===
*** End of Report ***

Data File C:\USERS\P...HENOLICS07122024\KRISTIN SEQUENCE 2024-10-02 09-40-51\PT3-0000029.D
Sample Name: FAX 24

===
Acq. Operator   : SYSTEM   Seq. Line : 29
Sample Operator : SYSTEM
Acq. Instrument : LC   Location :   P1-C-08
Injection Date : 10/2/2024 9:49:10 PM   Inj :   1
                                              Inj Volume : 5.000 µl
Different Inj Volume from Sample Entry! Actual Inj Volume : 10.000 µl
Acq. Method   : C:\Users\Public\Documents\ChemStation\1\Data\R1Phenolics07122024\Kristin Sequence 2024-10-02 09-40-51\Ferulic Acid 300SB C18.M
Last changed   : 10/2/2024 9:37:39 AM by SYSTEM
Analysis Method : C:\Users\Public\Documents\ChemStation\1\Data\R1Phenolics07122024\Kristin Sequence 2024-10-02 09-40-51\Ferulic Acid 300SB C18.M (Sequence Method)
Last changed   : 10/3/2024 8:35:21 AM by SYSTEM
(modified after loading)

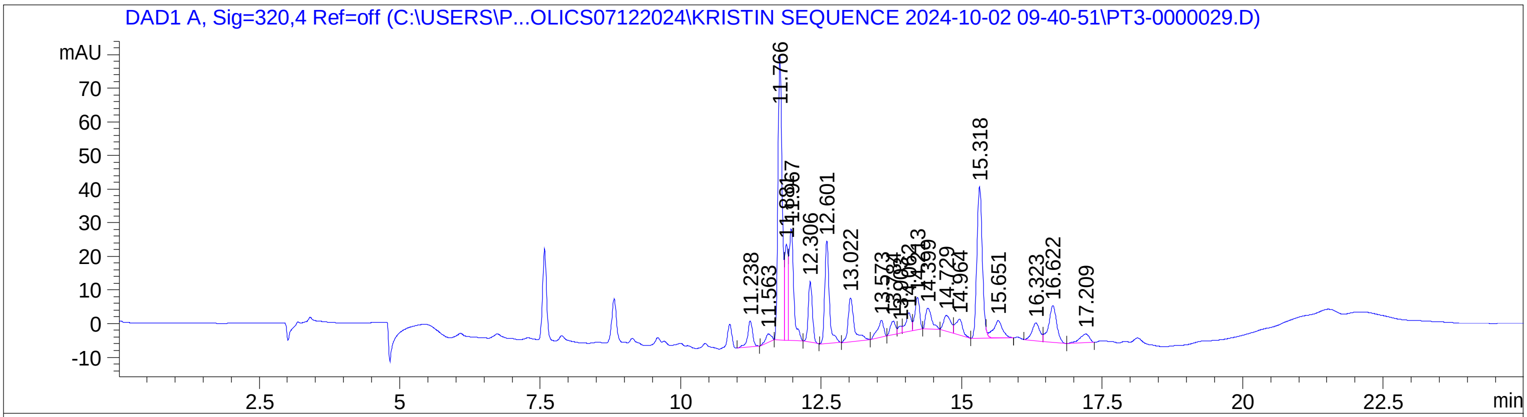

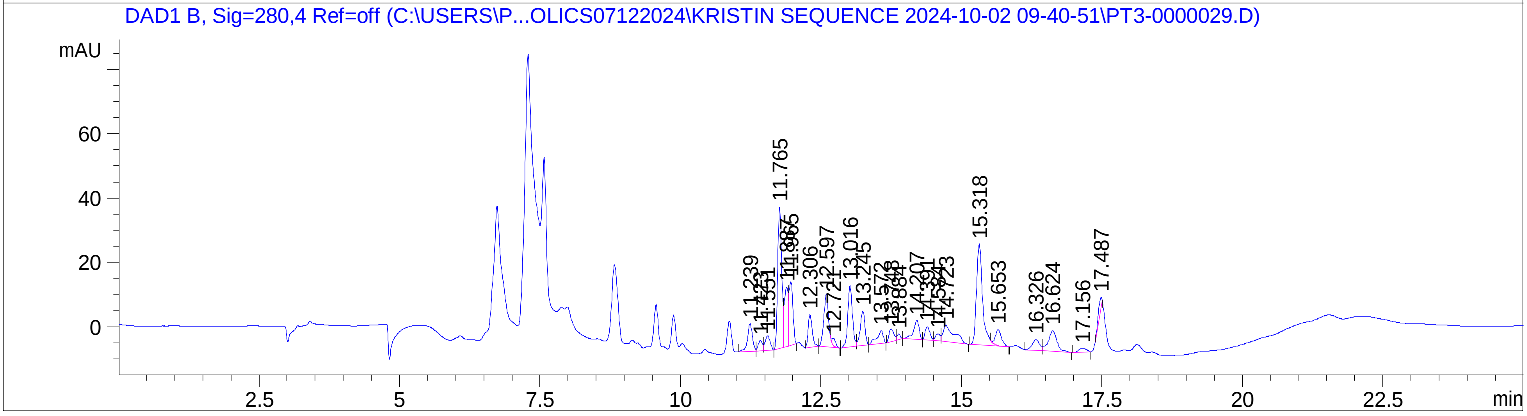

===
Fraction Information
===
No Fractions found.
===
===
===
External Standard Report
===

Sorted By   :   Signal
Calib. Data Modified :   Thursday, October 3, 20248:35:21 AM
Multiplier   :   1.0000
Dilution   :   1.0000
Do not use Multiplier & Dilution Factor with ISTDs

Signal 1: DAD1 A, Sig=320,4 Ref=off

RetTime [min]	Type	Area [mAU*s]	Amt/Area	Amount [ng/ul]	Grp	Name
11.881	VV	122.04630	3.11154e-2	3.79752		Ferulic Acid

Totals : 3.79752

Signal 2: DAD1 B, Sig=280,4 Ref=off

RetTime [min]	Type	Area [mAU*s]	Amt/Area	Amount [ng/ul]	Grp	Name
16.326	BV	31.29363	3.53305e-2	1.10562		Cinnamic Acid

Totals : 1.10562

*** End of Report ***

Data File C:\USERS\P...HENOLICS07122024\KRISTIN SEQUENCE 2024-10-02 09-40-51\PT3-0000030.D
Sample Name: FAX 25

===

Acq. Operator : SYSTEM   Seq. Line : 30
Sample Operator : SYSTEM
Acq. Instrument : LC   Location : P1-C-09
Injection Date : 10/2/2024 10:15:08 PM   Inj : 1
Inj Volume : 5.000 µl
Different Inj Volume from Sample Entry! Actual Inj Volume : 10.000 µl
Acq. Method : C:\Users\Public\Documents\ChemStation\1\Data\R1Phenolics07122024\Kristin Sequence 2024-10-02 09-40-51\Ferulic Acid 300SB C18.M
Last changed : 10/2/2024 9:37:39 AM by SYSTEM
Analysis Method : C:\Users\Public\Documents\ChemStation\1\Data\R1Phenolics07122024\Kristin Sequence 2024-10-02 09-40-51\Ferulic Acid 300SB C18.M (Sequence Method)
Last changed : 10/3/2024 8:35:21 AM by SYSTEM
(modified after loading)

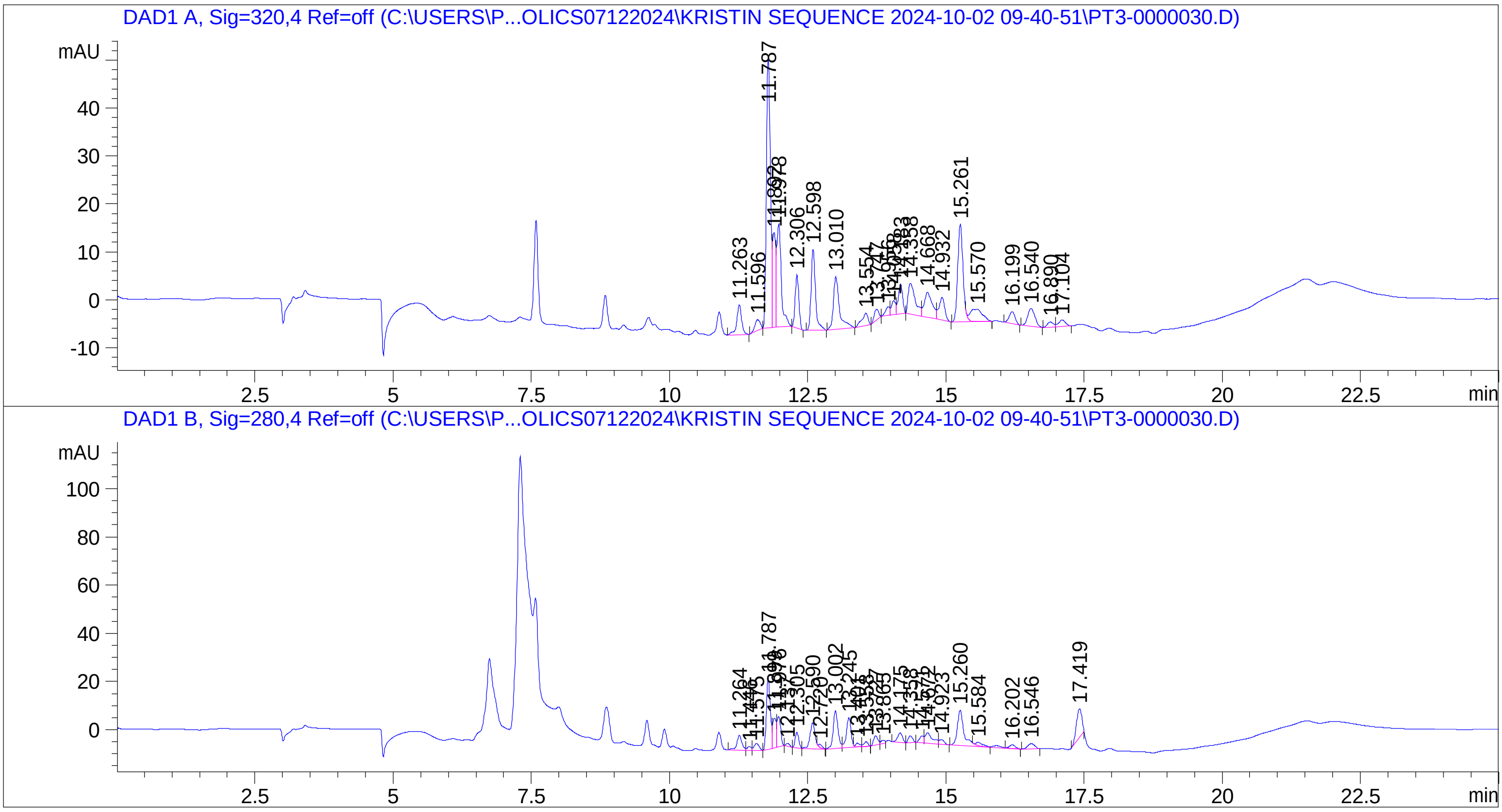

===

Fraction Information

===

No Fractions found.

===

===

===

External Standard Report

===

Sorted By : Signal
Calib. Data Modified : Thursday, October 3, 20248:35:21 AM
Multiplier : 1.0000
Dilution : 1.0000
Do not use Multiplier & Dilution Factor with ISTDs

Signal 1: DAD1 A, Sig=320,4 Ref=off

RetTime [min]	Type	Area [mAU*s]	Amt/Area	Amount [ng/ul]	Grp	Name
11.787	BV	287.37817	2.98495e-2	8.57810		Ferulic Acid

Totals : 8.57810

Signal 2: DAD1 B, Sig=280,4 Ref=off

RetTime [min]	Type	Area [mAU*s]	Amt/Area	Amount [ng/ul]	Grp	Name
16.202	VB E	11.28585	6.52547e-2	7.36455e-1		Cinnamic Acid

Totals : 7.36455e-1

==

*** End of Report ***

Data File C:\Users\P...\FAXr2_10032024\Kristin Sequence 2024-10-03 08-29-56\FAX2-0000007.D
Sample Name: FAX 26

===

Acq. Operator : SYSTEM Seq. Line : 7
Sample Operator : SYSTEM
Acq. Instrument : LC Location : P1-A-07
Injection Date : 10/3/2024 11:06:43 AM Inj : 1
Inj Volume : 5.000 µl
Different Inj Volume from Sample Entry! Actual Inj Volume : 10.000 µl
Acq. Method : C:\Users\Public\Documents\ChemStation\1\Data\FAXr2_10032024\Kristin Sequence 2024-10-03 08-29-56\Ferulic Acid 300SB C18.M
Last changed : 10/2/2024 9:37:39 AM by SYSTEM
Analysis Method : C:\Users\Public\Documents\ChemStation\1\Data\FAXr2_10032024\Kristin Sequence 2024-10-03 08-29-56\Ferulic Acid 300SB C18.M (Sequence Method)
Last changed : 10/4/2024 7:53:25 AM by SYSTEM
(modified after loading)

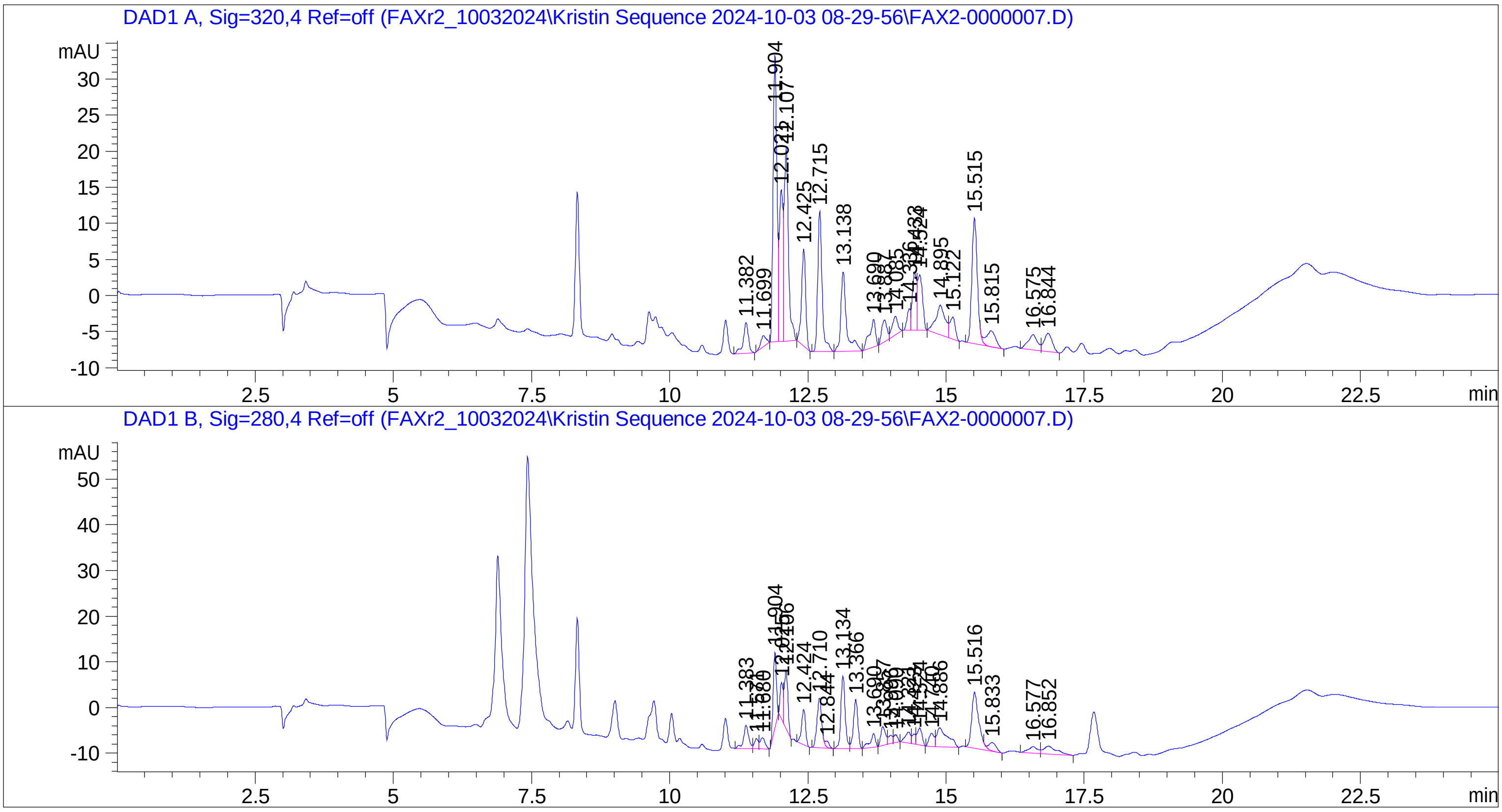

===

Fraction Information

===

No Fractions found.

===

===

===

External Standard Report

===

Sorted By : Signal
Calib. Data Modified : Friday, October 4, 20247:53:25 AM
Multiplier : 1.0000
Dilution : 1.0000
Do not use Multiplier & Dilution Factor with ISTDs

Signal 1: DAD1 A, Sig=320,4 Ref=off

RetTime [min]	Type	Area [mAU*s]	Amt/Area	Amount [ng/ul]	Grp	Name
11.904	BV	194.44701	3.16728e-2	6.15868		Ferulic Acid

Totals : 6.15868

Signal 2: DAD1 B, Sig=280,4 Ref=off

RetTime [min]	Type	Area [mAU*s]	Amt/Area	Amount [ng/ul]	Grp	Name
16.577	BV	16.71219	5.70797e-2	9.53927e-1		Cinnamic Acid

Totals : 9.53927e-1

*** End of Report ***

===

Acq. Operator   : SYSTEM   Seq. Line :   8
Sample Operator : SYSTEM
Acq. Instrument : LC   Location :   P1-A-08
Injection Date : 10/3/2024 11:32:40 AM   Inj :   1
                                              Inj Volume : 5.000 µl
Different Inj Volume from Sample Entry! Actual Inj Volume : 10.000 µl
Acq. Method   : C:\Users\Public\Documents\ChemStation\1\Data\FAXr2_10032024\Kristin Sequence 2024-10-03 08-29-56\Ferulic Acid 300SB C18.M
Last changed   : 10/2/2024 9:37:39 AM by SYSTEM
Analysis Method : C:\Users\Public\Documents\ChemStation\1\Data\FAXr2_10032024\Kristin Sequence 2024-10-03 08-29-56\Ferulic Acid 300SB C18.M (Sequence Method)
Last changed   : 10/4/2024 7:53:25 AM by SYSTEM
                  (modified after loading)

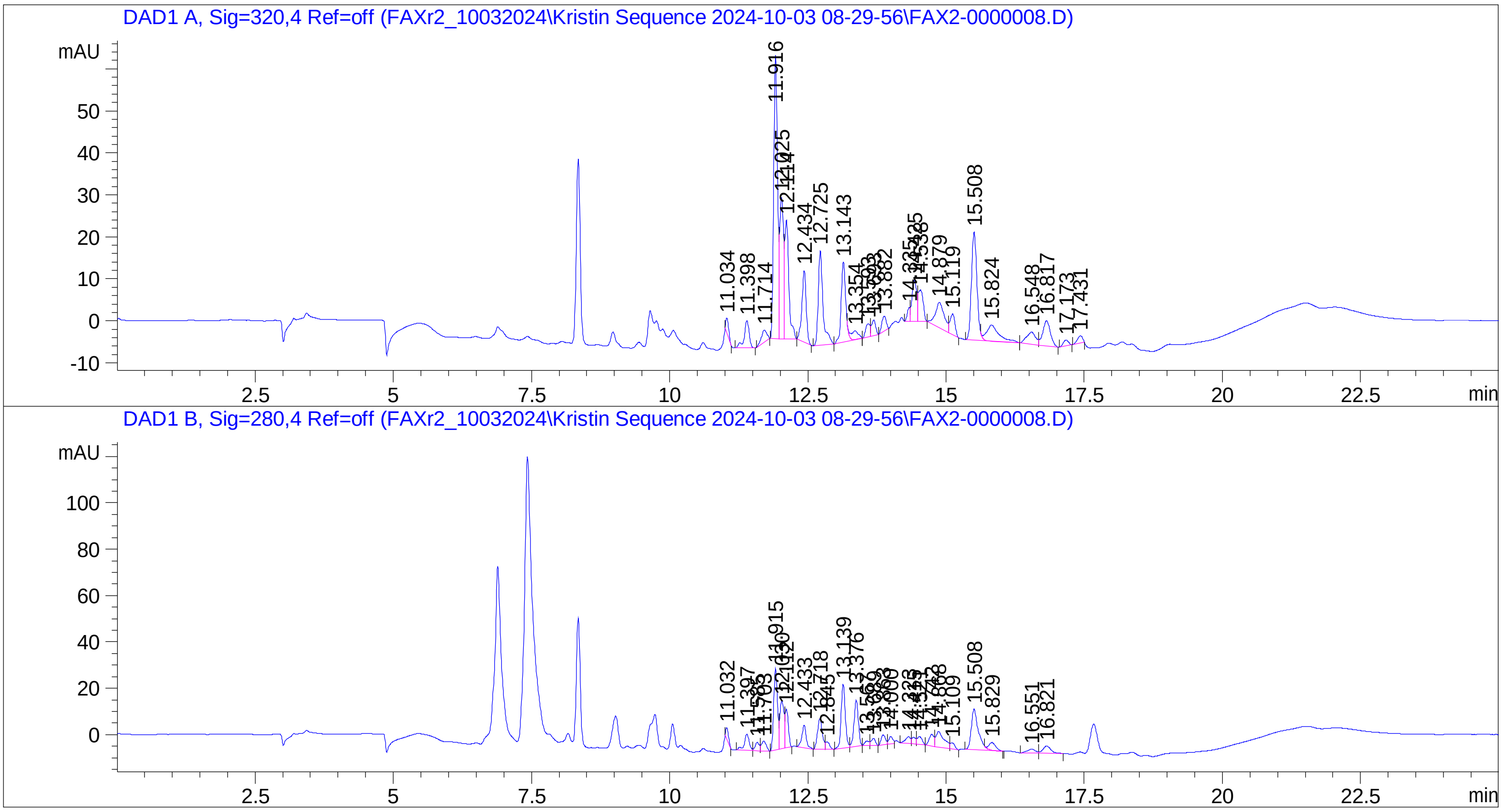

===

Fraction Information

===

No Fractions found.

===

===

===

External Standard Report

===

Sorted By   :   Signal
Calib. Data Modified :   Friday, October 4, 20247:53:25 AM
Multiplier   :   1.0000
Dilution   :   1.0000
Do not use Multiplier & Dilution Factor with ISTDs

Signal 1: DAD1 A, Sig=320,4 Ref=off

RetTime [min]	Type	Area [mAU*s]	Amt/Area	Amount [ng/ul]	Grp	Name
11.916	BV	330.37650	3.02325e-2	9.98809		Ferulic Acid

Totals : 9.98809

Signal 2: DAD1 B, Sig=280,4 Ref=off

RetTime [min]	Type	Area [mAU*s]	Amt/Area	Amount [ng/ul]	Grp	Name
16.551	BV	17.81816	5.46594e-2	9.73930e-1		Cinnamic Acid

Totals : 9.73930e-1

===

*** End of Report ***

===

Acq. Operator : SYSTEM   Seq. Line : 9
Sample Operator : SYSTEM
Acq. Instrument : LC   Location : P1-A-09
Injection Date : 10/3/2024 11:58:39 AM   Inj : 1
Inj Volume : 5.000 µl
Different Inj Volume from Sample Entry! Actual Inj Volume : 10.000 µl
Acq. Method : C:\Users\Public\Documents\ChemStation\1\Data\FAXr2_10032024\Kristin Sequence 2024-10-03 08-29-56\Ferulic Acid 300SB C18.M
Last changed : 10/2/2024 9:37:39 AM by SYSTEM
Analysis Method : C:\Users\Public\Documents\ChemStation\1\Data\FAXr2_10032024\Kristin Sequence 2024-10-03 08-29-56\Ferulic Acid 300SB C18.M (Sequence Method)
Last changed : 10/4/2024 7:53:25 AM by SYSTEM
(modified after loading)

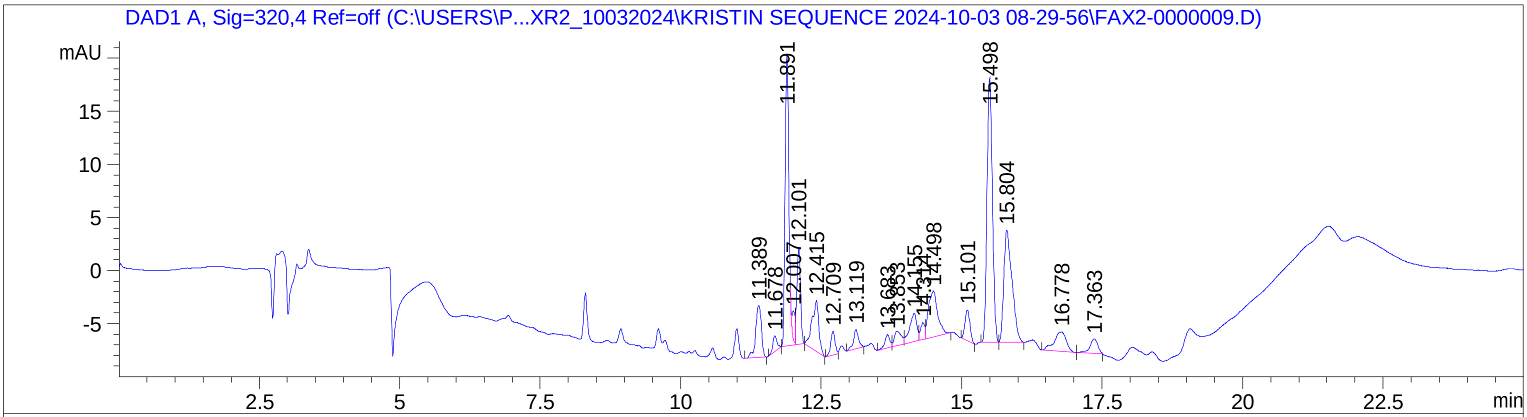

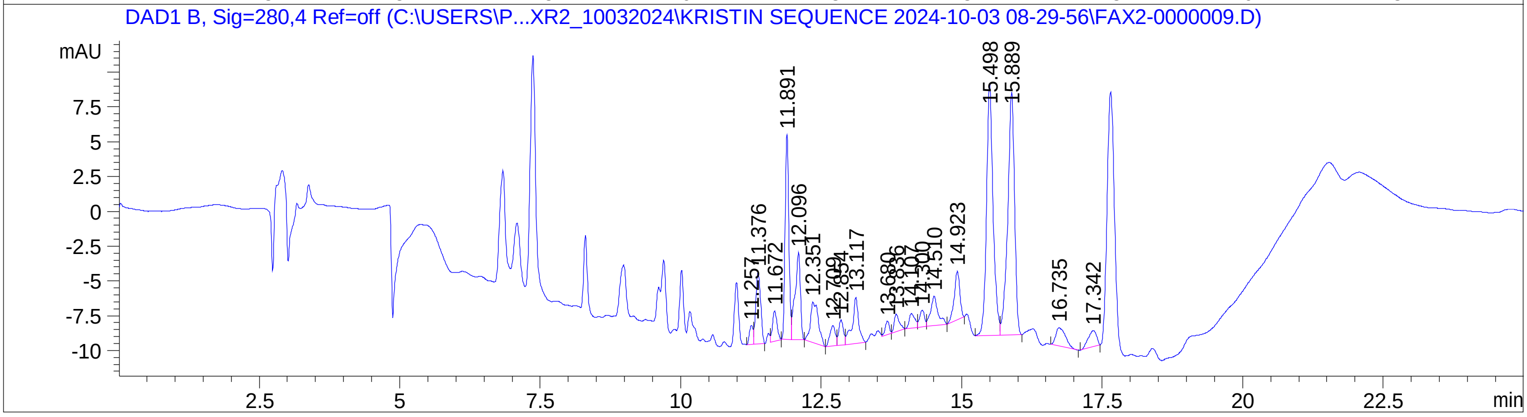

===

Fraction Information

===

No Fractions found.

===

===

===

External Standard Report

===

Sorted By : Signal
Calib. Data Modified : Friday, October 4, 20247:53:25 AM
Multiplier : 1.0000
Dilution : 1.0000
Do not use Multiplier & Dilution Factor with ISTDs

Signal 1: DAD1 A, Sig=320,4 Ref=off

RetTime [min]	Type	Area [mAU*s]	Amt/Area	Amount [ng/ul]	Grp	Name
11.891	BV R	130.13829	3.34028e-2	4.34698		Ferulic Acid

Totals : 4.34698

Signal 2: DAD1 B, Sig=280,4 Ref=off

RetTime [min]	Type	Area [mAU*s]	Amt/Area	Amount [ng/ul]	Grp	Name
15.889	VB	146.98653	2.25202e-2	3.31017		Cinnamic Acid

Totals : 3.31017

===

*** End of Report ***

Data File C:\Users\P...\FAXr2_10032024\Kristin Sequence 2024-10-03 08-29-56\FAX2-0000010.D
Sample Name: FAX 29

===

Acq. Operator	: SYSTEM	Seq. Line :	10
Sample Operator	: SYSTEM		
Acq. Instrument	: LC	Location :	P1-A-10
Injection Date	: 10/3/2024 12:24:38 PM	Inj :	1
		Inj Volume :	5.000 µl

Different Inj Volume from Sample Entry! Actual Inj Volume : 10.000 µl

Acq. Method : C:\Users\Public\Documents\ChemStation\1\Data\FAXr2_10032024\Kristin Sequence 2024-10-03 08-29-56\Ferulic Acid 300SB C18.M

Last changed : 10/2/2024 9:37:39 AM by SYSTEM

Analysis Method : C:\Users\Public\Documents\ChemStation\1\Data\FAXr2_10032024\Kristin Sequence 2024-10-03 08-29-56\Ferulic Acid 300SB C18.M (Sequence Method)

Last changed : 10/4/2024 7:53:25 AM by SYSTEM (modified after loading)

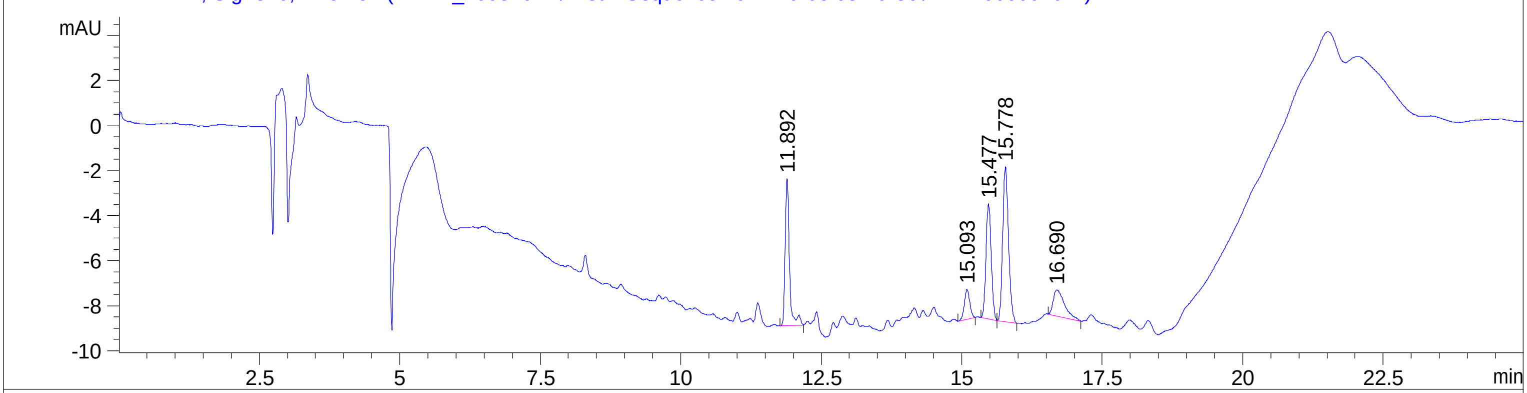

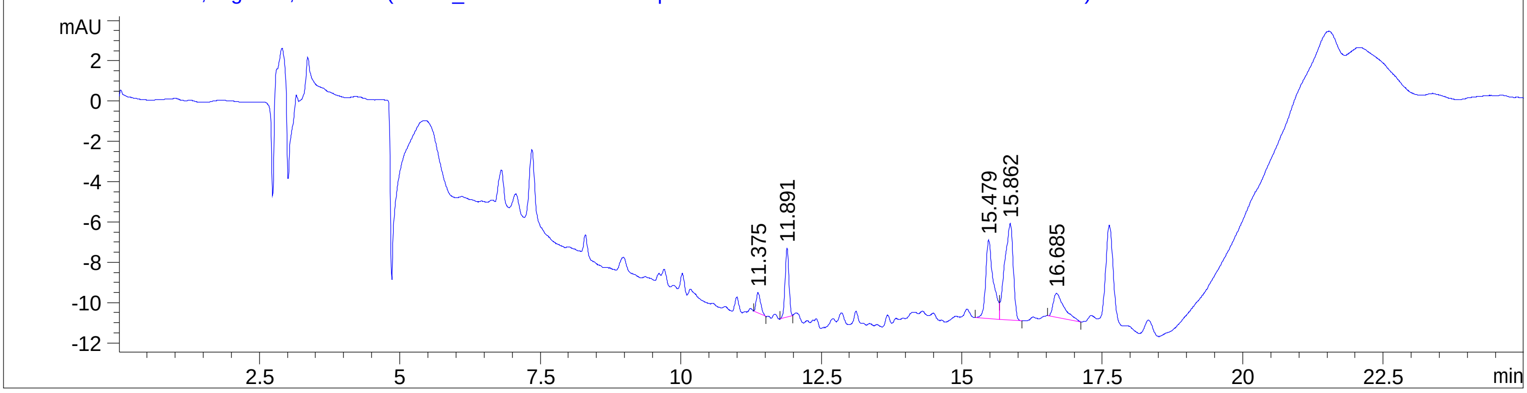

===

Fraction Information

===

No Fractions found.

===

===

External Standard Report

===

Sorted By	:	Signal
Calib. Data Modified	:	Friday, October 4, 20247:53:25 AM
Multiplier	:	1.0000
Dilution	:	1.0000

Do not use Multiplier & Dilution Factor with ISTDs

Signal 1: DAD1 A, Sig=320,4 Ref=off

RetTime [min]	Type	Area [mAU*s]	Amt/Area	Amount [ng/ul]	Grp	Name
11.892	BV R	34.92405	4.76635e-2	1.66460		Ferulic Acid

Totals : 1.66460

Signal 2: DAD1 B, Sig=280,4 Ref=off

RetTime [min]	Type	Area [mAU*s]	Amt/Area	Amount [ng/ul]	Grp	Name
15.862	VB	50.27052	3.10498e-2	1.56089		Cinnamic Acid

Totals : 1.56089

*** End of Report ***

===

Acq. Operator   : SYSTEM   Seq. Line :   8
Sample Operator : SYSTEM
Acq. Instrument : LC   Location :   P1-A-09
Injection Date : 10/2/2024 12:43:37 PM   Inj :   1
                                               Inj Volume : 5.000 µl
Different Inj Volume from Sample Entry! Actual Inj Volume : 10.000 µl
Acq. Method   : C:\Users\Public\Documents\ChemStation\1\Data\R1Phenolics07122024\Kristin Sequence 2024-10-02 09-40-51\Ferulic Acid 300SB C18.M
Last changed   : 10/2/2024 9:37:39 AM by SYSTEM
Analysis Method : C:\Users\Public\Documents\ChemStation\1\Data\R1Phenolics07122024\Kristin Sequence 2024-10-02 09-40-51\Ferulic Acid 300SB C18.M (Sequence Method)
Last changed   : 10/3/2024 8:35:21 AM by SYSTEM
                  (modified after loading)

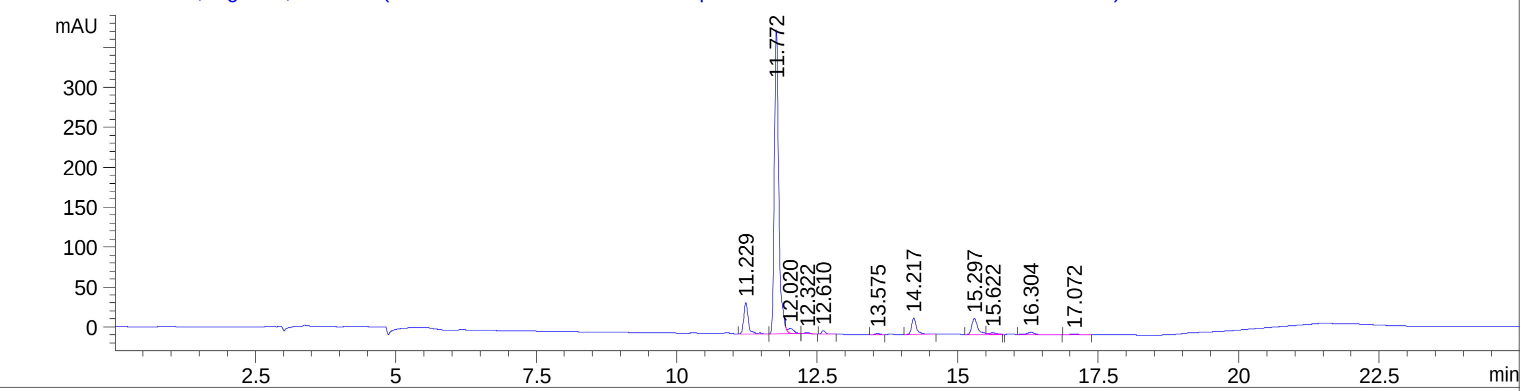

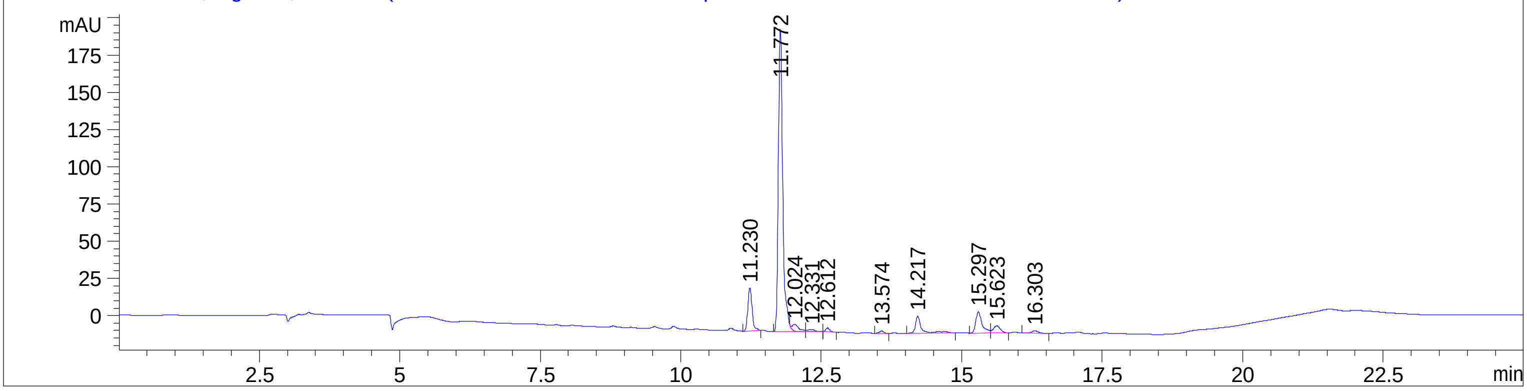

===

Fraction Information

===

No Fractions found.

===

===

===

External Standard Report

===

Sorted By   :   Signal
Calib. Data Modified :   Thursday, October 3, 20248:35:21 AM
Multiplier   :   1.0000
Dilution   :   1.0000
Do not use Multiplier & Dilution Factor with ISTDs

Signal 1: DAD1 A, Sig=320,4 Ref=off

RetTime [min]	Type	Area [mAU*s]	Amt/Area	Amount [ng/ul]	Grp	Name
11.772	BV R	2052.20557	2.90459e-2	59.60815		Ferulic Acid

Totals : 59.60815

Signal 2: DAD1 B, Sig=280,4 Ref=off

RetTime [min]	Type	Area [mAU*s]	Amt/Area	Amount [ng/ul]	Grp	Name
16.303	BB	14.15796	5.57600e-2	7.89448e-1		Cinnamic Acid

Totals : 7.89448e-1

*** End of Report ***

===

Acq. Operator : SYSTEM Seq. Line : 11
Sample Operator : SYSTEM
Acq. Instrument : LC Location : P1-A-11
Injection Date : 10/3/2024 12:50:37 PM Inj : 1
Inj Volume : 5.000 µl
Different Inj Volume from Sample Entry! Actual Inj Volume : 10.000 µl
Acq. Method : C:\Users\Public\Documents\ChemStation\1\Data\FAXr2_10032024\Kristin Sequence 2024-10-03 08-29-56\Ferulic Acid 300SB C18.M
Last changed : 10/2/2024 9:37:39 AM by SYSTEM
Analysis Method : C:\Users\Public\Documents\ChemStation\1\Data\FAXr2_10032024\Kristin Sequence 2024-10-03 08-29-56\Ferulic Acid 300SB C18.M (Sequence Method)
Last changed : 10/4/2024 7:53:25 AM by SYSTEM
(modified after loading)

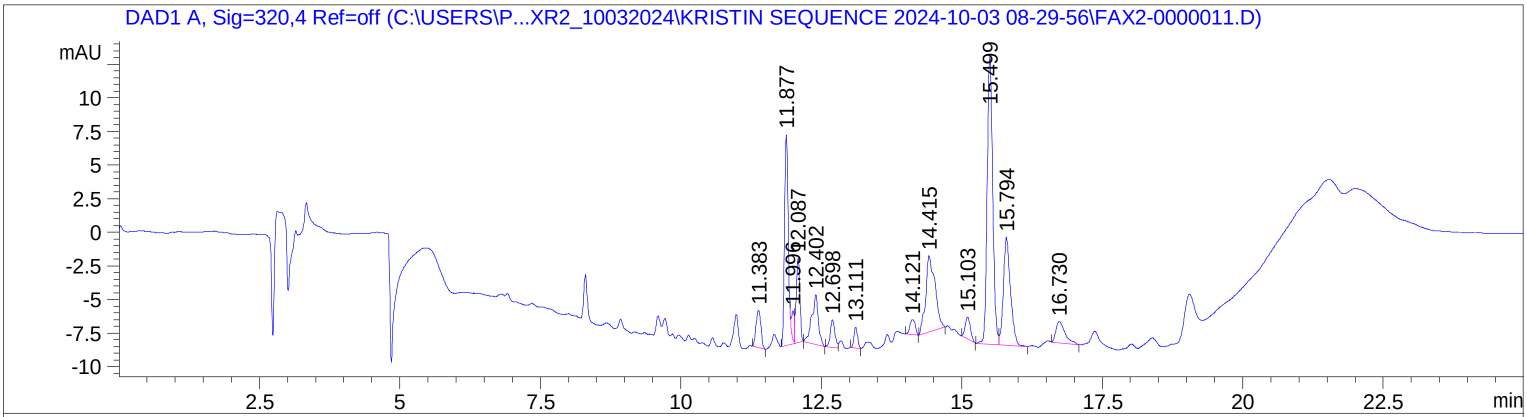

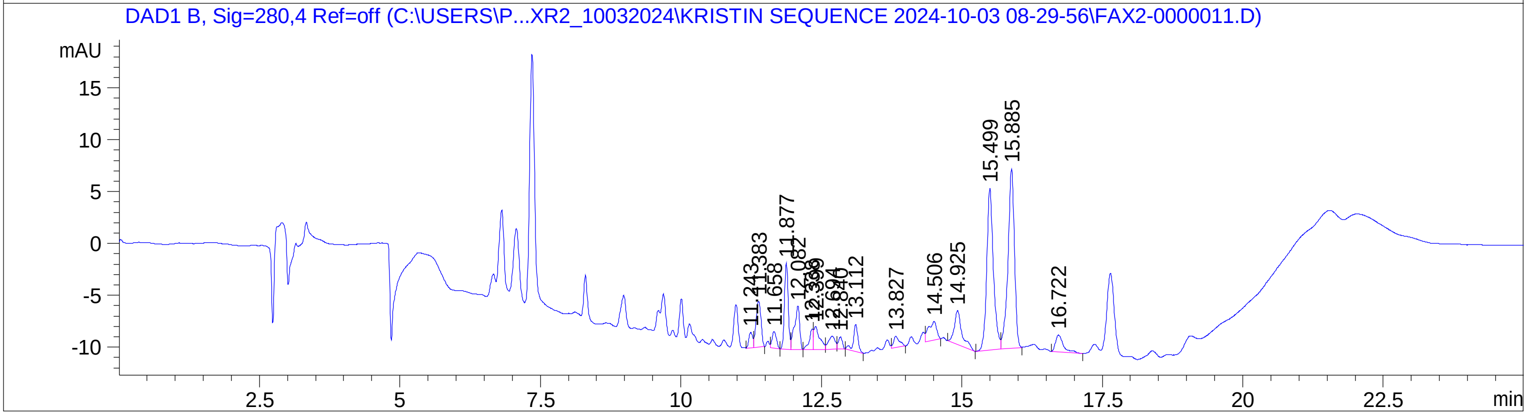

===

Fraction Information

===

No Fractions found.

===

===

===

External Standard Report

===

Sorted By : Signal
Calib. Data Modified : Friday, October 4, 20247:53:25 AM
Multiplier : 1.0000
Dilution : 1.0000
Do not use Multiplier & Dilution Factor with ISTDs

Signal 1: DAD1 A, Sig=320,4 Ref=off

RetTime [min]	Type	Area [mAU*s]	Amt/Area	Amount [ng/ul]	Grp	Name
11.877	BV R	74.96730	3.72523e-2	2.79270		Ferulic Acid

Totals : 2.79270

Signal 2: DAD1 B, Sig=280,4 Ref=off

RetTime [min]	Type	Area [mAU*s]	Amt/Area	Amount [ng/ul]	Grp	Name
15.885	VB	139.42288	2.27608e-2	3.17337		Cinnamic Acid

Totals : 3.17337

===

*** End of Report ***

Data File C:\USERS\P...\FAXR2_10032024\KRISTIN SEQUENCE 2024-10-03 08-29-56\FAX2-0000012.D
Sample Name: FAX 31

===
Acq. Operator   : SYSTEM   Seq. Line : 12
Sample Operator : SYSTEM
Acq. Instrument : LC   Location :   P1-B-01
Injection Date : 10/3/2024 1:16:36 PM   Inj :   1
                                                 Inj Volume : 5.000 µl
Different Inj Volume from Sample Entry! Actual Inj Volume : 10.000 µl
Acq. Method   : C:\Users\Public\Documents\ChemStation\1\Data\FAXr2_10032024\Kristin Sequence 2024-10-03 08-29-56\Ferulic Acid 300SB C18.M
Last changed   : 10/2/2024 9:37:39 AM by SYSTEM
Analysis Method : C:\Users\Public\Documents\ChemStation\1\Data\FAXr2_10032024\Kristin Sequence 2024-10-03 08-29-56\Ferulic Acid 300SB C18.M (Sequence Method)
Last changed   : 10/4/2024 7:53:25 AM by SYSTEM
                  (modified after loading)

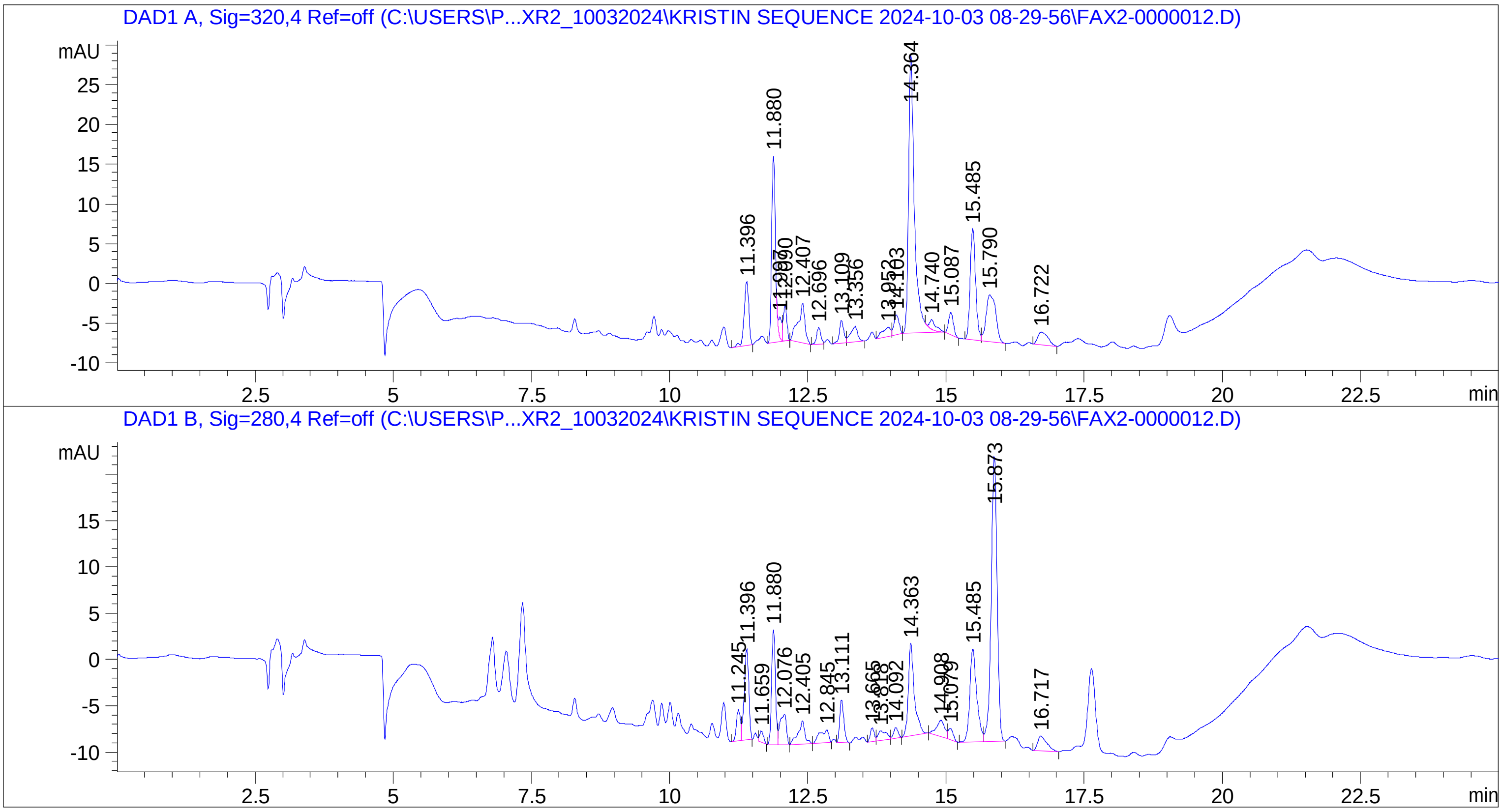

===
Fraction Information
===
No Fractions found.
===
===
===
External Standard Report
===

Sorted By   :   Signal
Calib. Data Modified :   Friday, October 4, 20247:53:25 AM
Multiplier   :   1.0000
Dilution   :   1.0000
Do not use Multiplier & Dilution Factor with ISTDs

Signal 1: DAD1 A, Sig=320,4 Ref=off

RetTime [min]	Type	Area [mAU*s]	Amt/Area	Amount [ng/ul]	Grp	Name
11.880	BV R	115.80354	3.40503e-2	3.94314		Ferulic Acid

Totals : 3.94314

Signal 2: DAD1 B, Sig=280,4 Ref=off

RetTime [min]	Type	Area [mAU*s]	Amt/Area	Amount [ng/ul]	Grp	Name
15.873	VB	228.70163	2.09362e-2	4.78814		Cinnamic Acid

Totals : 4.78814

===

*** End of Report ***

===

Acq. Operator : SYSTEM Seq. Line : 13
Sample Operator : SYSTEM
Acq. Instrument : LC Location : P1-B-02
Injection Date : 10/3/2024 1:42:35 PM Inj : 1
Inj Volume : 5.000 µl
Different Inj Volume from Sample Entry! Actual Inj Volume : 10.000 µl
Acq. Method : C:\Users\Public\Documents\ChemStation\1\Data\FAXr2_10032024\Kristin Sequence 2024-10-03 08-29-56\Ferulic Acid 300SB C18.M
Last changed : 10/2/2024 9:37:39 AM by SYSTEM
Analysis Method : C:\Users\Public\Documents\ChemStation\1\Data\FAXr2_10032024\Kristin Sequence 2024-10-03 08-29-56\Ferulic Acid 300SB C18.M (Sequence Method)
Last changed : 10/4/2024 7:53:25 AM by SYSTEM
(modified after loading)

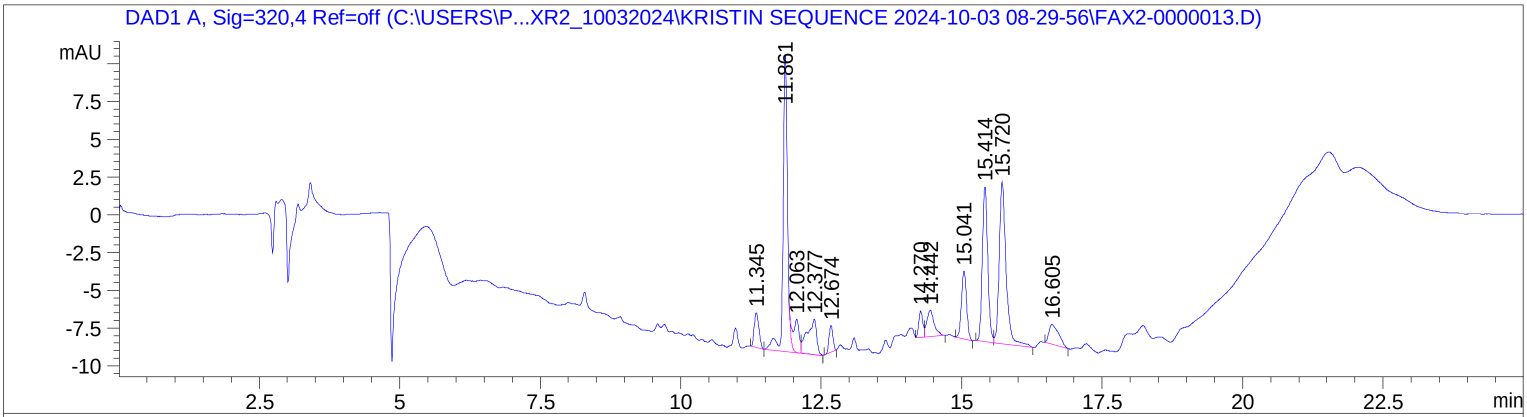

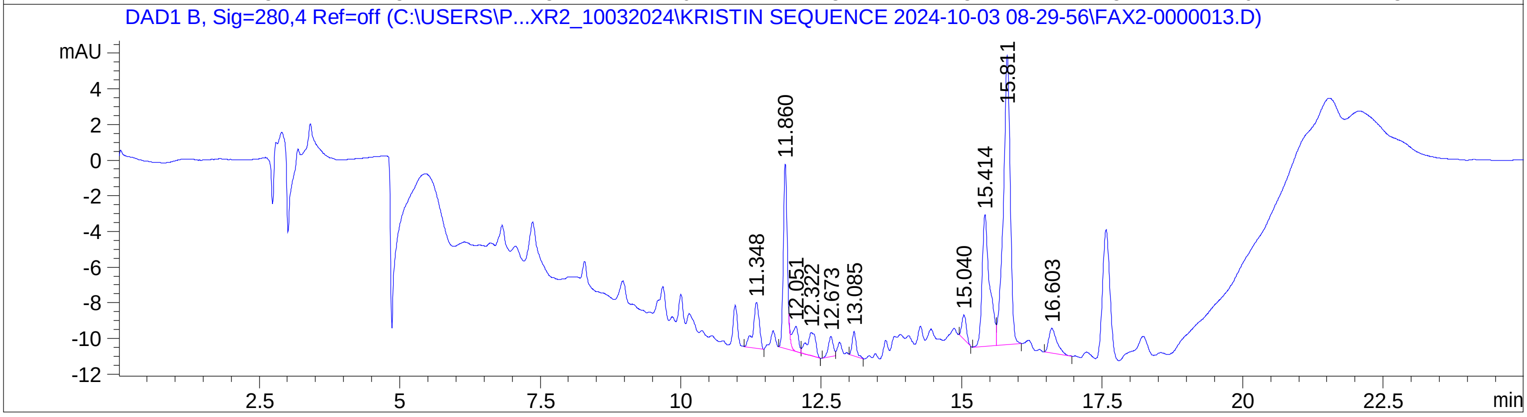

===

Fraction Information

===

No Fractions found.

===

===

===

External Standard Report

===

Sorted By : Signal
Calib. Data Modified : Friday, October 4, 20247:53:25 AM
Multiplier : 1.0000
Dilution : 1.0000
Do not use Multiplier & Dilution Factor with ISTDs

Signal 1: DAD1 A, Sig=320,4 Ref=off

RetTime [min]	Type	Area [mAU*s]	Amt/Area	Amount [ng/ul]	Grp	Name
11.861	VV R	107.67839	3.44938e-2	3.71424		Ferulic Acid

Totals : 3.71424

Signal 2: DAD1 B, Sig=280,4 Ref=off

RetTime [min]	Type	Area [mAU*s]	Amt/Area	Amount [ng/ul]	Grp	Name
16.603	BB	13.99603	6.46469e-2	9.04800e-1		Cinnamic Acid

Totals : 9.04800e-1

==

*** End of Report ***

===

```
Acq. Operator   : SYSTEM                        Seq. Line :  14
Sample Operator : SYSTEM
Acq. Instrument : LC                             Location :   P1-B-03
Injection Date  : 10/3/2024 2:08:32 PM                Inj :   1
                                               Inj Volume : 5.000 µl
Different Inj Volume from Sample Entry!  Actual Inj Volume : 10.000 µl
Acq. Method     : C:\Users\Public\Documents\ChemStation\1\Data\FAXr2_10032024\Kristin
                  Sequence 2024-10-03 08-29-56\Ferulic Acid 300SB C18.M
Last changed    : 10/2/2024 9:37:39 AM by SYSTEM
Analysis Method : C:\Users\Public\Documents\ChemStation\1\Data\FAXr2_10032024\Kristin
                  Sequence 2024-10-03 08-29-56\Ferulic Acid 300SB C18.M (Sequence Method)
Last changed    : 10/4/2024 7:53:25 AM by SYSTEM
                  (modified after loading)
```

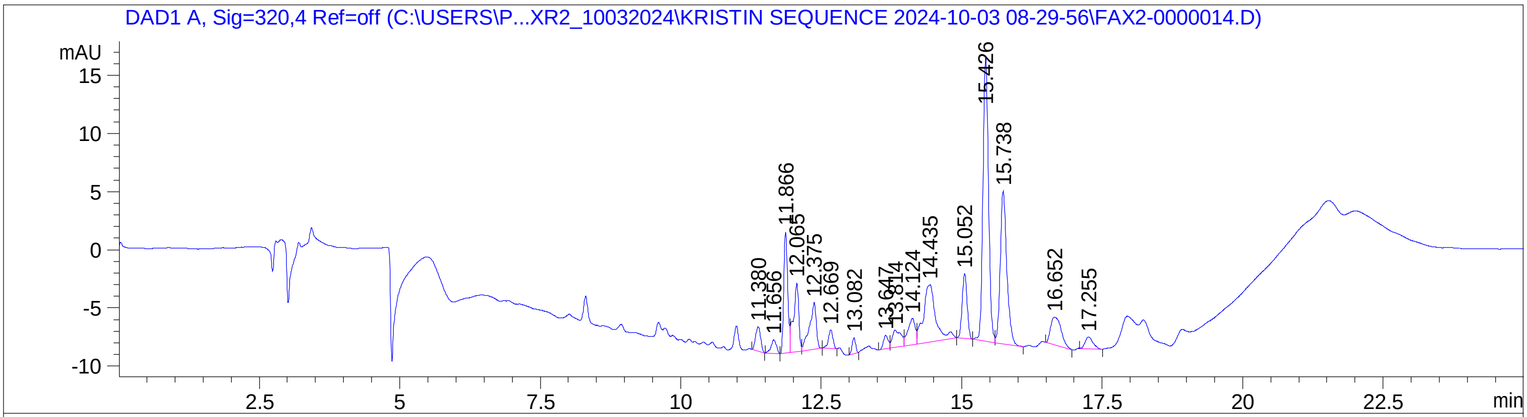

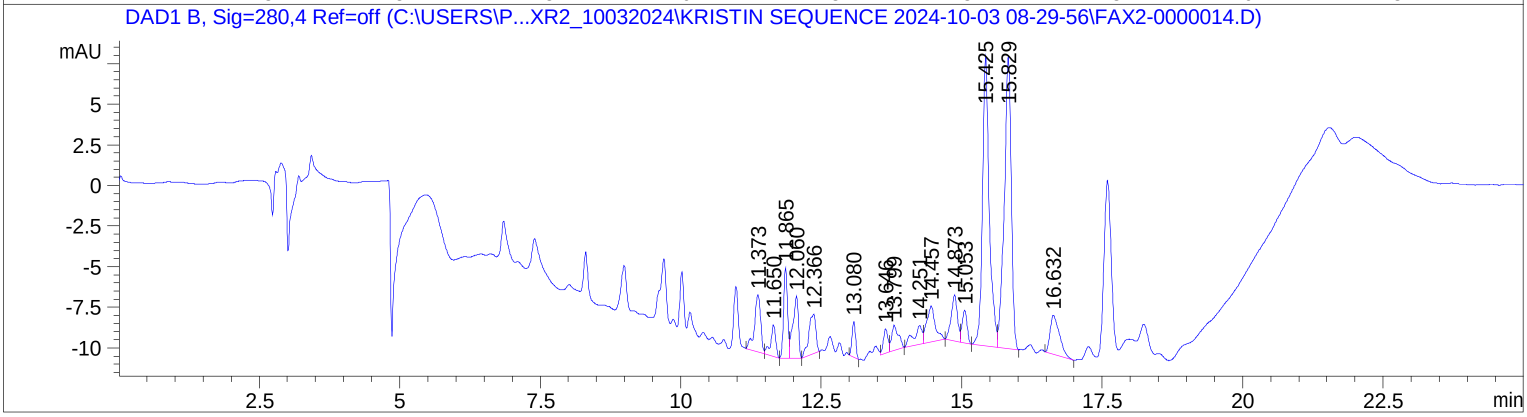

===

Fraction Information

===

No Fractions found.

===

===

===

External Standard Report

===

```
Sorted By             :      Signal
Calib. Data Modified  :      Friday, October 4, 20247:53:25 AM
Multiplier            :      1.0000
Dilution              :      1.0000
Do not use Multiplier & Dilution Factor with ISTDs
```

Signal 1: DAD1 A, Sig=320,4 Ref=off

RetTime [min]	Type	Area [mAU*s]	Amt/Area	Amount [ng/ul]	Grp	Name
11.866	BV	50.83940	4.15616e-2	2.11297		Ferulic Acid

Totals : 2.11297

Signal 2: DAD1 B, Sig=280,4 Ref=off

RetTime [min]	Type	Area [mAU*s]	Amt/Area	Amount [ng/ul]	Grp	Name
16.632	BB	27.69842	4.16136e-2	1.15263		Cinnamic Acid

Totals : 1.15263

*** End of Report ***

===

Acq. Operator : SYSTEM   Seq. Line : 15
Sample Operator : SYSTEM
Acq. Instrument : LC   Location : P1-B-04
Injection Date : 10/3/2024 2:34:31 PM   Inj : 1
Inj Volume : 5.000 µl
Different Inj Volume from Sample Entry! Actual Inj Volume : 10.000 µl
Acq. Method : C:\Users\Public\Documents\ChemStation\1\Data\FAXr2_10032024\Kristin Sequence 2024-10-03 08-29-56\Ferulic Acid 300SB C18.M
Last changed : 10/2/2024 9:37:39 AM by SYSTEM
Analysis Method : C:\Users\Public\Documents\ChemStation\1\Data\FAXr2_10032024\Kristin Sequence 2024-10-03 08-29-56\Ferulic Acid 300SB C18.M (Sequence Method)
Last changed : 10/4/2024 7:53:25 AM by SYSTEM
(modified after loading)

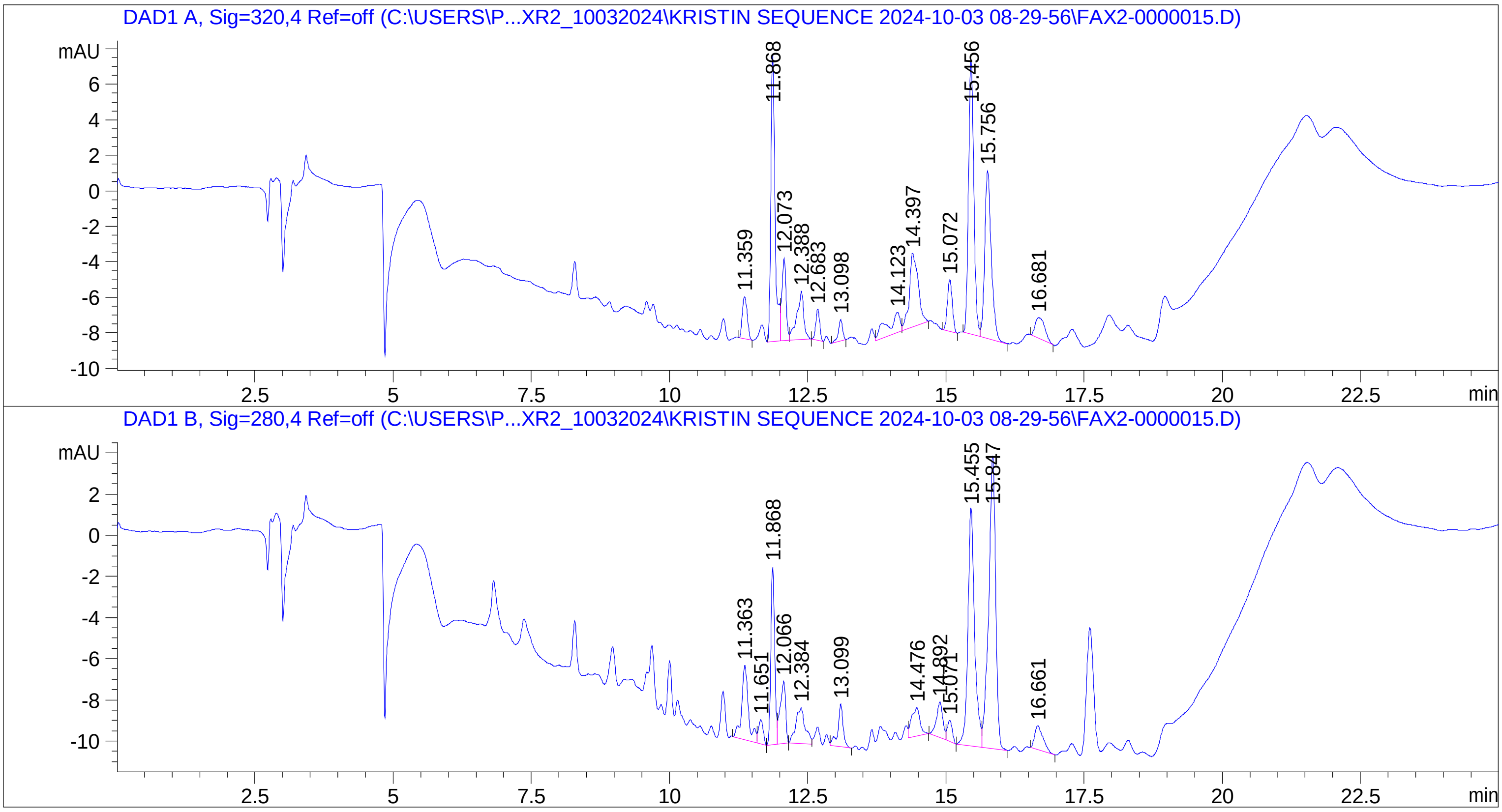

===

Fraction Information

===

No Fractions found.

===

===

===

External Standard Report

===

Sorted By : Signal
Calib. Data Modified : Friday, October 4, 20247:53:25 AM
Multiplier : 1.0000
Dilution : 1.0000
Do not use Multiplier & Dilution Factor with ISTDs

Signal 1: DAD1 A, Sig=320,4 Ref=off

RetTime [min]	Type	Area [mAU*s]	Amt/Area	Amount [ng/ul]	Grp	Name
11.868	BV	85.36452	3.61463e-2	3.08561		Ferulic Acid

Totals : 3.08561

Signal 2: DAD1 B, Sig=280,4 Ref=off

RetTime [min]	Type	Area [mAU*s]	Amt/Area	Amount [ng/ul]	Grp	Name
15.847	VB	123.99701	2.33422e-2	2.89436		Cinnamic Acid

Totals : 2.89436

===

*** End of Report ***

===

Acq. Operator : SYSTEM   Seq. Line : 16
Sample Operator : SYSTEM
Acq. Instrument : LC   Location : P1-B-05
Injection Date : 10/3/2024 3:00:29 PM   Inj : 1
Inj Volume : 5.000 µl
Different Inj Volume from Sample Entry! Actual Inj Volume : 10.000 µl
Acq. Method : C:\Users\Public\Documents\ChemStation\1\Data\FAXr2_10032024\Kristin Sequence 2024-10-03 08-29-56\Ferulic Acid 300SB C18.M
Last changed : 10/2/2024 9:37:39 AM by SYSTEM
Analysis Method : C:\Users\Public\Documents\ChemStation\1\Data\FAXr2_10032024\Kristin Sequence 2024-10-03 08-29-56\Ferulic Acid 300SB C18.M (Sequence Method)
Last changed : 10/4/2024 7:53:25 AM by SYSTEM
(modified after loading)

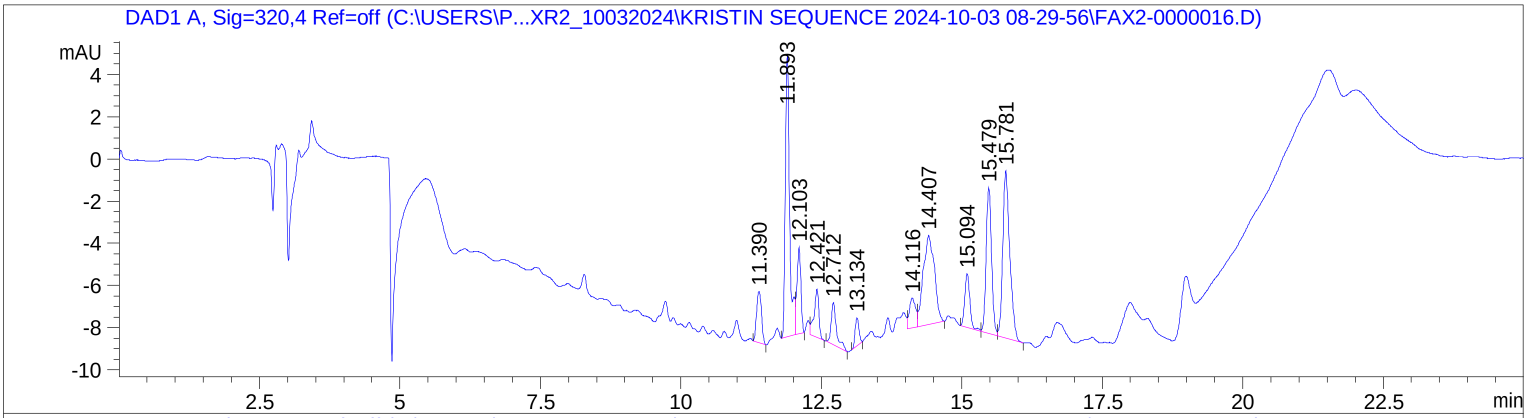

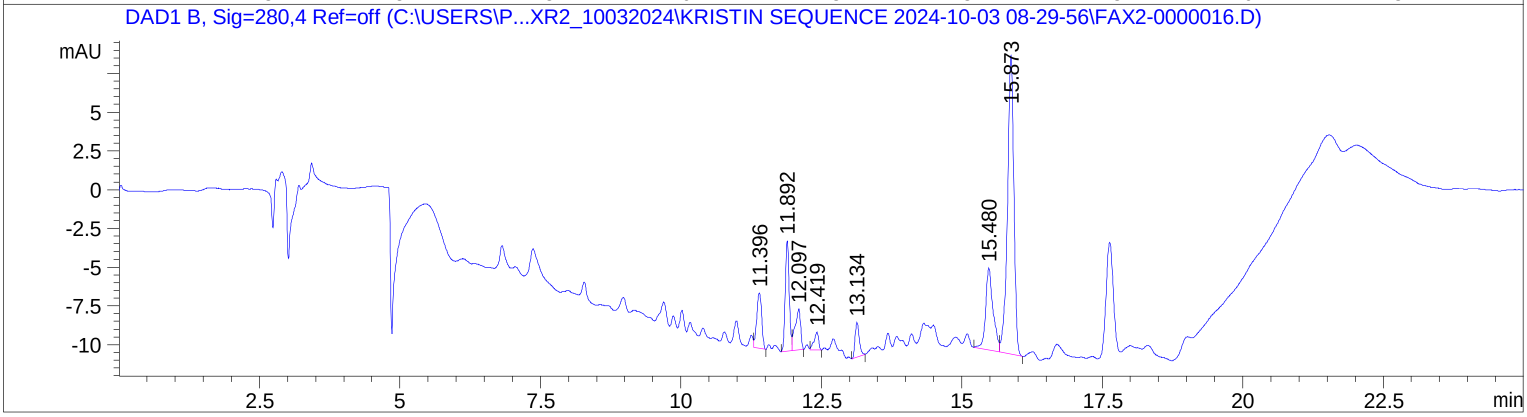

===

Fraction Information

===

No Fractions found.

===

===

===

External Standard Report

===

Sorted By : Signal
Calib. Data Modified : Friday, October 4, 20247:53:25 AM
Multiplier : 1.0000
Dilution : 1.0000
Do not use Multiplier & Dilution Factor with ISTDs

Signal 1: DAD1 A, Sig=320,4 Ref=off

RetTime [min]	Type	Area [mAU*s]	Amt/Area	Amount [ng/ul]	Grp	Name
11.893	BV R	70.77197	3.77905e-2	2.67451		Ferulic Acid

Totals : 2.67451

Signal 2: DAD1 B, Sig=280,4 Ref=off

RetTime [min]	Type	Area [mAU*s]	Amt/Area	Amount [ng/ul]	Grp	Name
15.873	VB	152.25772	2.23668e-2	3.40551		Cinnamic Acid

Totals : 3.40551

===

*** End of Report ***

Data File C:\USERS\P...\FAXR2_10032024\KRISTIN SEQUENCE 2024-10-03 08-29-56\FAX2-0000017.D
Sample Name: FAX 36

===

Acq. Operator : SYSTEM Seq. Line : 17
Sample Operator : SYSTEM
Acq. Instrument : LC Location : P1-B-06
Injection Date : 10/3/2024 3:26:28 PM Inj : 1
Inj Volume : 5.000 µl
Different Inj Volume from Sample Entry! Actual Inj Volume : 10.000 µl
Acq. Method : C:\Users\Public\Documents\ChemStation\1\Data\FAXr2_10032024\Kristin Sequence 2024-10-03 08-29-56\Ferulic Acid 300SB C18.M
Last changed : 10/2/2024 9:37:39 AM by SYSTEM
Analysis Method : C:\Users\Public\Documents\ChemStation\1\Data\FAXr2_10032024\Kristin Sequence 2024-10-03 08-29-56\Ferulic Acid 300SB C18.M (Sequence Method)
Last changed : 10/4/2024 7:53:25 AM by SYSTEM
(modified after loading)

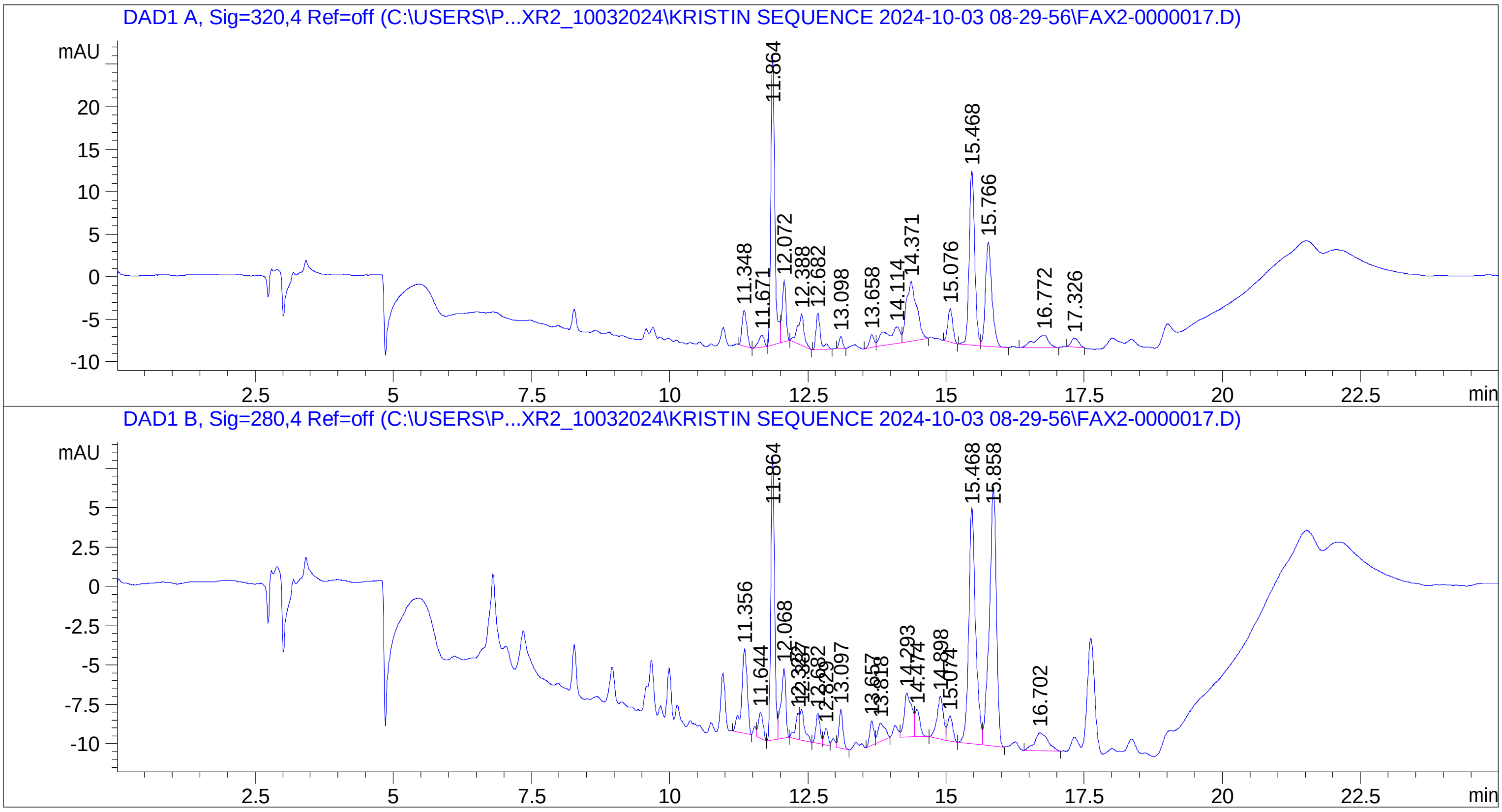

===

Fraction Information

===

No Fractions found.

===

===

===

External Standard Report

===

Sorted By : Signal
Calib. Data Modified : Friday, October 4, 20247:53:25 AM
Multiplier : 1.0000
Dilution : 1.0000
Do not use Multiplier & Dilution Factor with ISTDs

Signal 1: DAD1 A, Sig=320,4 Ref=off

RetTime [min]	Type	Area [mAU*s]	Amt/Area	Amount [ng/ul]	Grp	Name
11.864	BV	170.33751	3.21683e-2	5.47947		Ferulic Acid

Totals : 5.47947

Signal 2: DAD1 B, Sig=280,4 Ref=off

RetTime [min]	Type	Area [mAU*s]	Amt/Area	Amount [ng/ul]	Grp	Name
15.858	VB	144.09122	2.26093e-2	3.25780		Cinnamic Acid

Totals : 3.25780

*** End of Report ***

Data File C:\USERS\P...\FAXR2_10032024\KRISTIN SEQUENCE 2024-10-03 08-29-56\FAX2-0000018.D
Sample Name: FAX 37

===

```
Acq. Operator   : SYSTEM                          Seq. Line :  18
Sample Operator : SYSTEM
Acq. Instrument : LC                               Location :   P1-B-07
Injection Date  : 10/3/2024 3:52:27 PM                  Inj :   1
                                                 Inj Volume : 5.000 µl
Different Inj Volume from Sample Entry!  Actual Inj Volume : 10.000 µl
Acq. Method     : C:\Users\Public\Documents\ChemStation\1\Data\FAXr2_10032024\Kristin
                  Sequence 2024-10-03 08-29-56\Ferulic Acid 300SB C18.M
Last changed    : 10/2/2024 9:37:39 AM by SYSTEM
Analysis Method : C:\Users\Public\Documents\ChemStation\1\Data\FAXr2_10032024\Kristin
                  Sequence 2024-10-03 08-29-56\Ferulic Acid 300SB C18.M (Sequence Method)
Last changed    : 10/4/2024 7:53:25 AM by SYSTEM
                  (modified after loading)
```

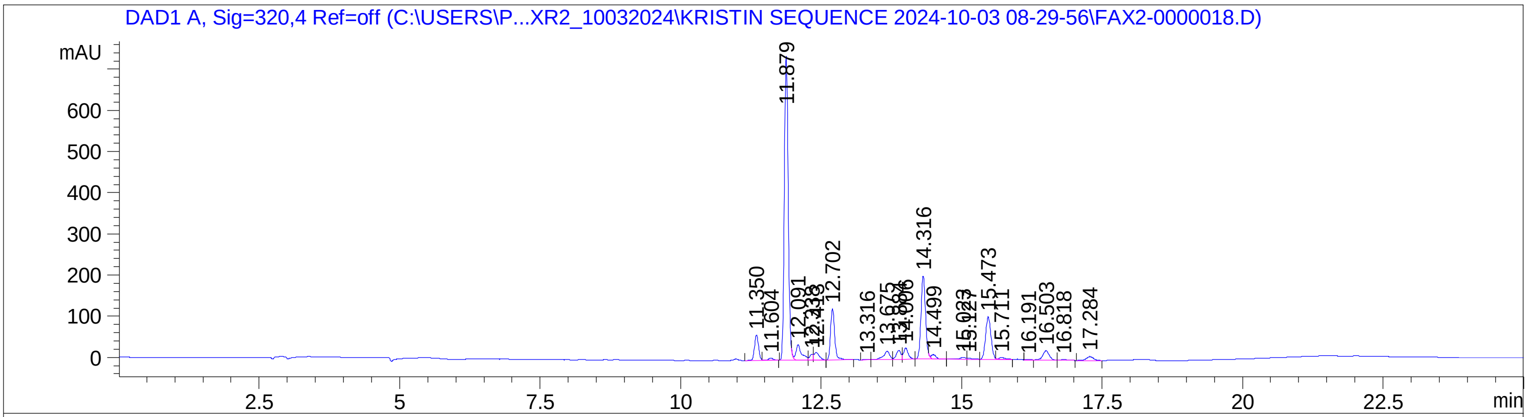

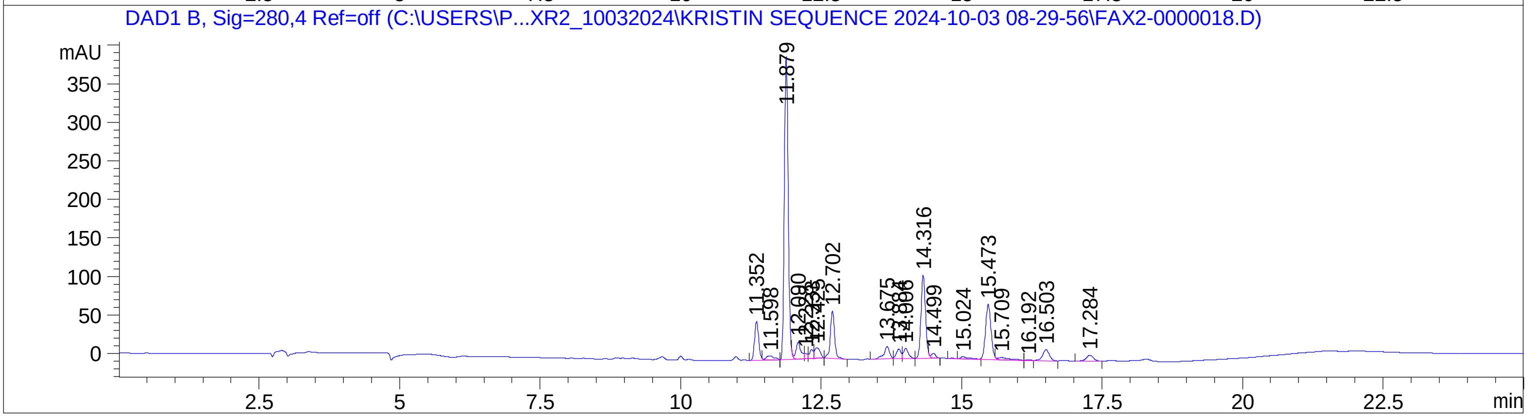

===

Fraction Information

===

No Fractions found.

===

===

===

External Standard Report

===

```
Sorted By             :      Signal
Calib. Data Modified  :      Friday, October 4, 20247:53:25 AM
Multiplier            :      1.0000
Dilution              :      1.0000
Do not use Multiplier & Dilution Factor with ISTDs
```

Signal 1: DAD1 A, Sig=320,4 Ref=off

RetTime [min]	Type	Area [mAU*s]	Amt/Area	Amount [ng/ul]	Grp	Name
11.879	BV R	3566.90747	2.83629e-2	101.16768		Ferulic Acid

Totals : 101.16768

Signal 2: DAD1 B, Sig=280,4 Ref=off

RetTime [min]	Type	Area [mAU*s]	Amt/Area	Amount [ng/ul]	Grp	Name
16.192	BV E	5.45264	1.37599e-1	7.50277e-1		Cinnamic Acid

Totals : 7.50277e-1

==

*** End of Report ***

Data File C:\USERS\P...\FAXR2_10032024\KRISTIN SEQUENCE 2024-10-03 08-29-56\FAX2-0000019.D
Sample Name: FAX 38

===

Acq. Operator : SYSTEM Seq. Line : 19
Sample Operator : SYSTEM
Acq. Instrument : LC Location : P1-B-08
Injection Date : 10/3/2024 4:18:24 PM Inj : 1
Inj Volume : 5.000 µl
Different Inj Volume from Sample Entry! Actual Inj Volume : 10.000 µl
Acq. Method : C:\Users\Public\Documents\ChemStation\1\Data\FAXr2_10032024\Kristin Sequence 2024-10-03 08-29-56\Ferulic Acid 300SB C18.M
Last changed : 10/2/2024 9:37:39 AM by SYSTEM
Analysis Method : C:\Users\Public\Documents\ChemStation\1\Data\FAXr2_10032024\Kristin Sequence 2024-10-03 08-29-56\Ferulic Acid 300SB C18.M (Sequence Method)
Last changed : 10/4/2024 7:53:25 AM by SYSTEM
(modified after loading)

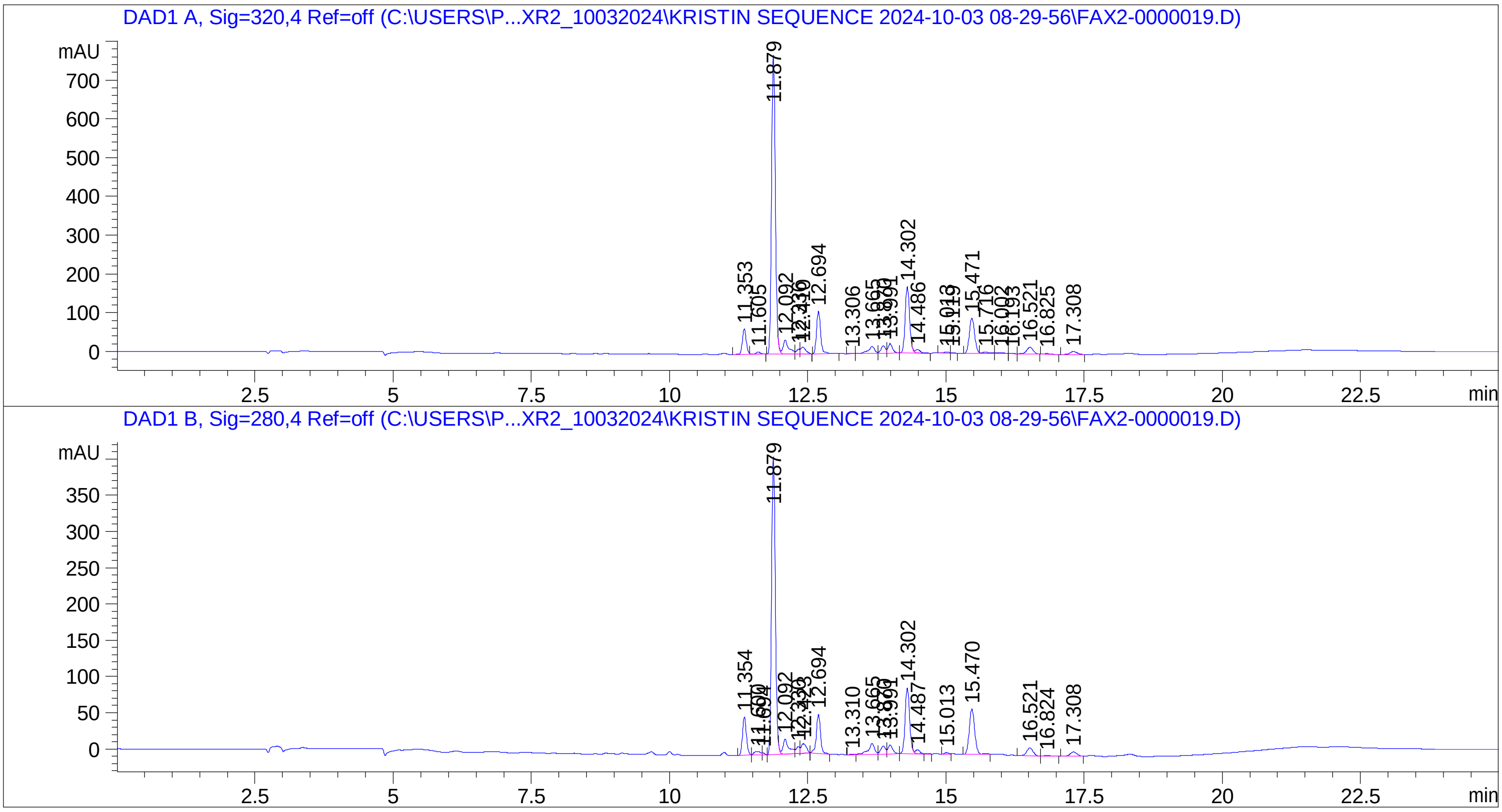

===

Fraction Information

===

No Fractions found.

===

===

===

External Standard Report

===

Sorted By : Signal
Calib. Data Modified : Friday, October 4, 20247:53:25 AM
Multiplier : 1.0000
Dilution : 1.0000
Do not use Multiplier & Dilution Factor with ISTDs

Signal 1: DAD1 A, Sig=320,4 Ref=off

RetTime [min]	Type	Area [mAU*s]	Amt/Area	Amount [ng/ul]	Grp	Name
11.879	BV R	3713.68481	2.83553e-2	105.30269		Ferulic Acid

Totals : 105.30269

Signal 2: DAD1 B, Sig=280,4 Ref=off

RetTime [min]	Type	Area [mAU*s]	Amt/Area	Amount [ng/ul]	Grp	Name
16.521	BB	92.38574	2.51404e-2	2.32262		Cinnamic Acid

Totals : 2.32262

===

*** End of Report ***

===

Acq. Operator : SYSTEM Seq. Line : 20
Sample Operator : SYSTEM
Acq. Instrument : LC Location : P1-B-09
Injection Date : 10/3/2024 4:44:22 PM Inj : 1
Inj Volume : 5.000 µl
Different Inj Volume from Sample Entry! Actual Inj Volume : 10.000 µl
Acq. Method : C:\Users\Public\Documents\ChemStation\1\Data\FAXr2_10032024\Kristin Sequence 2024-10-03 08-29-56\Ferulic Acid 300SB C18.M
Last changed : 10/2/2024 9:37:39 AM by SYSTEM
Analysis Method : C:\Users\Public\Documents\ChemStation\1\Data\FAXr2_10032024\Kristin Sequence 2024-10-03 08-29-56\Ferulic Acid 300SB C18.M (Sequence Method)
Last changed : 10/4/2024 7:53:25 AM by SYSTEM
(modified after loading)

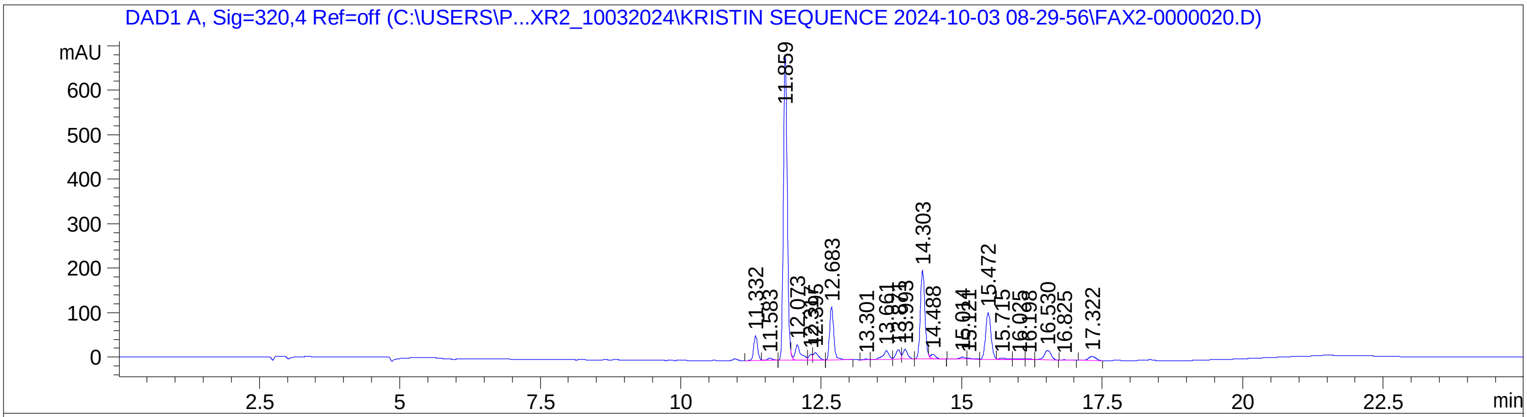

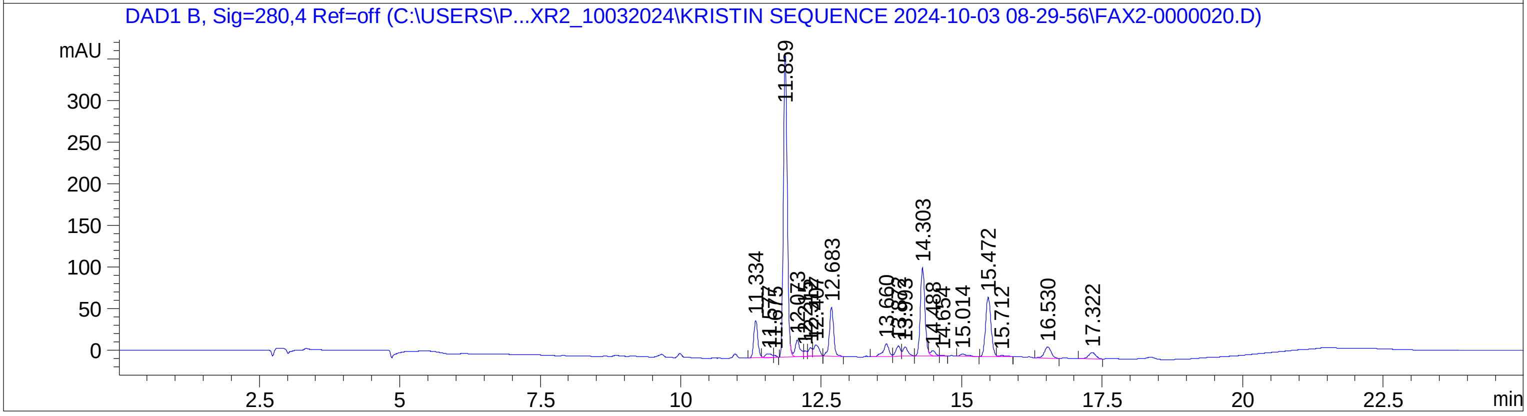

===

Fraction Information

===

No Fractions found.

===

===

===

External Standard Report

===

Sorted By : Signal
Calib. Data Modified : Friday, October 4, 20247:53:25 AM
Multiplier : 1.0000
Dilution : 1.0000
Do not use Multiplier & Dilution Factor with ISTDs

Signal 1: DAD1 A, Sig=320,4 Ref=off

RetTime [min]	Type	Area [mAU*s]	Amt/Area	Amount [ng/ul]	Grp	Name
11.859	BV R	3302.54639	2.83781e-2	93.72010		Ferulic Acid

Totals : 93.72010

Signal 2: DAD1 B, Sig=280,4 Ref=off

RetTime [min]	Type	Area [mAU*s]	Amt/Area	Amount [ng/ul]	Grp	Name
16.530	BB	115.63055	2.37225e-2	2.74304		Cinnamic Acid

Totals : 2.74304

===

*** End of Report ***

Data File C:\Users\P...henolics07122024\Kristin Sequence 2024-10-02 09-40-51\PT3-0000009.D
Sample Name: FAX 4

===
Acq. Operator   : SYSTEM   Seq. Line :   9
Sample Operator : SYSTEM
Acq. Instrument : LC   Location :   P1-A-10
Injection Date : 10/2/2024 1:09:36 PM   Inj :   1
                                                 Inj Volume : 5.000 µl
Different Inj Volume from Sample Entry! Actual Inj Volume : 10.000 µl
Acq. Method   : C:\Users\Public\Documents\ChemStation\1\Data\R1Phenolics07122024\Kristin Sequence 2024-10-02 09-40-51\Ferulic Acid 300SB C18.M
Last changed   : 10/2/2024 9:37:39 AM by SYSTEM
Analysis Method : C:\Users\Public\Documents\ChemStation\1\Data\R1Phenolics07122024\Kristin Sequence 2024-10-02 09-40-51\Ferulic Acid 300SB C18.M (Sequence Method)
Last changed   : 10/3/2024 8:35:21 AM by SYSTEM
                  (modified after loading)

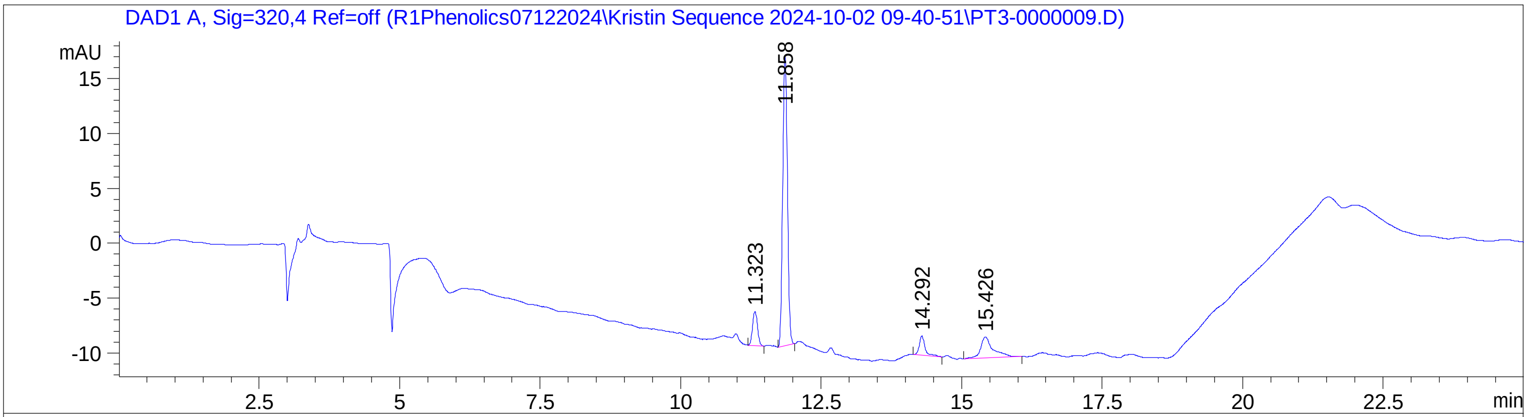

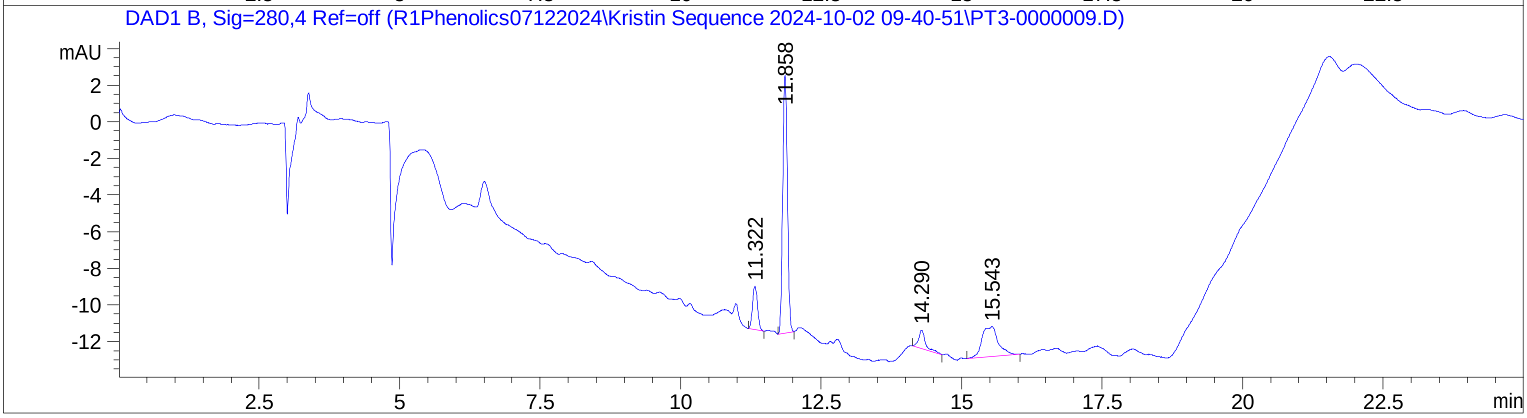

===

Fraction Information

===

No Fractions found.

===

===

===

External Standard Report

===

Sorted By   :   Signal
Calib. Data Modified :   Thursday, October 3, 20248:35:21 AM
Multiplier   :   1.0000
Dilution   :   1.0000
Do not use Multiplier & Dilution Factor with ISTDs

Signal 1: DAD1 A, Sig=320,4 Ref=off

RetTime [min]	Type	Area [mAU*s]	Amt/Area	Amount [ng/ul]	Grp	Name
11.858	BB	154.85275	3.06493e-2	4.74612		Ferulic Acid

Totals : 4.74612

Signal 2: DAD1 B, Sig=280,4 Ref=off

RetTime [min]	Type	Area [mAU*s]	Amt/Area	Amount [ng/ul]	Grp	Name
16.111		-	-	-		Cinnamic Acid

Totals : 0.00000

1 Warnings or Errors :

Warning : Calibrated compound(s) not found

===

*** End of Report ***

Data File C:\USERS\P...\FAXR2_10032024\KRISTIN SEQUENCE 2024-10-03 08-29-56\FAX2-0000021.D
Sample Name: FAX 40

===
Acq. Operator   : SYSTEM   Seq. Line : 21
Sample Operator : SYSTEM
Acq. Instrument : LC   Location :   P1-B-10
Injection Date : 10/3/2024 5:10:20 PM   Inj :   1
                                              Inj Volume : 5.000 µl
Different Inj Volume from Sample Entry! Actual Inj Volume : 10.000 µl
Acq. Method   : C:\Users\Public\Documents\ChemStation\1\Data\FAXr2_10032024\Kristin Sequence 2024-10-03 08-29-56\Ferulic Acid 300SB C18.M
Last changed   : 10/2/2024 9:37:39 AM by SYSTEM
Analysis Method : C:\Users\Public\Documents\ChemStation\1\Data\FAXr2_10032024\Kristin Sequence 2024-10-03 08-29-56\Ferulic Acid 300SB C18.M (Sequence Method)
Last changed   : 10/4/2024 7:53:25 AM by SYSTEM
(modified after loading)

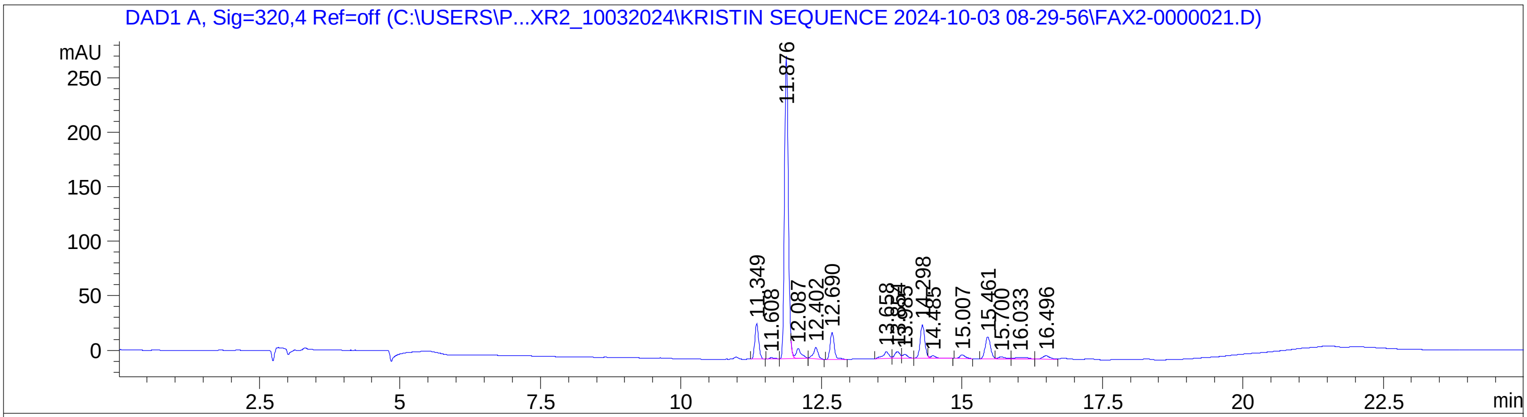

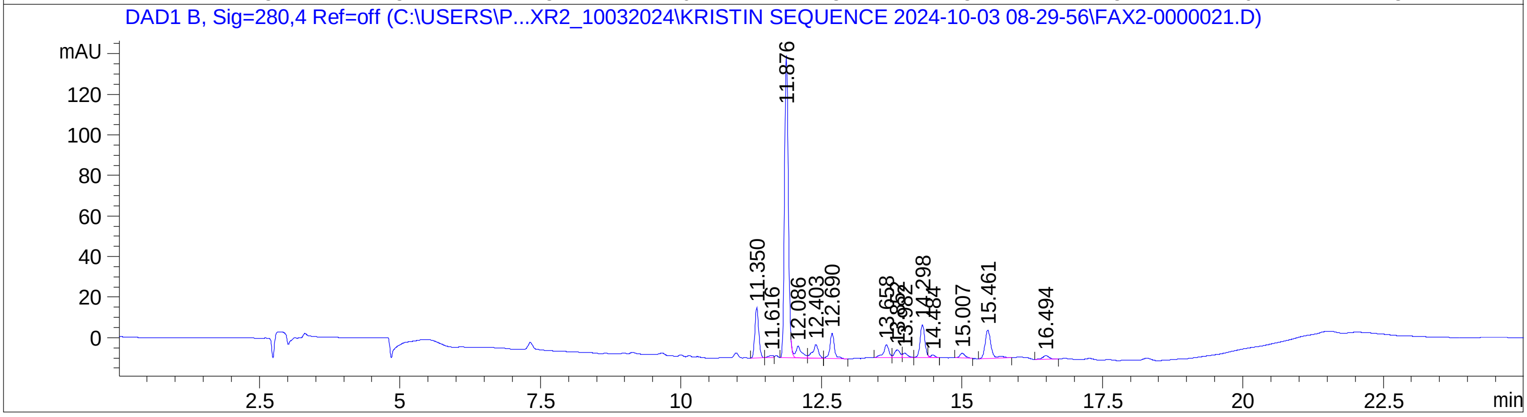

===

Fraction Information

===

No Fractions found.

===

===

===

External Standard Report

===

Sorted By   :   Signal
Calib. Data Modified :   Friday, October 4, 20247:53:25 AM
Multiplier   :   1.0000
Dilution   :   1.0000
Do not use Multiplier & Dilution Factor with ISTDs

Signal 1: DAD1 A, Sig=320,4 Ref=off

RetTime [min]	Type	Area [mAU*s]	Amt/Area	Amount [ng/ul]	Grp	Name
11.876	BV R	1327.62317	2.86847e-2	38.08254		Ferulic Acid

Totals : 38.08254

Signal 2: DAD1 B, Sig=280,4 Ref=off

RetTime [min]	Type	Area [mAU*s]	Amt/Area	Amount [ng/ul]	Grp	Name
16.494	BB	14.27441	6.37389e-2	9.09835e-1		Cinnamic Acid

Totals : 9.09835e-1

*** End of Report ***

Data File C:\USERS\P...\FAXR2_10032024\KRISTIN SEQUENCE 2024-10-03 08-29-56\FAX2-0000022.D
Sample Name: FAX 41

===

Acq. Operator : SYSTEM   Seq. Line : 22
Sample Operator : SYSTEM
Acq. Instrument : LC   Location : P1-B-11
Injection Date : 10/3/2024 5:36:19 PM   Inj : 1
Inj Volume : 5.000 µl
Different Inj Volume from Sample Entry! Actual Inj Volume : 10.000 µl
Acq. Method : C:\Users\Public\Documents\ChemStation\1\Data\FAXr2_10032024\Kristin Sequence 2024-10-03 08-29-56\Ferulic Acid 300SB C18.M
Last changed : 10/2/2024 9:37:39 AM by SYSTEM
Analysis Method : C:\Users\Public\Documents\ChemStation\1\Data\FAXr2_10032024\Kristin Sequence 2024-10-03 08-29-56\Ferulic Acid 300SB C18.M (Sequence Method)
Last changed : 10/4/2024 7:53:25 AM by SYSTEM
(modified after loading)

DAD1 A, Sig=320,4 Ref=off (C:\USERS\P...XR2_10032024\KRISTIN SEQUENCE 2024-10-03 08-29-56\FAX2-0000022.D)

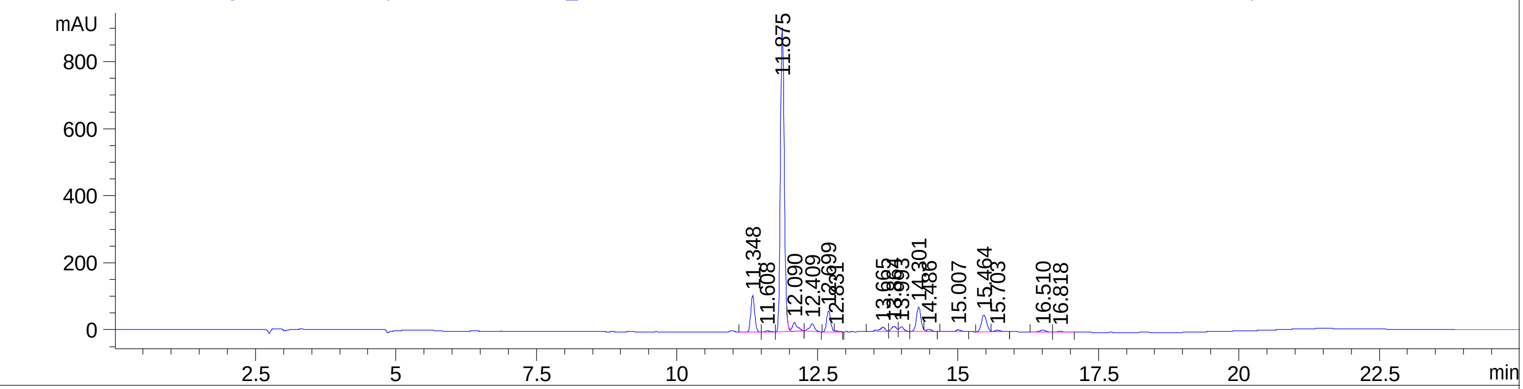

DAD1 B, Sig=280,4 Ref=off (C:\USERS\P...XR2_10032024\KRISTIN SEQUENCE 2024-10-03 08-29-56\FAX2-0000022.D)

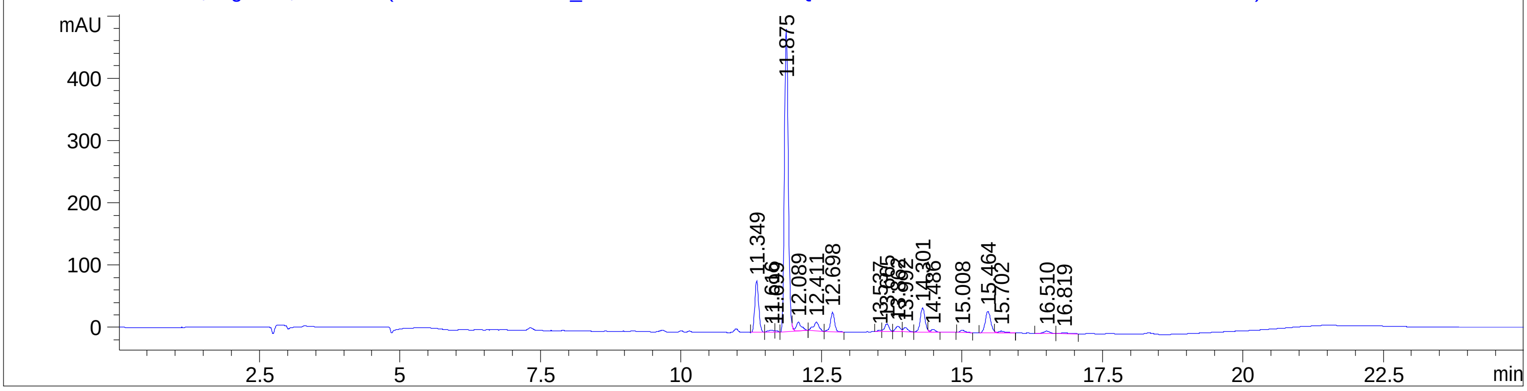

===

Fraction Information

===

No Fractions found.

===
===
===

External Standard Report

===

Sorted By : Signal
Calib. Data Modified : Friday, October 4, 20247:53:25 AM
Multiplier : 1.0000
Dilution : 1.0000
Do not use Multiplier & Dilution Factor with ISTDs

Signal 1: DAD1 A, Sig=320,4 Ref=off

RetTime [min]	Type	Area [mAU*s]	Amt/Area	Amount [ng/ul]	Grp	Name
11.875	BV R	4344.47266	2.83287e-2	123.07326		Ferulic Acid

Totals : 123.07326

Signal 2: DAD1 B, Sig=280,4 Ref=off

RetTime [min]	Type	Area [mAU*s]	Amt/Area	Amount [ng/ul]	Grp	Name
16.510	BV	33.19912	3.77155e-2	1.25212		Cinnamic Acid

Totals : 1.25212

===

*** End of Report ***

Data File C:\USERS\P...\FAXR2_10032024\KRISTIN SEQUENCE 2024-10-03 08-29-56\FAX2-0000023.D
Sample Name: FAX 42

===
Acq. Operator   : SYSTEM   Seq. Line :   23
Sample Operator : SYSTEM
Acq. Instrument : LC   Location :   P1-C-01
Injection Date : 10/3/2024 6:02:20 PM   Inj :   1
                                                     Inj Volume : 5.000 µl
Different Inj Volume from Sample Entry! Actual Inj Volume : 10.000 µl
Acq. Method   : C:\Users\Public\Documents\ChemStation\1\Data\FAXr2_10032024\Kristin
                  Sequence 2024-10-03 08-29-56\Ferulic Acid 300SB C18.M
Last changed   : 10/2/2024 9:37:39 AM by SYSTEM
Analysis Method : C:\Users\Public\Documents\ChemStation\1\Data\FAXr2_10032024\Kristin
                  Sequence 2024-10-03 08-29-56\Ferulic Acid 300SB C18.M (Sequence Method)
Last changed   : 10/4/2024 7:53:25 AM by SYSTEM
                  (modified after loading)

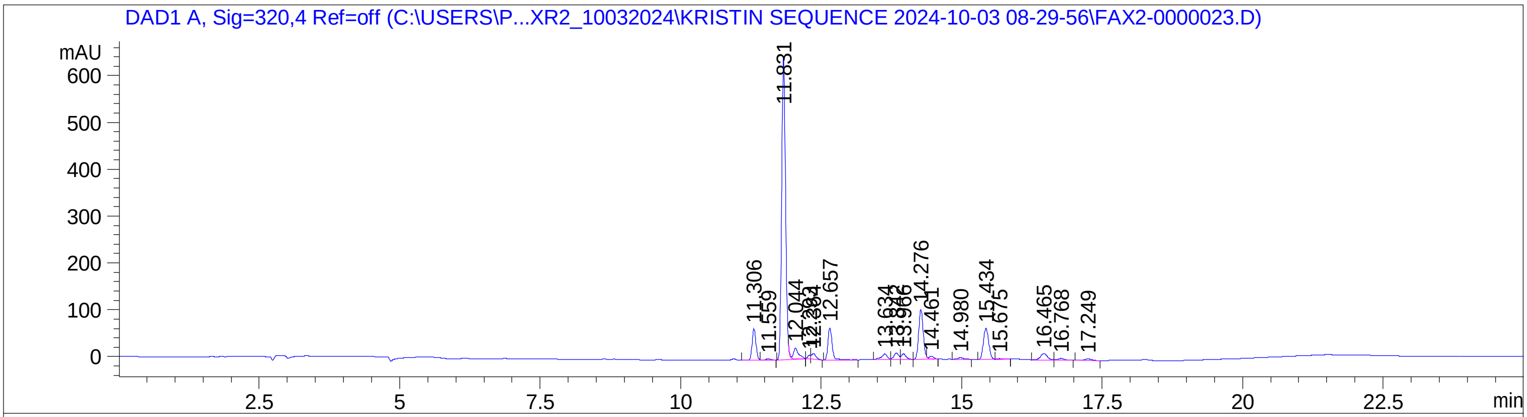

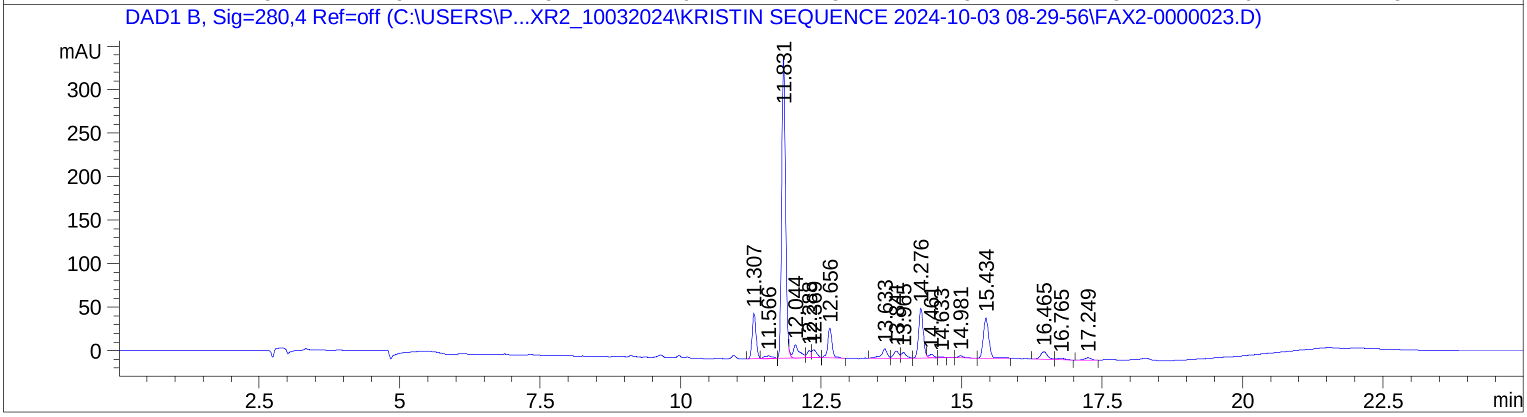

===
                           Fraction Information
===
No Fractions found.
===
===
===
                      External Standard Report
===

Sorted By   :   Signal
Calib. Data Modified :   Friday, October 4, 20247:53:25 AM
Multiplier   :   1.0000
Dilution   :   1.0000
Do not use Multiplier & Dilution Factor with ISTDs

Signal 1: DAD1 A, Sig=320,4 Ref=off

RetTime [min]	Type	Area [mAU*s]	Amt/Area	Amount [ng/ul]	Grp	Name
11.831	BV R	3122.43359	2.83900e-2	88.64596		Ferulic Acid

Totals : 88.64596

Signal 2: DAD1 B, Sig=280,4 Ref=off

RetTime [min]	Type	Area [mAU*s]	Amt/Area	Amount [ng/ul]	Grp	Name
16.465	BV	77.51026	2.64942e-2	2.05357		Cinnamic Acid

Totals : 2.05357

*** End of Report ***

===

Acq. Operator	: SYSTEM	Seq. Line :	24
Sample Operator	: SYSTEM		
Acq. Instrument	: LC	Location :	P1-C-02
Injection Date	: 10/3/2024 6:28:18 PM	Inj :	1
		Inj Volume :	5.000 µl

Different Inj Volume from Sample Entry! Actual Inj Volume : 10.000 µl

Acq. Method : C:\Users\Public\Documents\ChemStation\1\Data\FAXr2_10032024\Kristin Sequence 2024-10-03 08-29-56\Ferulic Acid 300SB C18.M
Last changed : 10/2/2024 9:37:39 AM by SYSTEM
Analysis Method : C:\Users\Public\Documents\ChemStation\1\Data\FAXr2_10032024\Kristin Sequence 2024-10-03 08-29-56\Ferulic Acid 300SB C18.M (Sequence Method)
Last changed : 10/4/2024 7:53:25 AM by SYSTEM
(modified after loading)

DAD1 A, Sig=320,4 Ref=off (C:\USERS\P...XR2_10032024\KRISTIN SEQUENCE 2024-10-03 08-29-56\FAX2-0000024.D)

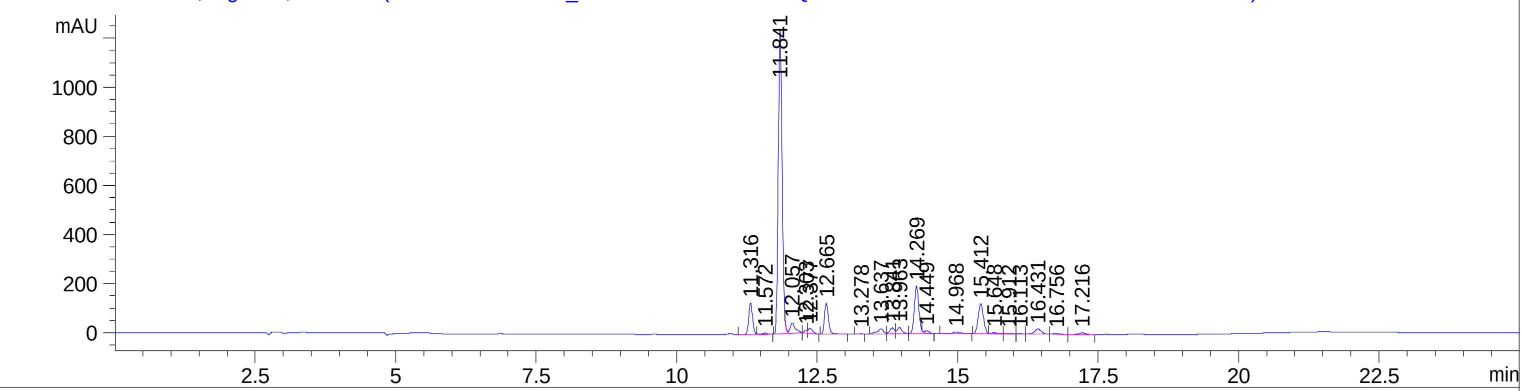

DAD1 B, Sig=280,4 Ref=off (C:\USERS\P...XR2_10032024\KRISTIN SEQUENCE 2024-10-03 08-29-56\FAX2-0000024.D)

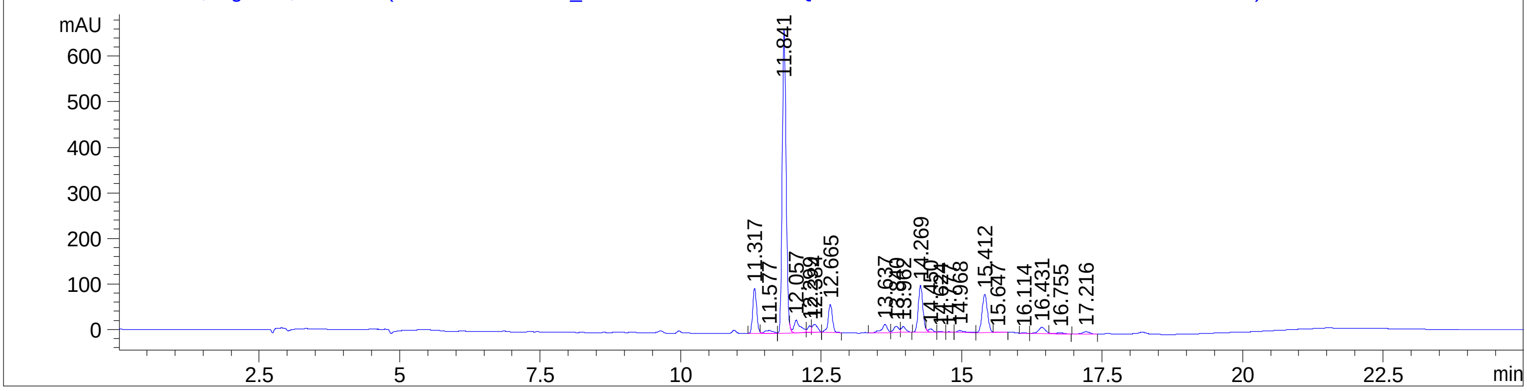

===

Fraction Information

===

No Fractions found.

===

===

===

External Standard Report

===

Sorted By	:	Signal
Calib. Data Modified	:	Friday, October 4, 20247:53:25 AM
Multiplier	:	1.0000
Dilution	:	1.0000

Do not use Multiplier & Dilution Factor with ISTDs

Signal 1: DAD1 A, Sig=320,4 Ref=off

RetTime [min]	Type	Area [mAU*s]	Amt/Area	Amount [ng/ul]	Grp	Name
11.841	BV R	6001.61035	2.82854e-2	169.75816		Ferulic Acid

Totals : 169.75816

Signal 2: DAD1 B, Sig=280,4 Ref=off

RetTime [min]	Type	Area [mAU*s]	Amt/Area	Amount [ng/ul]	Grp	Name
16.114	BB	5.48029	1.36996e-1	7.50777e-1		Cinnamic Acid

Totals : 7.50777e-1

===

*** End of Report ***

===

```
Acq. Operator   : SYSTEM                          Seq. Line :  25
Sample Operator : SYSTEM
Acq. Instrument : LC                               Location :   P1-C-03
Injection Date  : 10/3/2024 6:54:17 PM                  Inj :   1
                                                 Inj Volume : 5.000 µl
Different Inj Volume from Sample Entry!  Actual Inj Volume : 10.000 µl
Acq. Method     : C:\Users\Public\Documents\ChemStation\1\Data\FAXr2_10032024\Kristin
                  Sequence 2024-10-03 08-29-56\Ferulic Acid 300SB C18.M
Last changed    : 10/2/2024 9:37:39 AM by SYSTEM
Analysis Method : C:\Users\Public\Documents\ChemStation\1\Data\FAXr2_10032024\Kristin
                  Sequence 2024-10-03 08-29-56\Ferulic Acid 300SB C18.M (Sequence Method)
Last changed    : 10/4/2024 7:53:25 AM by SYSTEM
                  (modified after loading)
```

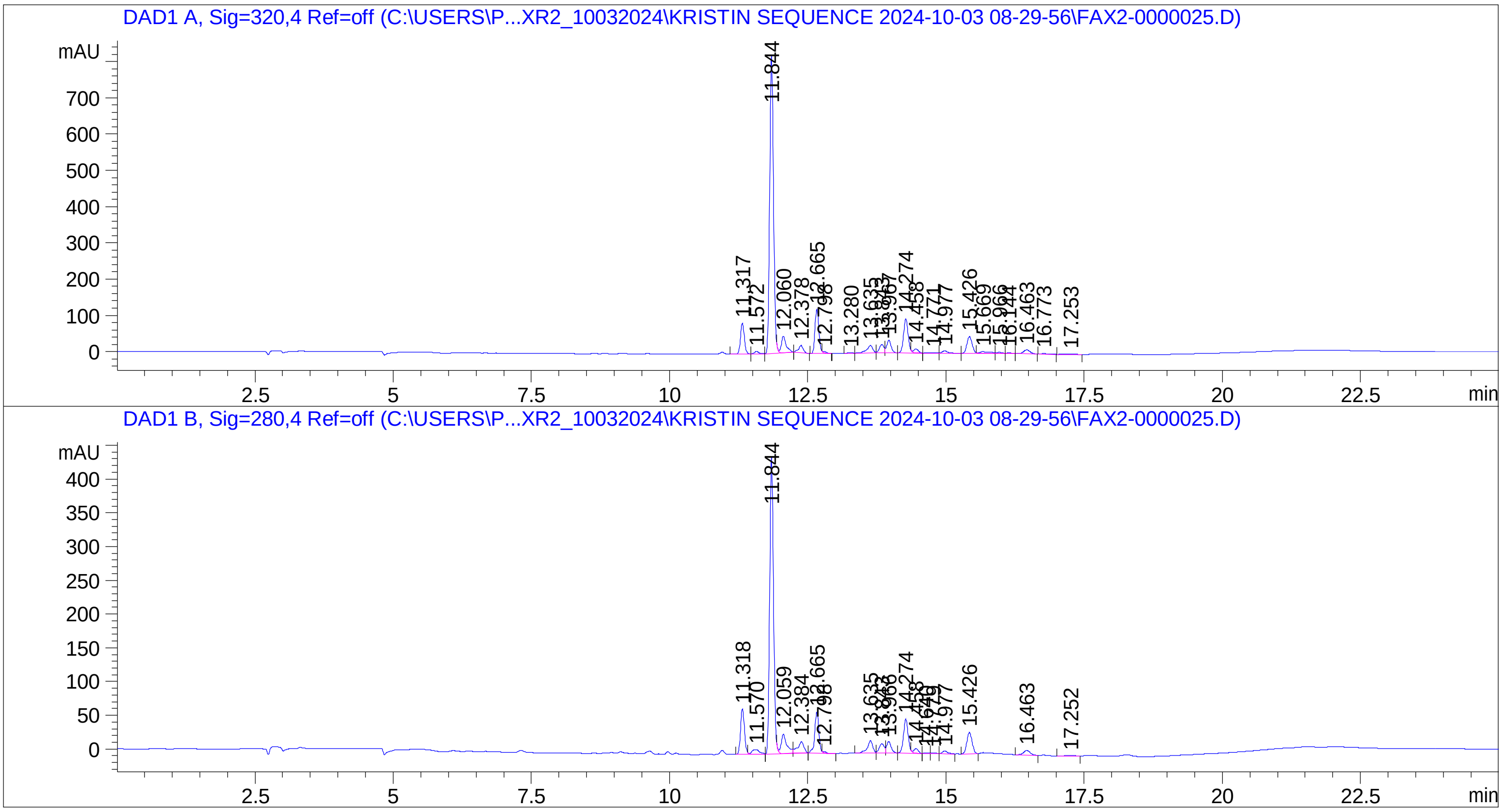

===

Fraction Information

===

No Fractions found.

===

===

===

External Standard Report

===

```
Sorted By              :      Signal
Calib. Data Modified   :      Friday, October 4, 20247:53:25 AM
Multiplier             :      1.0000
Dilution               :      1.0000
Do not use Multiplier & Dilution Factor with ISTDs
```

Signal 1: DAD1 A, Sig=320,4 Ref=off

RetTime [min]	Type	Area [mAU*s]	Amt/Area	Amount [ng/ul]	Grp	Name
11.844	BV R	3944.54663	2.83446e-2	111.80654		Ferulic Acid

Totals : 111.80654

Signal 2: DAD1 B, Sig=280,4 Ref=off

RetTime [min]	Type	Area [mAU*s]	Amt/Area	Amount [ng/ul]	Grp	Name
16.463	BB	56.72940	2.95739e-2	1.67771		Cinnamic Acid

Totals : 1.67771

*** End of Report ***

```
=====================================================================
Acq. Operator   : SYSTEM                        Seq. Line :  26
Sample Operator : SYSTEM
Acq. Instrument : LC                             Location :   P1-C-04
Injection Date  : 10/3/2024 7:20:16 PM                Inj :   1
                                               Inj Volume : 5.000 µl
Different Inj Volume from Sample Entry!  Actual Inj Volume : 10.000 µl
Acq. Method     : C:\Users\Public\Documents\ChemStation\1\Data\FAXr2_10032024\Kristin
                  Sequence 2024-10-03 08-29-56\Ferulic Acid 300SB C18.M
Last changed    : 10/2/2024 9:37:39 AM by SYSTEM
Analysis Method : C:\Users\Public\Documents\ChemStation\1\Data\FAXr2_10032024\Kristin
                  Sequence 2024-10-03 08-29-56\Ferulic Acid 300SB C18.M (Sequence Method)
Last changed    : 10/4/2024 7:53:25 AM by SYSTEM
                  (modified after loading)
```

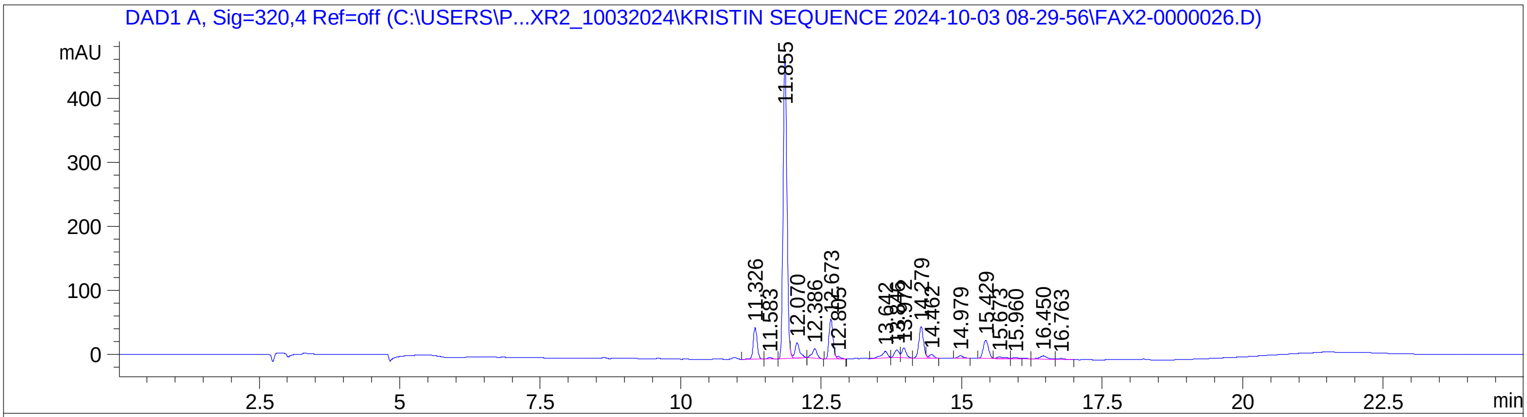

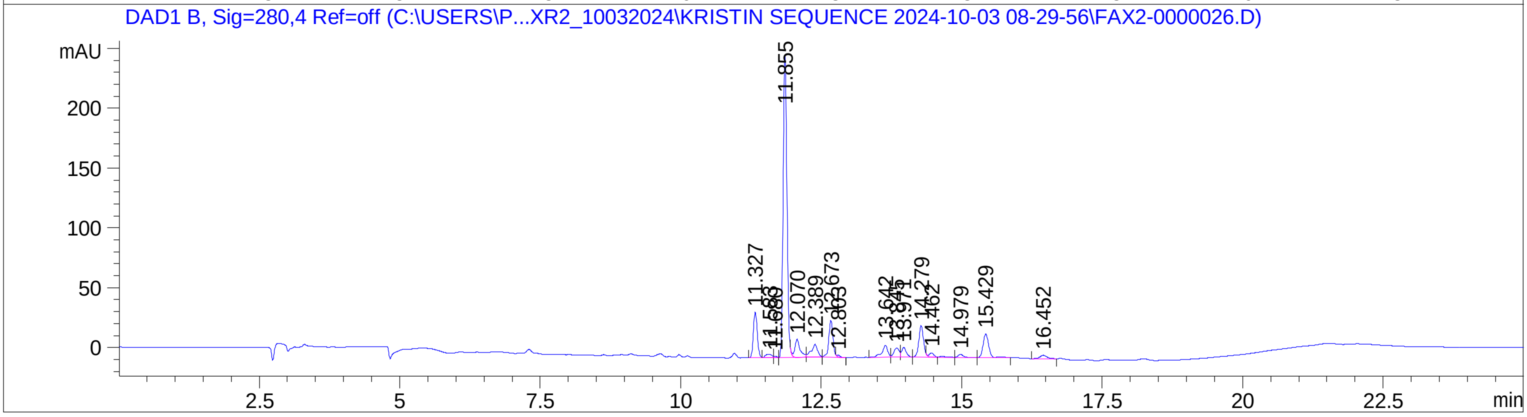


```
==================================================================================
                           Fraction Information
==================================================================================
No Fractions found.
==================================================================================
==================================================================================
=====================================================================
                      External Standard Report
=====================================================================

Sorted By             :      Signal
Calib. Data Modified  :      Friday, October 4, 20247:53:25 AM
Multiplier            :      1.0000
Dilution              :      1.0000
Do not use Multiplier & Dilution Factor with ISTDs
```

Signal 1: DAD1 A, Sig=320,4 Ref=off

RetTime [min]	Type	Area [mAU*s]	Amt/Area	Amount [ng/ul]	Grp	Name
11.855	BV R	2273.10034	2.84715e-2	64.71853		Ferulic Acid

Totals : 64.71853

Signal 2: DAD1 B, Sig=280,4 Ref=off

RetTime [min]	Type	Area [mAU*s]	Amt/Area	Amount [ng/ul]	Grp	Name
16.452	BB	25.65460	4.34880e-2	1.11567		Cinnamic Acid

Totals : 1.11567

==

*** End of Report ***

```
=====================================================================
Acq. Operator   : SYSTEM                          Seq. Line :  10
Sample Operator : SYSTEM
Acq. Instrument : LC                               Location :   P1-A-11
Injection Date  : 10/2/2024 1:35:34 PM                  Inj :   1
                                                Inj Volume : 5.000 µl
Different Inj Volume from Sample Entry!  Actual Inj Volume : 10.000 µl
Acq. Method     : C:\Users\Public\Documents\ChemStation\1\Data\R1Phenolics07122024\Kristin
                  Sequence 2024-10-02 09-40-51\Ferulic Acid 300SB C18.M
Last changed    : 10/2/2024 9:37:39 AM by SYSTEM
Analysis Method : C:\Users\Public\Documents\ChemStation\1\Data\R1Phenolics07122024\Kristin
                  Sequence 2024-10-02 09-40-51\Ferulic Acid 300SB C18.M (Sequence Method)
Last changed    : 10/3/2024 8:35:21 AM by SYSTEM
                  (modified after loading)
```

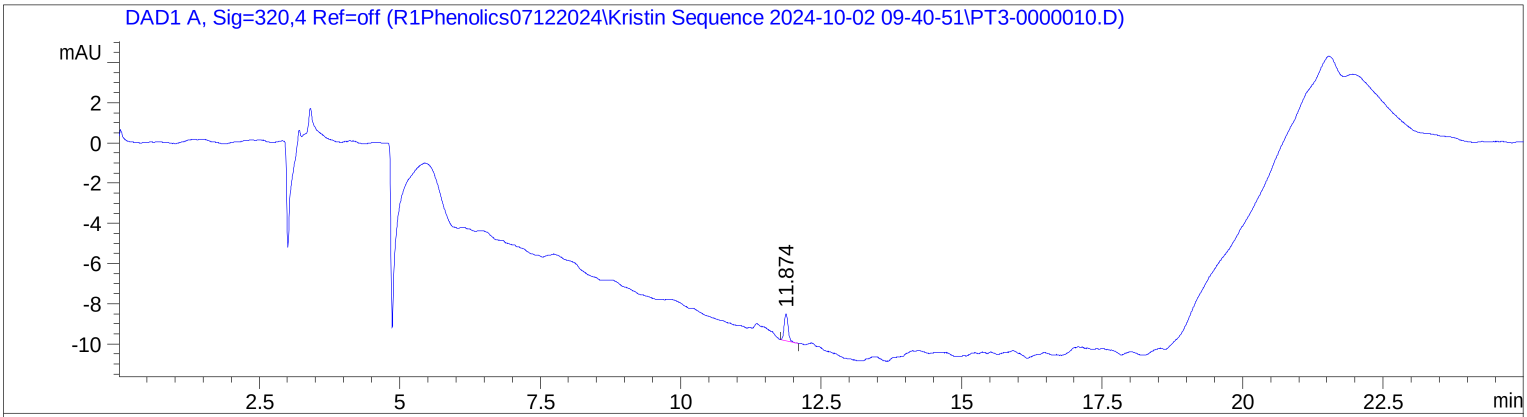

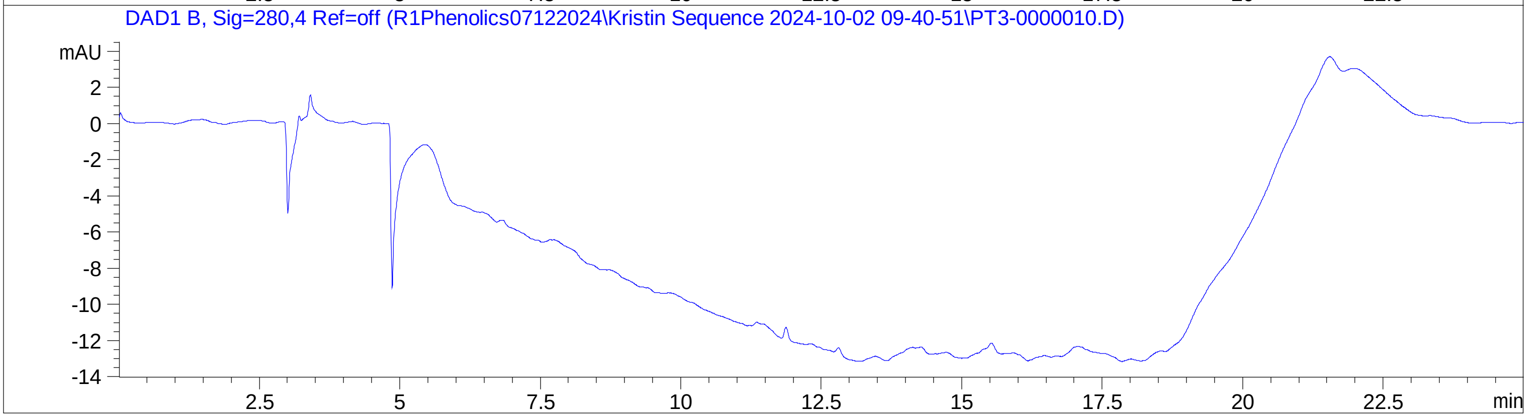


```
=================================================================================
                           Fraction Information
=================================================================================
No Fractions found.
=================================================================================
=================================================================================
=====================================================================
                      External Standard Report
=====================================================================

Sorted By             :      Signal
Calib. Data Modified  :      Thursday, October 3, 20248:35:21 AM
Multiplier            :      1.0000
Dilution              :      1.0000
Do not use Multiplier & Dilution Factor with ISTDs
```

Signal 1: DAD1 A, Sig=320,4 Ref=off

RetTime [min]	Type	Area [mAU*s]	Amt/Area	Amount [ng/ul]	Grp	Name
11.874	BB	6.59571	6.96307e-2	4.59264e-1		Ferulic Acid

Totals : 4.59264e-1

Signal 2: DAD1 B, Sig=280,4 Ref=off

RetTime [min]	Type	Area [mAU*s]	Amt/Area	Amount [ng/ul]	Grp	Name
16.111		-	-	-		Cinnamic Acid

Totals : 0.00000

1 Warnings or Errors :

Warning : Calibrated compound(s) not found

===

*** End of Report ***

===

Acq. Operator : SYSTEM   Seq. Line : 11
Sample Operator : SYSTEM
Acq. Instrument : LC   Location : P1-B-01
Injection Date : 10/2/2024 2:01:34 PM   Inj : 1
Inj Volume : 5.000 µl
Different Inj Volume from Sample Entry! Actual Inj Volume : 10.000 µl
Acq. Method : C:\Users\Public\Documents\ChemStation\1\Data\R1Phenolics07122024\Kristin Sequence 2024-10-02 09-40-51\Ferulic Acid 300SB C18.M
Last changed : 10/2/2024 9:37:39 AM by SYSTEM
Analysis Method : C:\Users\Public\Documents\ChemStation\1\Data\R1Phenolics07122024\Kristin Sequence 2024-10-02 09-40-51\Ferulic Acid 300SB C18.M (Sequence Method)
Last changed : 10/3/2024 8:35:21 AM by SYSTEM
(modified after loading)

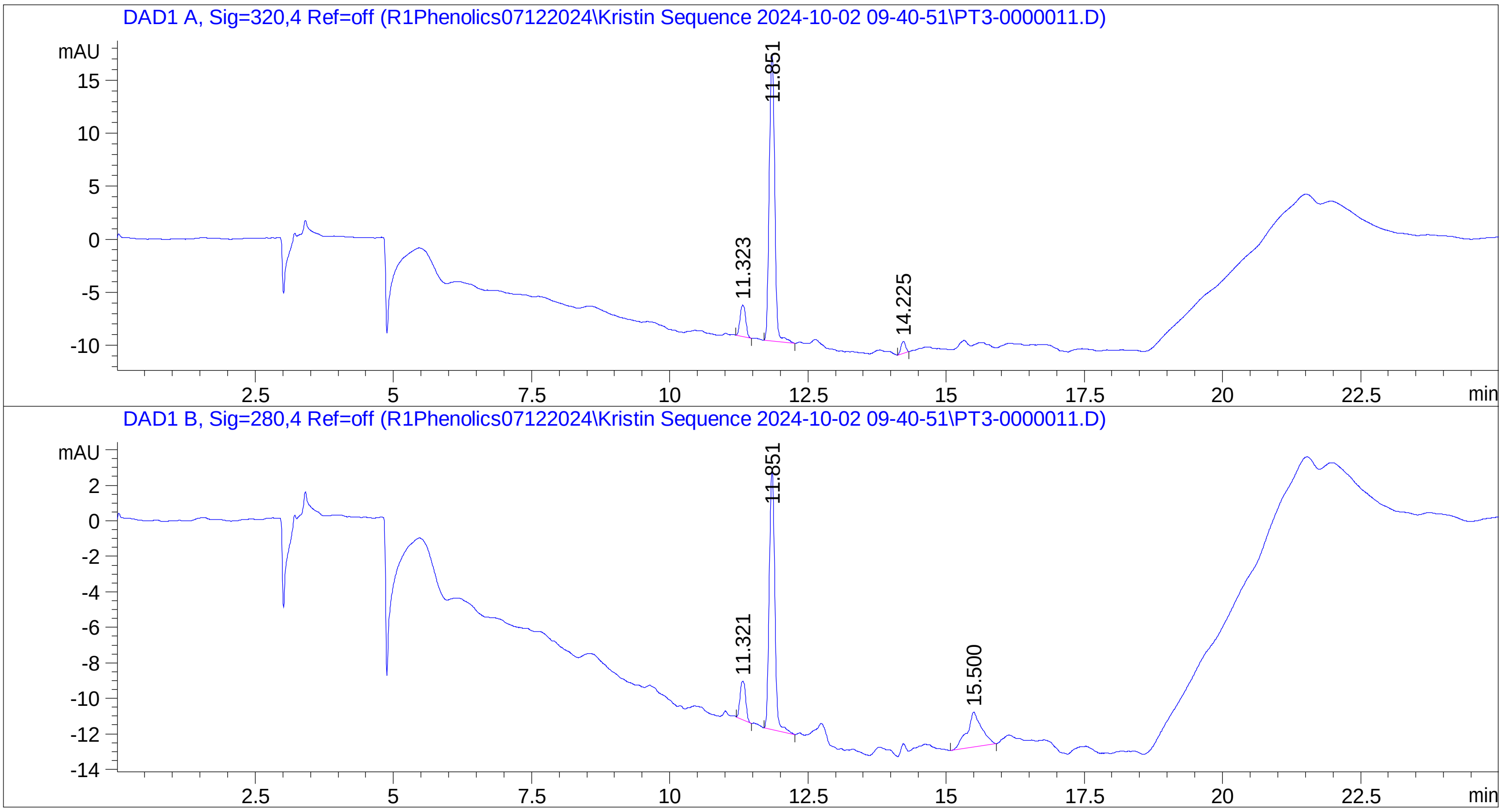

===

Fraction Information

===

No Fractions found.

===

===

===

External Standard Report

===

Sorted By : Signal
Calib. Data Modified : Thursday, October 3, 20248:35:21 AM
Multiplier : 1.0000
Dilution : 1.0000
Do not use Multiplier & Dilution Factor with ISTDs

Signal 1: DAD1 A, Sig=320,4 Ref=off

RetTime [min]	Type	Area [mAU*s]	Amt/Area	Amount [ng/ul]	Grp	Name
11.851	BB	186.57904	3.03544e-2	5.66349		Ferulic Acid

Totals : 5.66349

Signal 2: DAD1 B, Sig=280,4 Ref=off

RetTime [min]	Type	Area [mAU*s]	Amt/Area	Amount [ng/ul]	Grp	Name
16.111		-	-	-		Cinnamic Acid

Totals : 0.00000

1 Warnings or Errors :

Warning : Calibrated compound(s) not found

*** End of Report ***

Data File C:\USERS\P...HENOLICS07122024\KRISTIN SEQUENCE 2024-10-02 09-40-51\PT3-0000012.D
Sample Name: FAX 7

===

Acq. Operator : SYSTEM   Seq. Line : 12
Sample Operator : SYSTEM
Acq. Instrument : LC   Location : P1-B-02
Injection Date : 10/2/2024 2:27:32 PM   Inj : 1
Inj Volume : 5.000 µl
Different Inj Volume from Sample Entry! Actual Inj Volume : 10.000 µl
Acq. Method : C:\Users\Public\Documents\ChemStation\1\Data\R1Phenolics07122024\Kristin Sequence 2024-10-02 09-40-51\Ferulic Acid 300SB C18.M
Last changed : 10/2/2024 9:37:39 AM by SYSTEM
Analysis Method : C:\Users\Public\Documents\ChemStation\1\Data\R1Phenolics07122024\Kristin Sequence 2024-10-02 09-40-51\Ferulic Acid 300SB C18.M (Sequence Method)
Last changed : 10/3/2024 8:35:21 AM by SYSTEM
(modified after loading)

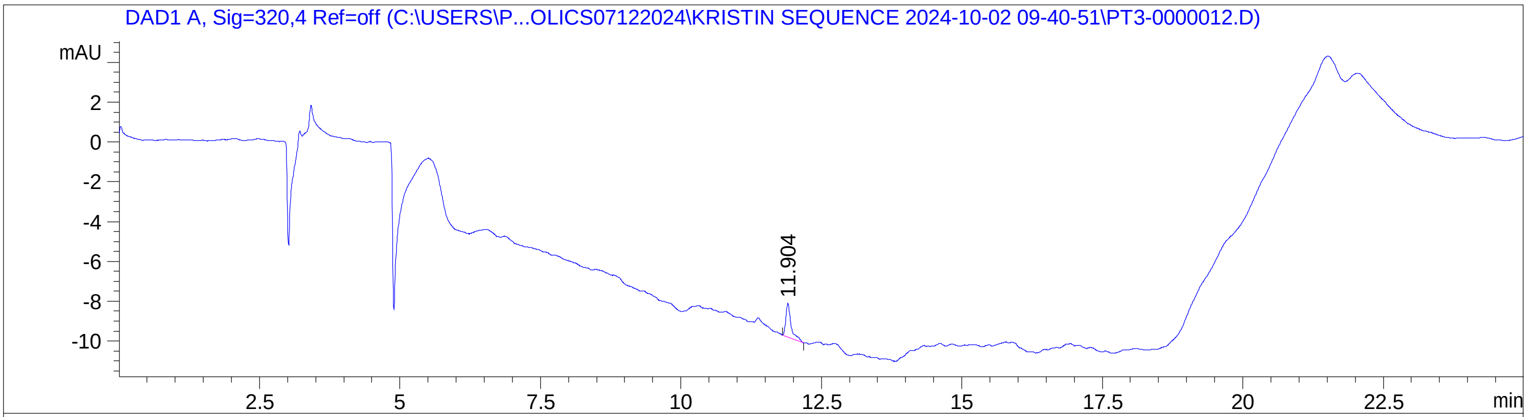

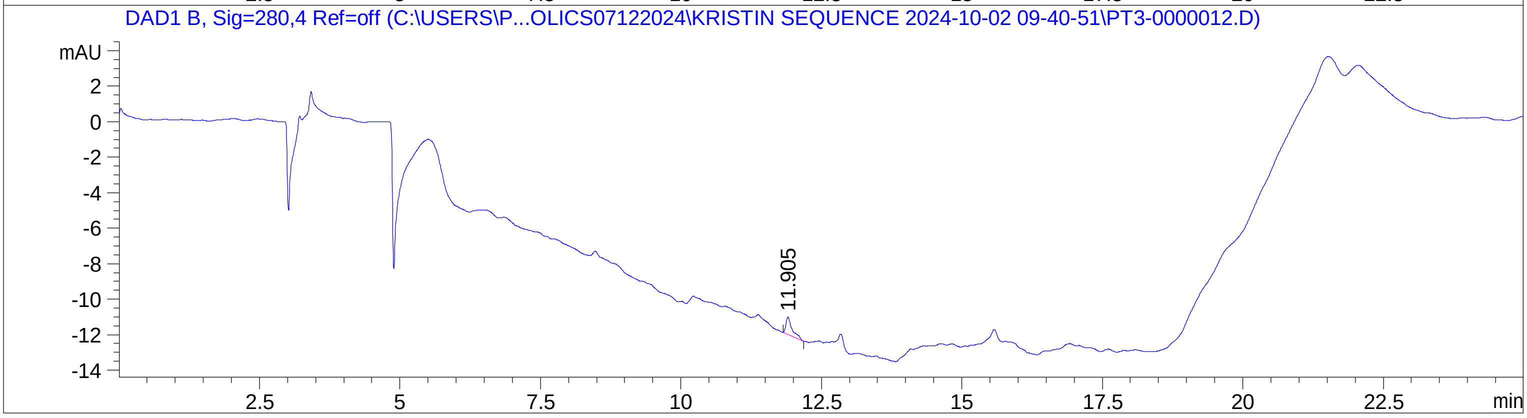

===

Fraction Information

===

No Fractions found.

===

===

===

External Standard Report

===

Sorted By : Signal
Calib. Data Modified : Thursday, October 3, 20248:35:21 AM
Multiplier : 1.0000
Dilution : 1.0000
Do not use Multiplier & Dilution Factor with ISTDs

Signal 1: DAD1 A, Sig=320,4 Ref=off

RetTime [min]	Type	Area [mAU*s]	Amt/Area	Amount [ng/ul]	Grp	Name
11.904	BB	10.89067	5.35736e-2	5.83453e-1		Ferulic Acid

Totals : 5.83453e-1

Signal 2: DAD1 B, Sig=280,4 Ref=off

RetTime [min]	Type	Area [mAU*s]	Amt/Area	Amount [ng/ul]	Grp	Name
16.111		-	-	-		Cinnamic Acid

Totals : 0.00000

1 Warnings or Errors :

Warning : Calibrated compound(s) not found

===

*** End of Report ***

===

```
Acq. Operator   : SYSTEM                          Seq. Line :  13
Sample Operator : SYSTEM
Acq. Instrument : LC                               Location :   P1-B-03
Injection Date  : 10/2/2024 2:53:29 PM                   Inj :   1
                                                 Inj Volume : 5.000 µl
Different Inj Volume from Sample Entry!  Actual Inj Volume : 10.000 µl
Acq. Method     : C:\Users\Public\Documents\ChemStation\1\Data\R1Phenolics07122024\Kristin
                  Sequence 2024-10-02 09-40-51\Ferulic Acid 300SB C18.M
Last changed    : 10/2/2024 9:37:39 AM by SYSTEM
Analysis Method : C:\Users\Public\Documents\ChemStation\1\Data\R1Phenolics07122024\Kristin
                  Sequence 2024-10-02 09-40-51\Ferulic Acid 300SB C18.M (Sequence Method)
Last changed    : 10/3/2024 8:35:21 AM by SYSTEM
                  (modified after loading)
```

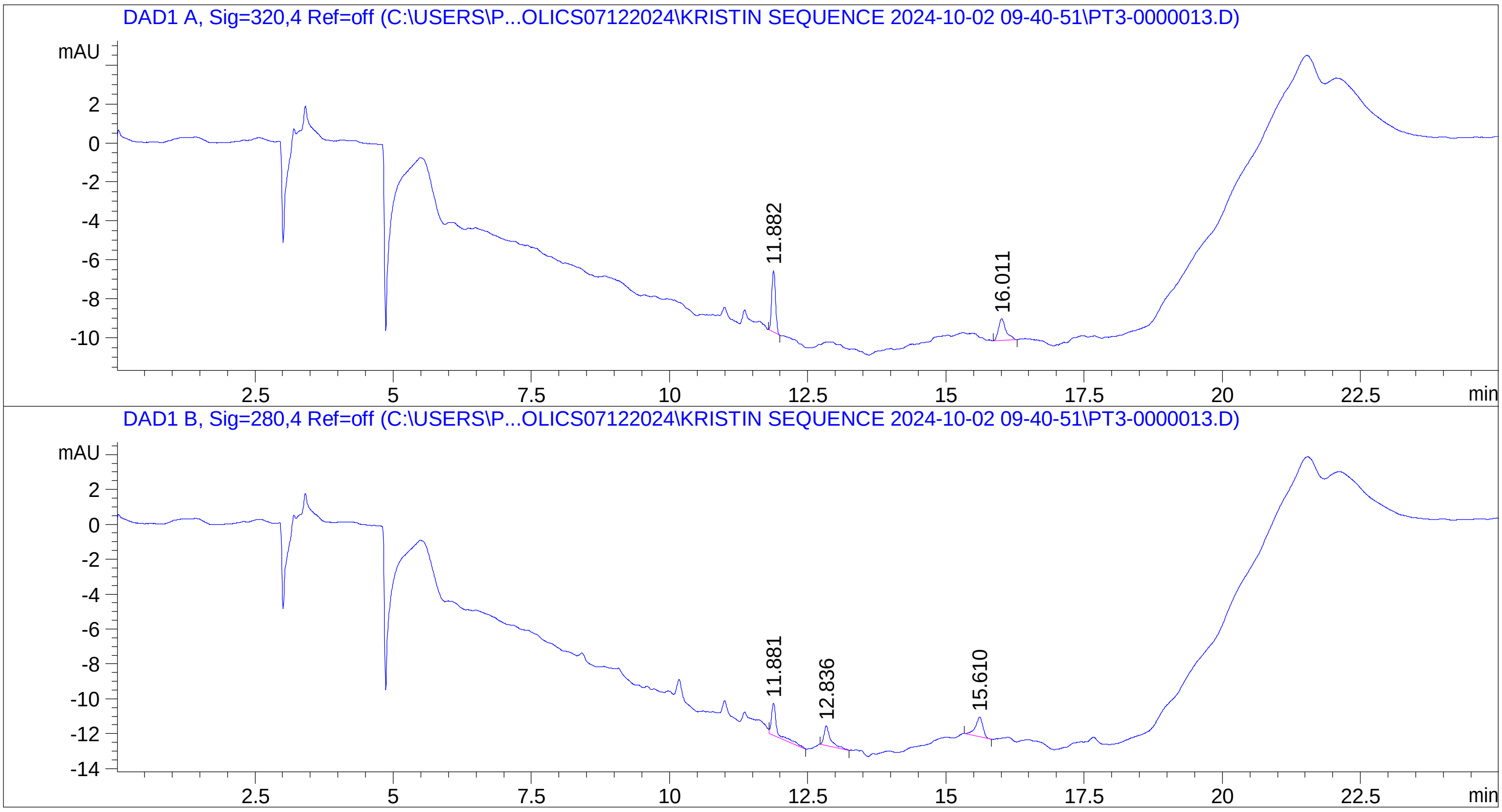

===

Fraction Information

===

No Fractions found.

===

===

===

External Standard Report

===

```
Sorted By             :      Signal
Calib. Data Modified  :      Thursday, October 3, 20248:35:21 AM
Multiplier            :      1.0000
Dilution              :      1.0000
Do not use Multiplier & Dilution Factor with ISTDs
```

Signal 1: DAD1 A, Sig=320,4 Ref=off

RetTime [min]	Type	Area [mAU*s]	Amt/Area	Amount [ng/ul]	Grp	Name
11.882	BB	15.06653	4.67392e-2	7.04198e-1		Ferulic Acid

Totals : 7.04198e-1

Signal 2: DAD1 B, Sig=280,4 Ref=off

RetTime [min]	Type	Area [mAU*s]	Amt/Area	Amount [ng/ul]	Grp	Name
16.111		-	-	-		Cinnamic Acid

Totals : 0.00000

1 Warnings or Errors :

Warning : Calibrated compound(s) not found

===

*** End of Report ***

Data File C:\USERS\P...HENOLICS07122024\KRISTIN SEQUENCE 2024-10-02 09-40-51\PT3-0000014.D
Sample Name: FAX 9

===

```
Acq. Operator   : SYSTEM                          Seq. Line :  14
Sample Operator : SYSTEM
Acq. Instrument : LC                               Location :   P1-B-04
Injection Date  : 10/2/2024 3:19:27 PM                  Inj :   1
                                                 Inj Volume : 5.000 µl
Different Inj Volume from Sample Entry!  Actual Inj Volume : 10.000 µl
Acq. Method     : C:\Users\Public\Documents\ChemStation\1\Data\R1Phenolics07122024\Kristin
                  Sequence 2024-10-02 09-40-51\Ferulic Acid 300SB C18.M
Last changed    : 10/2/2024 9:37:39 AM by SYSTEM
Analysis Method : C:\Users\Public\Documents\ChemStation\1\Data\R1Phenolics07122024\Kristin
                  Sequence 2024-10-02 09-40-51\Ferulic Acid 300SB C18.M (Sequence Method)
Last changed    : 10/3/2024 8:35:21 AM by SYSTEM
                  (modified after loading)
```

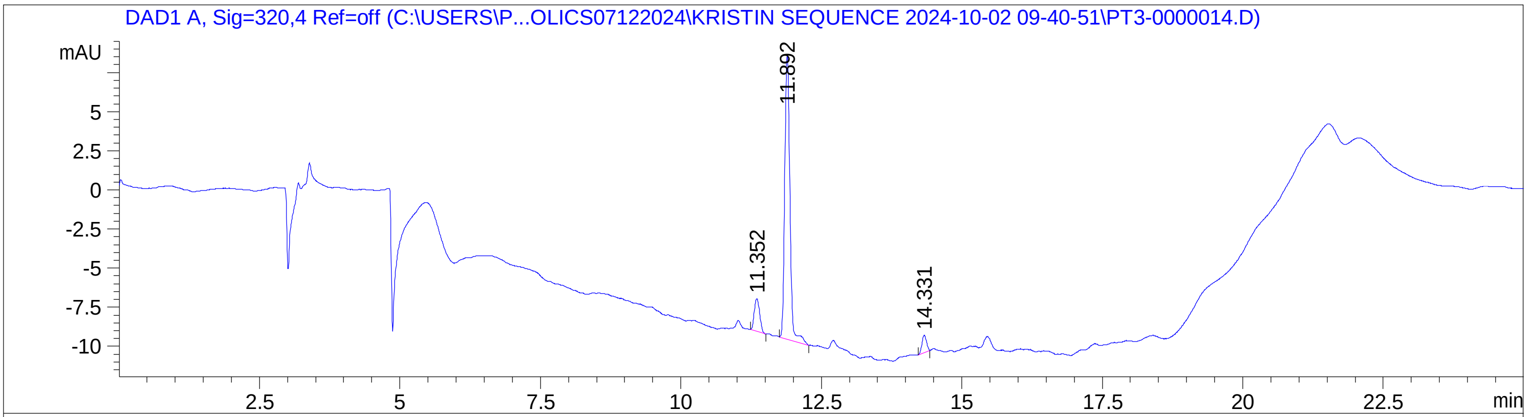

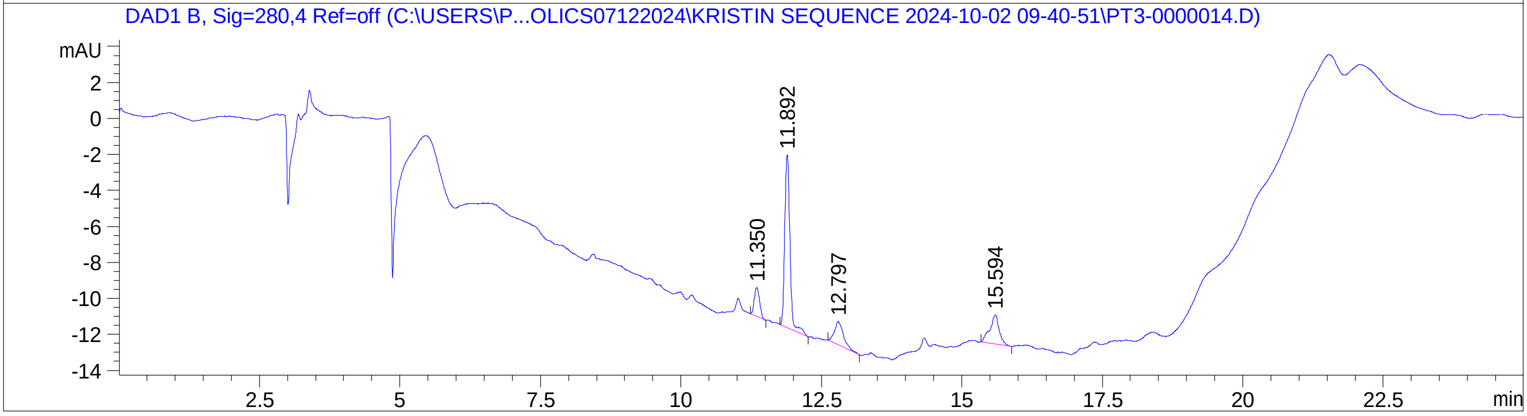

===

Fraction Information

===

No Fractions found.

===

===

===

External Standard Report

===

```
Sorted By             :      Signal
Calib. Data Modified  :      Thursday, October 3, 20248:35:21 AM
Multiplier            :      1.0000
Dilution              :      1.0000
Do not use Multiplier & Dilution Factor with ISTDs
```

Signal 1: DAD1 A, Sig=320,4 Ref=off

RetTime [min]	Type	Area [mAU*s]	Amt/Area	Amount [ng/ul]	Grp	Name
11.892	BB	116.39623	3.12222e-2	3.63415		Ferulic Acid

Totals : 3.63415

Signal 2: DAD1 B, Sig=280,4 Ref=off

RetTime [min]	Type	Area [mAU*s]	Amt/Area	Amount [ng/ul]	Grp	Name
16.111		-	-	-		Cinnamic Acid

Totals : 0.00000

1 Warnings or Errors :

Warning : Calibrated compound(s) not found

*** End of Report ***
